# Stereo- and regioselective hydroboration of 1-*exo*-methylene pyranoses: discovery of aryltriazolylmethyl C-galactopyranosides as selective galectin-1 inhibitors

**DOI:** 10.3762/bjoc.15.102

**Published:** 2019-05-07

**Authors:** Alexander Dahlqvist, Axel Furevi, Niklas Warlin, Hakon Leffler, Ulf J Nilsson

**Affiliations:** 1 Centre for Analysis and Synthesis, Department of Chemistry, Lund University, Box 124, SE-221 00 LUND, Sweden; 2 Division of Microbiology, Immunology and Glycobiology, Lund University, BMC C12, SE-221 84 LUND, Sweden

**Keywords:** C-galactoside, galectin-1, hydroboration, inhibition, selective, triazole

## Abstract

Galectins are carbohydrate recognition proteins that bind carbohydrates containing galactose and are involved in cell signaling and cellular interactions, involving them in several diseases. We present the synthesis of (aryltriazolyl)methyl galactopyranoside galectin inhibitors using a highly diastereoselective hydroboration of C1-*exo*-methylene pyranosides giving inhibitors with fourfold or better selectivity for galectin-1 over galectin-3, -4C (C-terminal CRD), -4N (N-terminal CRD), -7, -8C, -8N, -9C, and -9N and dissociation constants down to 170 µM.

## Introduction

Galectins are defined by a typically about 130 amino acid carbohydrate recognition domain (CRD) that binds to carbohydrates with at least one β-galactose subunit within a binding pocket large enough to accommodate a tetrasaccharide sequence of larger glycans [1–3]. Some galectins contain one CRD and occur as monomers or dimers, including galectins -1, -2, -7, -10 and -13 in humans. Others contain 2 different CRDs within the same peptide sequence and include galectins -4, -8, -9 and -12. Galectin-3 contains one CRD and a long N-terminal Pro/Gly-rich intrinsically disordered sequence [3–4]. The two most studied galectins, galectin-1 and -3, are present in a wide variety of tissues. Galectin-3 is almost ubiquitously expressed while galectin-1 is mainly expressed by immune cells, muscle cells, kidney cells and neurons [2–3]. Galectin-1 has a marked preference for binding N-linked glycans, while galectin-3 binds both O-linked and N-linked glycans [5]. Galectins play many biological roles in the body and one important and well-understood function is the cross-linking of cell-surface glycoproteins through binding to O and N-linked glycans on the cell surface. Surface proteins such as integrins [6–7], vascular endothelial growth factor receptor [8], and lysosome-associated membrane proteins [9] are known to be crosslinked by galectins, giving galectins a modulating role in cell adhesion, blood vessel growth and cellular uptake and breakdown, respectively. The association of galectins to cell signaling and adhesion gives them roles in several different pathological processes, such as pulmonary fibrosis [6,10], pathological lymphangiogenesis [11], inflammation [12] and cancer [13], with galectin inhibitors demonstrated to attenuate such processes [3,10–11,14]. Experimental galectin inhibitors have often been developed with a close resemblance to natural disaccharide ligands, such as lactose and *N*-acetyllactosamine (lacNAc) [15] via thiodigalactosides [16–19], as adding a second monosaccharide unit to the anomeric position of the minimal D-galactose ligand allows for an additional affinity-enhancing hydrogen bond according to structural and affinity analyses [20–21]. A complementary strategy has been to, instead of a second saccharide unit, add non-natural structural elements to a monogalactoside scaffold, as such derivatives have been hypothesized to allow for tuning of galectin selectivities and to be designed to have improved pharmacokinetic properties over natural saccharide fragments. Early reports along this strategy involved C-galactosides that were shown to reach affinities approaching those of lactose and LacNAc for galectin-3 and also to have selectivity over other galectin-1 [22–23]. Later, combining non-natural thiophenyl aglycons with C3-triazole groups at D-galactose led to the discovery of high affinity and selective galectin-3 inhibitors [24]. In this work, we present a synthesis pathway involving a diastereoselective hydroboration towards (aryltriazolyl)methyl galactopyranosyl derivatives and determined the viability of this as a scaffold for galectin inhibitors by screening a library of fourteen different products against galectins -1, -3, -4C (C-terminal CRD), -4N (N-terminal CRD), -7, -8C, -8N, -9C, and -9N.

## Results and Discussion

### Chemistry

**Scheme 1:**
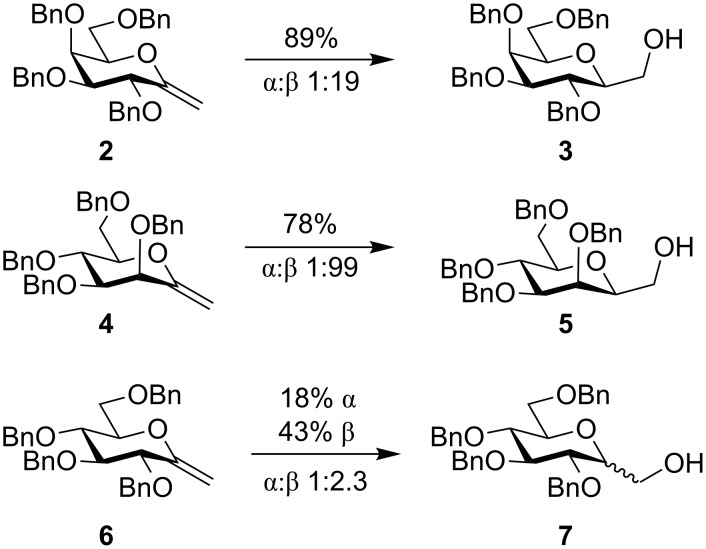
Diastereoselective hydroboration of glycopyranosyl exomethylene enol ethers **2**, **4**, and **6**: a) BH_3_-DMS, THF, 0 °C, 2 h; b) H_2_O_2_, NaOH, THF/water, 0 °C, 2 h.

The synthesis starts from the known enol ethers **2**, **4**, and **6** prepared using published methods [25–26]. Hydroborations of enol ethers have been known to give good to excellent regio- and stereoselectivity and are thus a possible route to 2-deoxyhepuloses **3**, **5** and **7** [27–30]. The hydroboration of enol ethers **2**, **4**, and **6** with borane dimethyl sulfide in THF, followed by oxidation using hydrogen peroxide and sodium hydroxide gave 2-deoxygalactoheptulose **3** and 2-deoxymannoheptulose **5** in good yields (89% and 78%) and with excellent diastereoselectivities (1:19 and 1:99 α:β ratio, respectively). Hydroboration of the glucose enol ether **6** afforded a mixture of both diastereomers (1:2.3 α:β ratio) in good yield (43% β and 18% α isolated yields, respectively). Identification of the diastereomers was accomplished by NOESY experiments using deuterated pyridine as a shift reagent solvent. Other hydroboration reagents, such as 9-borabicyclo[3.3.1]nonane (9-BBN) or pinacolborane, resulted in no conversion of **2** and an almost complete recovery of starting material in repeated experiments, although 9-BBN is known to convert the gluco analog **6** to β-**7** in high yield and with excellent stereoselectivity [31]. In none of the reaction conditions 1-methyl glycopyranoside side products were formed (Scheme 1).

**Scheme 2:**
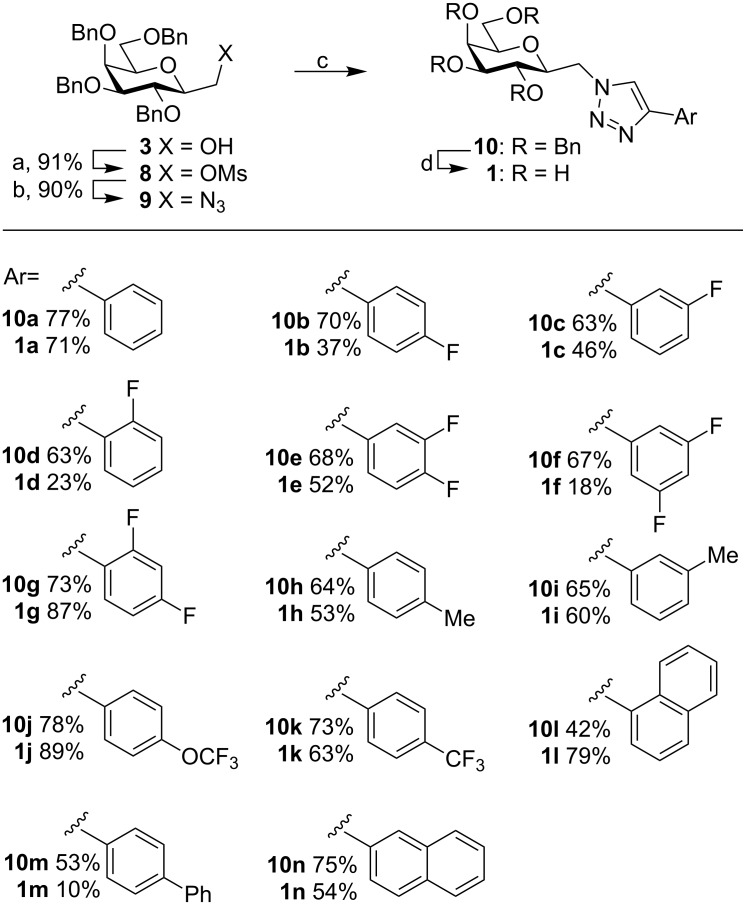
Synthesis of (aryltriazolyl)methylene galactopyranosides **1a**–**n**. Conditions: a) **3**, MsCl, pyridine, 0 °C, 2 h; b) NaN_3_, DMF, 95 °C, 12 h; c) alkyne, CuI, Et_3_N, MeCN, 12 h; d) Pd(OH)_2_/C, cyclohexene/EtOH, reflux, 12 h.

Continuing the synthesis towards (aryltriazolyl)methyl galactopyranosyl derivatives, the 2-deoxygalactoheptulose **3** was mesylated with mesyl chloride in pyridine at 0 °C to give 2-deoxy-1-mesylgalactoheptulose **8** (91%), followed by a nucleophilic substitution reaction with sodium azide in dimethylformamide to give the azide **9** in good yield (90%) [26]. The azide **9** was reacted with a panel of substituted ethynyl arenes to give (aryltriazolyl)methyl galactopyranosyls **10a**–**n** in fair to good yields (42–78% yields) via a copper-catalyzed Huisgen cycloaddition [17,32]. The resulting (aryltriazolyl)methyl galactopyranosyls **10a**–**n** were debenzylated using palladium hydroxide on carbon in a 2:1 cyclohexene and ethanol mixture to give **1a**–**n** in yields varying from poor to good (10–87% yields, Scheme 2). The transfer hydrogenation was selected as common hydrogenation using conditions with hydrogen gas and palladium on carbon lead to very low yields or no recovered product during the synthesis of **1a**,**b**. Unfortunately, no arenes bearing halogen substituents other than fluorine could be prepared, as they were dehalogenated during the final debenzylation.

### Galectin binding

Galectin-1, -3, -4C (C-terminal CRD), -4N (N-terminal CRD), -7, -8C, -8N, -9C, and -9N affinities for (aryltriazolyl)methyl galactopyranosyls **1a**–**n** were determined in a competitive fluorescence polarization titration assay [6,15–18,33]. Compounds **1a**–**n** had millimolar or higher affinities for all galectins except for galectins-1 and -3. Generally, compounds based on the (aryltriazolyl)methyl galactopyranoside scaffold were selective for galectin-1, with the best ligand (4-fluorophenyltriazolyl)methyl galactopyranoside **1b** having an affinity of 170 ± 2 µM and fourfold better than for galectin-3 (Table 1). The (4-(trifluoromethyl)phenyltriazolyl)methyl galactopyranoside **1k** displayed a comparable affinity of 240 ± 61 µM, but slightly lower selectivity over galectin-3. (1-Naphthyltriazolyl)methyl galactopyranoside **1l** also displayed a good affinity of 180 ± 20 µM for galectin-1 with a threefold selectivity over galectin-3. Methyl substituents gave ligands (**1h**,**i**) with poor galectin-1 affinity and undetectable galectin-3 binding, rendering them ineffective as galectin inhibitors. The contrast between the 4-methylphenyl **1h** and the 4-trifluoromethylphenyl **1k** is interesting, suggesting that the affinity of **1k** may be explained by fluorine interaction(s) and/or solvation effects. Of particular interest is the different difluorinated aryls **1e**–**g**, which show a reversed selectivity pattern. They display twofold preference for galectin-3 (**1g**), but their poor affinities limit their use as effective galectin-3 inhibitors. Furthermore, the 3-fluoro and 2-fluoro derivatives **1c** and **1d**, respectively also show a reversed galectin selectivity, but with only about twofold selectivity and poor affinity. The most potent galectin-1 inhibitor **1b** has at least fiftyfold better affinity than the reference monosaccharide methyl β-D-galactoside (**11**) and a similar affinity as the reference disaccharide methyl β-lactoside (**12**). Hence, the 4-fluorophenyltriazol moiety of **1b** efficiently replaces the galectin-1-interacting glucose unit in methyl β-lactoside (**12**) and at the same time induces a significantly better selectivity than that of methyl β-lactoside (**12**). Taken together, the 4-fluorophenyltriazole **1b** represents the most potent mono-galactoside-derived galectin-1 inhibitor, albeit with an apparently lower selectivity than reported C-galactosides [22–23]. Monosaccharide derivatives with somewhat higher affinity for galectin-1 are known; dissociation constants down to 62 µM and selectivity of 8 over galectin-3 [34]. However, these inhibitors are larger and carry double modifications with an anomeric thiophenyl aglycon combined with a heteroaryltriazole moiety at galactose C3 that both form affinity and selectivity-enhancing interactions with galectin-1. Unfortunately, attempts to combine the anomeric 4-fluorophenyltriazolylmethyl moiety of compound **1b** with such a galactose C3-thiazolyltriazole substituent reported to enhance affinity for galectin-1 [34] failed in our hands, why other additional substituents to combine with the 4-fluorophenyltriazolylmethyl moiety of compound **1b** remain to be discovered.

**Table 1:** Dissociation constants (*K*_D_ ± SEM in µM) and selectivities of **1a**–**n** and references methyl β-D-galactopyranoside (**11**) and methyl β-lactoside (**12**) binding to galectin-1 and -3 determined in a competitive fluorescence polarization assay [33].

	Galectin-		Selectivity
		
	1	3	Galectin-1/3

**1a**	600 ± 48	870 ± 37	1.5
**1b**	170 ± 2	710 ± 36	4.2
**1c**	1200 ± 120	720 ± 64	0.6
**1d**	390 ± 38	500 ± 23	1.3
**1e**	860 ± 150	610 ± 160	0.7
**1f**	1100 ± 43	790 ± 48	0.7
**1g**	1100 ± 16	490 ± 55	0.4
**1h**	1000 ± 130	>3000	>3
**1i**	990 ± 23	>3000	>3
**1j**	500 ± 59	700 ± 52	1.4
**1k**	240 ± 61	830 ± 38	3.5
**1l**	180 ± 20	470 ± 95	2.6
**1m**	710 ± 12	1200 ± 11	1.7
**1n**	1500 ± 290	1700 ± 190	1.1
**11** [35]	>10000	4400	0.4
**12** [35]	190	220	1.2

### Molecular modelling

**Figure 1:**
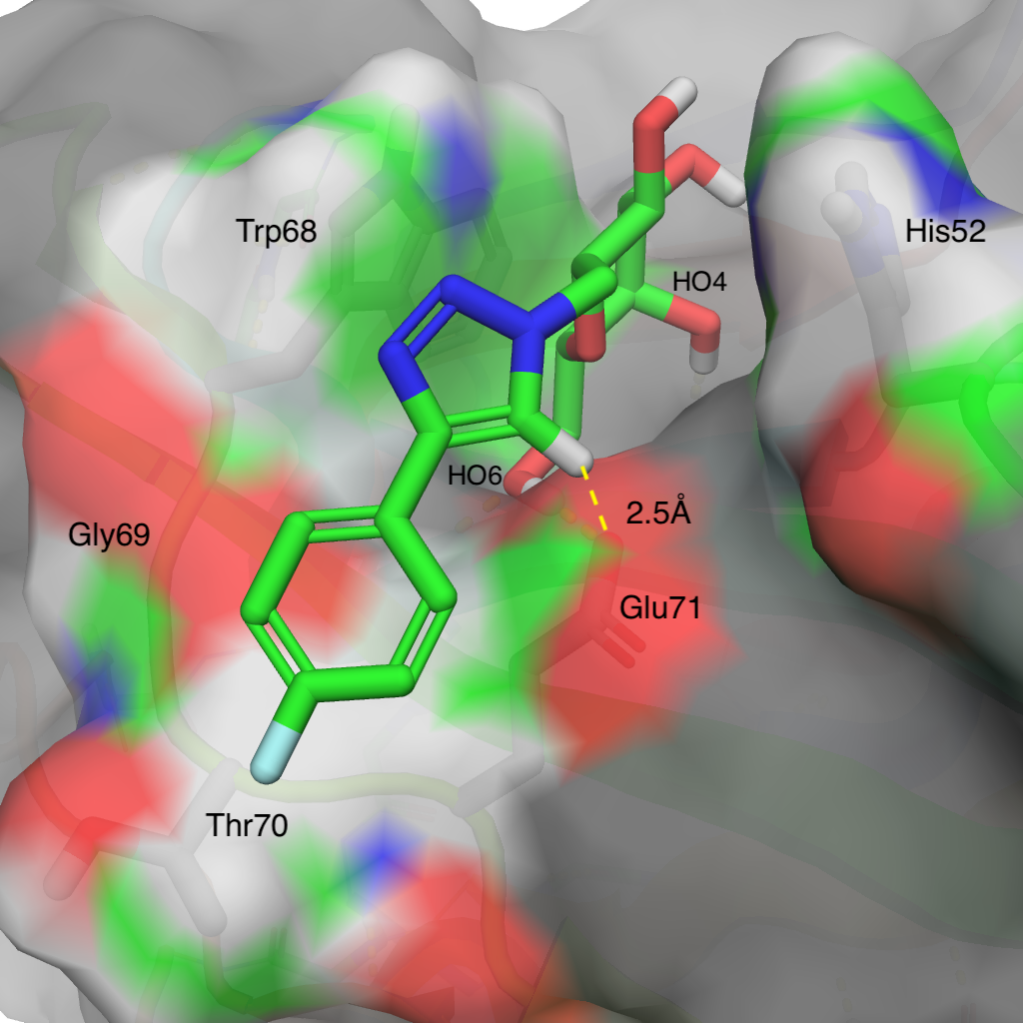
Galectin-1 in complex with **1b** derived by energy-minimizing a representative snapshot from a 200 ns molecular dynamic simulation. The distance between the triazole hydrogen and the Glu71 carboxylate is depicted with a yellow dashed line. The image was generated using PyMOL v1.7 (Schrodinger LLC).

In order to gain understanding of the affinity-enhancing effects of the aryltriazolyl C-galactoside aglycons, we performed a 200 ns molecular dynamic simulation of **1b** in the complex with galectin-1 and galectin-3. Starting conformations were selected with the galactopyranose of **1b** positioned overlapping with the positions of the lactose or *N*-acetyllactosamine galactopyranoses in galectin-1 (pdb id 1GZW) and galectin-3 (pdb id 1KJL), respectively, and with the 4-fluorophenyltriazol ring protruding away from the protein. The simulations with **1b** and galectin-1 converged toward a complex geometry in which the 4-fluorophenyltriazole extended along a shallow groove formed by Trp68-Gly69-Thr70-Glu71, while complex geometries in which the **1b** 4-fluorophenyltriazole interacted with the His52 that is situated above the β-face of the bound galactopyranose ring were less populated (Figure 1). The preferred complex geometry with the 4-fluorophenyltriazole extending along the Trp68-Gly69-Thr70-Glu71 groove may, in addition to a more favorable steric complementarity, benefit from the electron-poor triazole hydrogen sampling positions close to the Glu71 carboxylate and the electron-rich triazole nitrogens sampling positions close to the rim of Trp68 side chain. This hypothesis may also explain the weaker affinity by the corresponding 2-fluorophenyltriazole analogue **1d**, because in the favored complex geometry of **1b** introduction of a 2-fluoro atom would lead to this atom being close to either the Glu71 carboxylate or the triazole N3 lone pair.

MD simulations with **1b** positioned in a similar manner in the complex with galectin-3 converged to a similar complex geometry with the 4-fluorophenyltriazole rings extending along a shallow, but somewhat wider groove formed by the corresponding galectin-3 side chains Trp181-Gly182-Arg183-Glu184, which may explain why several of the aryltriazoles **1** also possess enhanced affinity for galectin-3 as compared to the reference methyl galactoside (**11**). Although the binding groove interacting with the ligand 4-fluorophenyl moiety is somewhat wider in galectin-3, analyses of factors underlying the 4-fold galectin-1 selectivity remain inconclusive.

## Conclusion

Hydroboration of 1-*exo*-methylene pyranosides **2** and **4** gives 3,4,5,7-tetra-*O*-benzyl-2-deoxy-D-galactoheptulose (**3**) and 3,4,5,7-tetra-*O*-benzyl-2-deoxy-D-mannoheptulose (**5**) in good yields and with excellent diastereoselectivities. This opens a quick, high-yielding route to (aryltriazolyl)methyl galactopyranosyls **1a**–**n**, a majority of which are galectin-1 selective with dissociation constants down to 170 ± 2 µM (**1b**) and fourfold or better selectivity for galectin-1 over other galectins. This is comparable to or better than known C-galactoside based galectin-1 inhibitors with almost a factor of two [22–23]. Hence, one monosaccharide moiety, glucose/*N*-acetylglucosamine of the common disaccharide ligands lactose/*N*-acetyllactosamine has effectively been replaced with an aryltriazole motif, which is chemically wholly dissimilar and more selective. This opens a route towards galectin inhibitors with improved selectivities and potentially better pharmacokinetic properties than existing inhibitors based on lactose- and thiodigalactoside or other disaccharide core structures.

## Experimental

### General procedures

Chemicals were obtained from Sigma-Aldrich unless otherwise stated and used without further purification, unless stated in the procedure. NMR spectra were collected on a Bruker Ultrashield Plus/Avance II 400 MHz spectrometer. ^1^H NMR spectra were recorded at 400 MHz and ^13^C NMR spectra at 100 MHz with residual solvent signals as references. ^19^F NMR spectra were recorded at 376 MHz. Stereochemistry was assigned through NOESY using pyridine-*d*_5_ as a shift reagent solvent. All final compounds were purified using preparative HPLC on an Agilent 1260 Infinity system with a SymmetryPrep C18 5 µM 19 × 100 mm column using a gradient (water with 0.1% formic acid and acetonitrile); 0–20 min 10–100% acetonitrile, 20–23 min 100% acetonitrile. Monitoring and collection based on UV–vis absorbance at 210 nm and 254 nm, respectively. Purity analysis was performed using UPLC–MS with UV–vis detection on a Waters Acquity UPLC + Waters XEVO-G2 system using a Waters Acquity CSH C18, 1.7 µm, 2.1 × 100 mm column. Samples were run using a gradient with water (0.1% formic acid) and acetonitrile using a flow rate of 0.50 mL/min and a column temperature 60 °C. Gradient parameters: 0–0.7 min: 40% acetonitrile, 0.7–10.0 min: 40–99% acetonitrile, 10.0–11.0 min 99% acetonitrile, 11.0–11.1 min 99–40% acetonitrile, 11.1–13 min 40% acetonitrile, 3 or 6 µL injection, detection 190–300 nm. MS parameters: Cap voltage 3.0 kV, cone voltage 40 kV, Ext 4, source temperature 120 °C, desolvation temperature 500 °C, cone gas 50, desolvation gas 800, centroid resolution mode, *m*/*z* interval 50–1200, lockspray. Calibration: Leu-enkephalin *m*/*z* 556.2771, 0.25 s every 30 s, average 3. For optical rotation measurements, samples were dissolved in an appropriate solvent to a concentration of 2–10 mg/mL. Polarimetry was performed on a PerkinElmer Model 341 Polarimeter using a sodium lamp and measuring at 589 nM with a 90 mm long 1 mL cell at 20 °C. For infrared spectroscopy, samples were pelleted with potassium bromide and analyzed on a Shimadzu FTIR-84005.

**1,2-Dideoxy-1-(4-phenyl-1*H*-1,2,3-triazol-1-yl)-β-D-galactoheptulose (1a):** Compound **10a** (42 mg, 0.062 mmol) was dissolved in cyclohexene/ethanol 2:1 (1.5 mL), palladium(II) hydroxide on charcoal (17 mg, 20 wt %) was added and the mixture refluxed overnight at 80 °C. The reaction mixture was diluted with methanol (15 mL), filtered through Celite, evaporated, purified by column chromatography (5:1, dichloromethane/methanol), and then preparative HPLC (gradient from 10% acetonitrile/90% water with 0.1% formic acid to 100% acetonitrile over 20 min, followed by 3 min of 100% acetonitrile) to give **1a** (14 mg, 71%) as a white powder. [α]_D_^20^ 24 (*c* 0.6, methanol); ^1^H NMR (400 MHz, CD_3_OD) δ 8.42 (s, 1H), 7.86–7.81 (m, 2H), 7.48–7.42 (m, 2H), 7.38–7.33 (m, 1H), 4.98–4.95 (m, 1H), 4.57 (dd, *J =* 14.4 Hz, 7.5 Hz, 1H, H1), 3.91–3.88 (m, 1H, H5), 3.78 (dd, *J =* 10.8 Hz, 7.35 Hz, 1H, H7), 3.70 (dd, *J =* 12.1 Hz, 4.8 Hz, 1H, H7), 3.60–3.48 (m, 4H); ^13^C NMR (100 MHz, CD_3_OD) δ 128. 5, 127.9, 125.3, 122.3, 78.9, 78.7, 74.7, 69.5, 68.4, 61.5, 51.5; HRMS (*m*/*z*): [M + H]^+^ calcd for 322.1403; found, 322.1402; purity by HPLC, UV–vis detection: 98.4%.

**1,2-Dideoxy-1-[4-(4-fluorophenyl)-1*H*-1,2,3-triazol-1-yl]-β-D-galactoheptulose (1b):** Compound **10b** (50 mg, 0.147 mmol) was dissolved in cyclohexene/ethanol 2:1 (3 mL), palladium(II) hydroxide on charcoal (40 mg, 20 wt %) was added, and the mixture refluxed overnight at 80 °C. The reaction mixture was diluted with methanol (15 mL), filtered through Celite, evaporated, purified by column chromatography (dichloromethane/methanol 5:1), and then preparative HPLC (gradient from 10% acetonitrile/90% water with 0.1% formic acid to 100% acetonitrile over 20 min, followed by 3 min of 100% acetonitrile) to give **1b** (9 mg, 37%) as a white powder. [α]_D_^20^ 17 (*c* 0.7, CD_3_OD); ^1^H NMR (400 MHz, CD_3_OD) δ 8.43 (s, 1H), 7.88–7.82 (m, 2H), 7.24–7.16 (m, 2H), 4.95 (dd, *J =* 14.36 Hz, 2.0 Hz, 1H, H1), 4.58 (dd, *J =* 13.6, 7.2 Hz, H1), 3.89 (dd, *J =* 2.9 Hz, 0.9 Hz, 1H, H5), 3.78 (dd, *J =* 11.4 Hz, 7.2 Hz, 1H, H7), 3.69 (dd, *J =* 11.5 Hz, 4.6 Hz, 1H, H7), 3.60–3.49 (m, 4H); ^13^C NMR (100 MHz, CD_3_OD) δ 161.6, 146.2, 127.4, 127.3, 126.6, 122.4, 115.5, 115.3, 79.0, 78.6, 74.7, 69.4, 68.4, 61.5, 51.6; ^19^F NMR (376 MHz, CD_3_OD) δ −115.54; HRMS (*m*/*z*): [M + H]^+^ calcd for 340.1306; found, 340.1309; purity by HPLC, UV–vis detection: 95.6%.

**1,2-Dideoxy-1-[4-(3-fluorophenyl)-1*H*-1,2,3-triazol-1-yl]-β-D-galactoheptulose (1c):** Compound **10c** (71 mg, 0.102 mmol) was dissolved in cyclohexene/ethanol 2:1 (4.5 mL), 27 mg of 20 wt % palladium(II) hydroxide on charcoal was added, and the mixture refluxed overnight at 80 °C. The reaction mixture was diluted with methanol (15 mL), filtered through Celite, evaporated, and purified by column chromatography (dichloromethane/methanol 5:1), and then preparative HPLC (gradient from 10% acetonitrile/90% water with 0.1% formic acid to 100% acetonitrile over 20 min, followed by 3 min of 100% acetonitrile) to give **1c** (16 mg, 46%) as a white powder. [α]_D_^20^ 19 (*c* 0.6, CD_3_OD); ^1^H NMR (400 MHz, CD_3_OD) δ 8.47 (s, 1H), 7.68–7.64 (m, 1H), 7.62–7.57 (m, 1H), 7.50–7.43 (m, 1H), 7.12–7.06 (m, 1H), 4.95 (dd, *J =* 14.4 Hz, 1.9 Hz, 1H, H1), 4.58 (dd, *J =* 14.2 Hz, 7.7 Hz, 1H, H1), 3.90 (dd, *J =* 2.9 Hz, 0.9 Hz, 1H, H5), 3.79 (dd, *J =* 11.2 Hz, 6.9 Hz, 1H, H7), 3.70 (dd, *J =* 11.6 Hz, 4.71 Hz, 1H, H7), 3.60–3.48 (m, 4H); ^13^C NMR (100 MHz, CD_3_OD) δ 164.5, 146.2, 132.9, 130.5, 122.9, 121.1, 114.4, 111.9, 78.9, 78.7, 74.7 69.4, 68.4, 61.5, 51.5; ^19^F NMR (376 MHz, CD_3_OD) δ −114.83; HRMS (*m*/*z*): [M + H]^+^ calcd for 340.1314; found, 340.1309; purity by HPLC, UV–vis detection: 99.6%.

**1,2-Dideoxy-1-[4-(2-fluorophenyl)-1*H*-1,2,3-triazol-1-yl]-β-D-galactoheptulose (1d):** Compound **10d** (66 mg, 0.095 mmol) was dissolved in cyclohexene/ethanol 2:1 (4.5 mL), palladium(II) hydroxide on charcoal (25 mg, 20 wt %) was added, and the mixture refluxed overnight at 80 °C. The reaction mixture was diluted with methanol (15 mL), filtered through Celite, evaporated, purified by column chromatography (dichloromethane/methanol 5:1), and then preparative HPLC (gradient from 10% acetonitrile/90% water with 0.1% formic acid to 100% acetonitrile over 20 min, followed by 3 min of 100% acetonitrile) to give **1d** (8 mg, 23%) as a white powder. [α]_D_^20^ 27 (*c* 0.5, CD_3_OD); ^1^H NMR (400 MHz, CD_3_OD) δ 8.41 (d, *J =* 3.74 Hz, 1H), 8.11 (td, *J =* 7.6 Hz, 1.9 Hz, 1H), 7.43–7.37 (m, 1H), 7.30 (td, *J =* 7.6 Hz, 1.3 Hz, 1H), 7.24 (ddd, *J =* 11.4 Hz, 8.3 Hz, 1.1 Hz, 1H), 4.97 (dd, *J =* 14.4 Hz, 2.1 Hz, 1H, H1), 4.60 (dd, *J =* 13.9 Hz,7.6 Hz, 1H, H1), 3.92–3.89 (m, 1H), 3.75 (*J =* 11.3 Hz, 6.7 Hz, 1H, H7), 3.70 (dd, *J =* 11.3 Hz, 5.1 Hz, 1H, H7), 3.62–3.49 (m, 4H); ^13^C NMR (100 MHz, CD_3_OD) δ 158.0, 140.7, 129.5, 129.4, 127.3, 127.3, 124.9, 124.8, 124.38, 124.35, 118.4, 115.6, 115.4, 78.8, 78.7, 74.7, 69.3, 68.4, 61.2, 51.4; ^19^F NMR (376 MHz, CD_3_OD) δ −116.16; HRMS (*m*/*z*): [M + H]^+^ calcd for 340.1304; found, 340.1309; purity by HPLC, UV–vis detection: 98.5%.

**1,2-Dideoxy-1-[4-(3,4-difluorophenyl)-1*H*-1,2,3-triazol-1-yl]-β-D-galactoheptulose (1e):** Compound **10e** (66 mg, 0.092 mmol) was dissolved in cyclohexene/ethanol 2:1 (4.5 mL), palladium(II) hydroxide on charcoal (24 mg, 20 wt %) was added, and the mixture refluxed overnight at 80 °C. The reaction mixture was diluted with methanol (15 mL), filtered through Celite, evaporated, purified by column chromatography (dichloromethane/methanol 5:1), and then preparative HPLC (gradient from 10% acetonitrile/90% water with 0.1% formic acid to 100% acetonitrile over 20 min, followed by 3 min of 100% acetonitrile) to give **1e** (17 mg, 52%) as a white powder. [α]_D_^20^ 16 (*c* 0.8, CD_3_OD); ^1^H NMR (400 MHz, CD_3_OD) δ 8.44 (s, 1H), 7.76 (ddd, *J =* 11.7 Hz, 7.8 Hz, 2.2 Hz, 1H), 7.67–7.62 (m, 1H), 7.39–7.31 (m, 1H), 4.94 (dd, *J =* 14.3 Hz,1.9 Hz, 1H, H1), 4.57 (dd, *J =* 14.5 Hz, 7.3 Hz, 1H, H1), 3.89 (dd, *J =* 2.9 Hz, 1.1 Hz, 1H, H5), 3.78 (dd, *J =* 11.3 Hz, 7.4 Hz, 1H, H7), 3.70 (dd, *J =* 11.6 Hz, 4.7 Hz, 1H, H7), 3.59–3.49 (m, 4H); ^13^C NMR (100 MHz, CD_3_OD) δ 163.6, 157.1, 151.6, 151.1, 145.4, 128.1, 122.7, 121.9, 121.81, 121.79, 121.75, 117.6, 117.5, 114.3, 114.1, 79.0, 78.7, 74.7, 69.4, 68.4, 61.5, 51.5; ^19^F NMR (376 MHz, CD_3_OD) δ −139.97, −140.03, −141.26, −141.31; HRMS (*m*/*z*): [M + H]^+^ calcd for 358.1212; found, 358.1215; purity by HPLC, UV–vis detection: 98.2%.

**1,2-Dideoxy-1-[4-(3,5-difluorophenyl)-1*H*-1,2,3-triazol-1-yl]-β-D-galactoheptulose (1f):** Compound **10f** (81 mg, 0.113 mmol) was dissolved in cyclohexene/ethanol 2:1 (6 mL) with 2 drops of acetic acid, palladium(II) hydroxide on charcoal (30 mg, 20 wt %) was added and the mixture refluxed overnight at 80 °C. The reaction mixture was diluted with 15 mL methanol, filtered through Celite, evaporated, purified by column chromatography (dichloromethane/methanol 5:1), and then preparative HPLC (gradient from 10% acetonitrile/90% water with 0.1% formic acid to 100% acetonitrile over 20 min, followed by 3 min of 100% acetonitrile) to give **1f** (7 mg, 18%) as a white powder. [α]_D_^20^ 22 (*c* 0.8, CD_3_OD); ^1^H NMR (400 MHz, CD_3_OD) δ 8.51 (s, 1H), 7.51–7.44 (m, 2H), 6.98–6.91 (tt, *J =* 9.1 Hz, 2.3 Hz, 1H, phenyl H4), 4.95 (dd, *J =* 14.4 Hz, 2.0 Hz, 1H, H1), 4.57 (dd, *J =* 14.2 Hz, 7.5 Hz, 1H, H1), 3.89 (dd, *J =* 2.7 Hz, 1.2 Hz, 1H, H5), 3.79 (dd, *J =* 11.2 Hz, 7.3 Hz, 1H, H7), 3.70 (dd, *J =* 11.3 Hz, 4.7 Hz, 1H, H7), 3.59–3.47 (m, 4H); ^13^C NMR (100 MHz, CD_3_OD) δ 164.9, 162.3, 145.2, 123.4, 108.1, 107.8, 102.8, 102.6, 102.0, 79.0, 78.6, 74.7, 69.4, 68.4, 61.5, 51.5; ^19^F NMR (376 MHz, CD_3_OD) δ −111.27; HRMS (*m*/*z*): [M + H]^+^ calcd for 358.1219; found, 358.1215; purity by HPLC, UV–vis detection: 99.6%.

**1,2-Dideoxy-1-[4-(2,4-difluorophenyl)-1*H*-1,2,3-triazol-1-yl]-β-D-galactoheptulose (1g):** Compound **10g** (88 mg, 0.123 mmol) was dissolved in cyclohexene/ethanol 2:1 (6 mL), palladium(II) hydroxide on charcoal (69 mg, 20 wt %) was added and the mixture refluxed overnight at 80 °C. The reaction mixture was diluted with 15 mL methanol, filtered through Celite, evaporated, purified by column chromatography (dichloromethane/methanol 5:1), and then preparative HPLC (gradient from 10% acetonitrile/90% water with 0.1% formic acid to 100% acetonitrile over 20 min, followed by 3 min of 100% acetonitrile) to give **1g** (40 mg, 87%) as a clear solid. [α]_D_^20^ 7 (*c* 0.7, methanol); ^1^H NMR (400 MHz, CD_3_OD) δ 8.36 (d, *J =* 3.6 Hz, 1H), 8.14–8.07 (m, 1H), 7.13–7.05 (m, 2H), 4.95 (dd, *J =* 14.2 Hz, 2.0 Hz, 1H, H1), 4.60 (dd, *J =* 14.2 Hz, 7.6 Hz, 1H, H1), 3.91 (dd, *J =* 2.7 Hz,1.0 Hz, 1H, H5), 3.77–3.67 (m, 2H), 3.61–3.48 (m, 4H); ^13^C NMR (100 MHz, CD_3_OD) δ 163.6, 163.5, 161.6, 161.5, 160.3, 160.2, 158.3, 158.2, 139.91, 139.89, 128.5, 128.44, 128.40, 128.36, 124.5, 124.4, 115.0, 114.9, 114.84, 114.81, 111.58, 111.55, 111.40, 111.37, 103.9, 103.7, 103.5, 78.7, 78.6, 74.6, 70.6, 69.2, 68.3, 61.2, 60.7, 51.4; ^19^F NMR (376 MHz, CD_3_OD) δ −111.90, −112.17; HRMS (*m*/*z*): [M + H]^+^ calcd for 358.1211; found, 358.1215 calculated. Purity by HPLC, UV–vis detection: 96.6%.

**1,2-Dideoxy-1-[4-(4-methylphenyl)-1*H*-1,2,3-triazol-1-yl]-β-D-galactoheptulose (1h):** Compound **10h** (71 mg, 0.102 mmol) was dissolved in cyclohexene/ethanol 2:1 (4.5 mL), palladium(II) hydroxide on charcoal (27 mg, 20 wt %) was added, and the mixture refluxed overnight at 80 °C. The reaction mixture was diluted with methanol (15 mL), filtered through Celite, evaporated, purified by column chromatography (dichloromethane/methanol 5:1), and then preparative HPLC (gradient from 10% acetonitrile/90% water with 0.1% formic acid to 100% acetonitrile over 20 min, followed by 3 min of 100% acetonitrile) to give **1h** (18 mg, 53%) as a white powder. [α]_D_^20^ 19 (*c* 0.9, CD_3_OD); ^1^H NMR (400 MHz, CD_3_OD) δ 8.37 (s, 1H), 7.74–7.69 (m, 2H), 7.29–7.25 (m, 2H), 4.94 (dd, *J =* 14.2 Hz, 1.8 Hz, 1H, H1), 4.56 (dd, *J =* 14.1 Hz, 7.4 Hz, 1H, H1), 3.91–3.88 (m, 1H, H5), 3.78 (dd, *J =* 10.8 Hz, 7.3 Hz, 1H, H7), 3.70 (dd, *J =* 11.4 Hz, 4.8 Hz, 1H, H7), 3.60–3.48 (m, 4H), 2.39 (s, 3H); ^13^C NMR (100 MHz, CD_3_OD) δ 147.4, 137.9, 129.1, 127.6, 125.3, 121.9, 78.9, 78.8, 74.7, 69.4, 68.4, 61.5, 51.4, 19.9; HRMS (*m*/*z*): [M + H]^+^ calcd for 336.1558; found, 336.1559; purity by HPLC, UV–vis detection: 99.6%.

**1,2-Dideoxy-1-[4-(3-methylphenyl)-1*H*-1,2,3-triazol-1-yl]-β-D-galactoheptulose (1i):** Compound **10i** (71 mg, 0.102 mmol) was dissolved in cyclohexene/ethanol 2:1 (4.5 mL), palladium(II) hydroxide on charcoal (27 mg, 20 wt %) was added, and the mixture refluxed overnight at 80 °C. The reaction mixture was diluted with methanol (15 mL), filtered through Celite, evaporated, purified by column chromatography (dichloromethane/methanol 5:1), and then preparative HPLC (gradient from 10% acetonitrile/90% water with 0.1% formic acid to 100% acetonitrile over 20 min, followed by 3 min of 100% acetonitrile) to give **1i** (20 mg, 60%) as a white powder. [α]_D_^20^ 16 (*c* 1, CD_3_OD); ^1^H NMR (400 MHz, CD_3_OD) δ 8.40 (s, 1H), 7.66 (s, 1H), 7.62 (d, *J =* 7.8 Hz, 1H), 7.32 (t, *J =* 7.6 Hz, 1H), 7.18 (d, *J =* 7.6 Hz, 1H), 4.94 (dd, *J =* 14.3 Hz, 1.8 Hz, 1H, H1), 4.56 (dd, *J =* 14.3 Hz, 7.4 Hz, 1H, H1), 3.90–3.88 (m, 1H, H5), 3.78 (dd, *J =* 11.5 Hz, 7.3 Hz, 1H, H7), 3.70 (dd, *J =* 11.3 Hz, 4.6 Hz, 1H, H7), 3.60–3.50 (m, 4H), 2.41 (s, 3H); ^13^C NMR (100 MHz, CD_3_OD) δ 147.4, 138.4, 130.3, 128.6, 128.5, 125.9, 122.5, 122.2, 78.9, 78.8, 74.7, 69.4, 68.4, 61.5, 51.5, 20.1; HRMS (*m*/*z*): [M + H]^+^ calcd for 336.1553; found, 336.1559; purity by HPLC, UV–vis detection: 97.3%.

**1,2-Dideoxy-1-[4-(4-trifluoromethoxyphenyl)-1*H*-1,2,3-triazol-1-yl]-β-D-galactoheptulose (1j):** Compound **10j** (77.3 mg, 0.101 mmol) was dissolved in cyclohexene/ethanol 2:1 (4.5 mL), palladium(II) hydroxide on charcoal (28 mg, 20 wt %) was added, and the mixture was refluxed overnight at 80 °C. The reaction mixture was diluted with methanol (15 mL), filtered through Celite, evaporated, purified by column chromatography (dichloromethane/methanol 5:1), and then preparative HPLC (gradient from 10% acetonitrile/90% water with 0.1% formic acid to 100% acetonitrile over 20 min, followed by 3 min of 100% acetonitrile) to give **1j** (40 mg, 89%) as a white solid. [α]_D_^20^ 7 (*c* 0.9, MeOH); ^1^H NMR (400 MHz, CD_3_OD) δ 8.46 (s, 1H), 7.96–7.91 (m, 2H), 7.39–7.34 (m, 2H), 4.95 (dd, *J =* 14.1 Hz, 2.0 Hz, 1H, H1), 4.57 (dd, *J =* 14.0 Hz, 7.5 Hz, 1H, H1), 3.90–3.88 (m, 1H, H5), 3.78 (dd, *J =* 11.4 Hz, 7.3 Hz, 1H, H7), 3.70 (dd, *J =* 11.5 Hz, 4.9 Hz, 1H, H7), 3.60–3.49 (m, 4H); ^13^C NMR (100 MHz, CD_3_OD) δ 148.8, 145.9, 129.8, 126.9, 122.7, 121.2, 78.9, 78.7, 74.7, 69.4, 68.4, 61.5, 51.5; ^19^F NMR (376 MHz, CD_3_OD) δ −59.49; HRMS (*m*/*z*): [M + H]^+^ calcd for 406.1234; found, 406.1226; purity by HPLC, UV–vis detection: 99.8%.

**1,2-Dideoxy-1-[4-(4-trifluoromethylphenyl)-1*H*-1,2,3-triazol-1-yl]-β-D-galactoheptulose (1k):** Compound **10k** (51 mg, 0.067 mmol) was dissolved in cyclohexene/ethanol 2:1 (4.5 mL), palladium(II) hydroxide on charcoal (38 mg, 20 wt %) was added, and the mixture refluxed overnight at 80 °C. The reaction mixture was diluted with methanol (15 mL), filtered through Celite, evaporated, purified by column chromatography (dichloromethane/methanol 5:1), and then preparative HPLC (gradient from 10% acetonitrile/90% water with 0.1% formic acid to 100% acetonitrile over 20 min, followed by 3 min of 100% acetonitrile) to give **1k** (17 mg, 63%) as a white solid. [α]_D_^20^ 8 (*c* 0.7, MeOH); ^1^H NMR (400 MHz, CD_3_OD) δ 8.55 (s, 1H), 8.06–8.01 (m, 2H), 7.78–7.73 (m, 2H), 4.97 (dd, *J =* 14.3 Hz, 2.0 Hz, 1H, H1), 4.59 (dd, *J =* 14.3 Hz, 7.6 Hz, 1H, H1), 3.91–3.88 (m, 1H, H5), 3.78 (dd, *J =* 11.8 Hz, 7.2 Hz, 1H, H7), 3.70 (dd, *J =* 11.8 Hz, 4.9 Hz, 1H, H7), 3.60–3.49 (m, 4H); ^13^C NMR (100 MHz, CD_3_OD) δ 145.8, 125.6, 125.50, 125.46, 123.3, 79.0, 78.7, 74.7, 69.4, 68.4, 61.5, 51.5; ^19^F NMR (376 MHz, CD_3_OD) δ −64.13; HRMS (*m*/*z*): [M + H]^+^ calcd for 390.1279; found, 390.1277; purity by HPLC, UV–vis detection: 99.8%.

**1,2-Dideoxy-1-(4-naphth-1-yl-1*H*-1,2,3-triazol-1-yl)-β-D-galactoheptulose (1l):** Compound **10l** (12 mg, 0.016 mmol) was dissolved in cyclohexene/ethanol 2:1 (4.5 mL), palladium(II) hydroxide on charcoal (10 mg, 20 wt %) was added, and the mixture refluxed overnight at 80 °C. The reaction mixture was diluted with methanol (15 mL), filtered through Celite, evaporated, purified by column chromatography (dichloromethane/methanol 5:1), and then preparative HPLC (gradient from 10% acetonitrile/90% water with 0.1% formic acid to 100% acetonitrile over 20 min, followed by 3 min of 100% acetonitrile) to give **1l** (5 mg, 79%) as a clear solid. [α]_D_^20^ 22 (*c* 0.5, MeOH); ^1^H NMR (400 MHz, CD_3_OD) δ 8.43 (s, 1H), 8.32–8.26 (m, 1H), 7.98–7.93 (m, 2H), 7.73 (d, *J =* 7.1 Hz, 1H), 7.60–7.53 (m, 3H), 5.02 (dd, *J =* 14.4 Hz, 1.2 Hz, 1H, H1), 4.69 (dd, *J =* 14.2 Hz, 7.6 Hz, 1H, H1), 3.91 (dd, *J =* 2.7 Hz,1.0 Hz, 1H, H5), 3.81 (dd, *J =* 11.8 Hz, 7.5 Hz, 1H, H7), 3.71 (dd, *J =* 11.4 Hz, 4.8 Hz, 1H, H7), 3.67–3.61 (m, 1H, HX), 3.59–3.52 (m, 3H); ^13^C NMR (100 MHz, CD_3_OD) δ 133.8, 131.2, 128.6, 128.1, 126.9, 126.3, 125.7, 125.3, 124.98, 124.95, 78.9, 78.6, 74.8, 69.5, 68.3, 61.6, 51.4; HRMS (*m*/*z*): [M + H]^+^ calcd for 372.1563; found, 372.1559; purity by HPLC, UV–vis detection: 96.3%.

**1,2-Dideoxy-1-[4-(4-biphenyl)-1*H*-1,2,3-triazol-1-yl]-β-D-galactoheptulose (1m):** Compound **10m** (69 mg, 0.085 mmol) was dissolved in cyclohexene/ethanol 2:1 (4.5 mL), palladium(II) hydroxide on charcoal (9 mg, 20 wt %) was added, and the mixture refluxed overnight at 80 °C. The reaction mixture was diluted with methanol (15 mL), filtered through Celite, evaporated, purified by column chromatography (dichloromethane/methanol 5:1), and then preparative HPLC (gradient from 10% acetonitrile/90% water with 0.1% formic acid to 100% acetonitrile over 20 min, followed by 3 min of 100% acetonitrile) to give **1m** (3 mg, 10%) as a white powder. [α]_D_^20^ 45 (*c* 0.7, MeOH); ^1^H NMR (400 MHz, CD_3_OD) δ 8.47 (s, 1H), 7.95–7.90 (m, 2H), 7.75–7.65 (m, 4H), 7.50–7.44 (m, 2H), 7.37 (tt, *J =* 7.4 Hz, 1.2 Hz, 1H), 4.97 (dd, *J =* 14.2 Hz, 1.9 Hz, 1H, H1), 4.58 (dd, *J =* 14.0 Hz, 7.5 Hz, 1H, H1), 3.92–3.89 (m, 1H, H5), 3.87–3.68 (m, 2H), 3.62–3.49 (m, 4H); ^13^C NMR (100 MHz, CD_3_OD) δ 147.0, 140.9, 140.4, 128.5, 127.2, 127.1, 126.5, 125.7, 122.3, 78.9, 78.8, 74.7, 69.4, 68.4, 61.5, 51.5; HRMS (*m*/*z*): [M + H]^+^ calcd for 398.1717; found, 398.1716; purity by HPLC, UV–vis detection: 99.7%.

**1,2-Dideoxy-1-(4-naphth-2-yl-1*H*-1,2,3-triazol-1-yl)-β-D-galactoheptulose (1n):** Compound **10n** (96 mg, 0.131 mmol) was dissolved in cyclohexene/ethanol 2:1 (6 mL), palladium(II) hydroxide on charcoal (73 mg, 20 wt %) was added, and the mixture refluxed overnight at 80 °C. The reaction mixture was diluted with methanol (15 mL), filtered through Celite, evaporated, purified by column chromatography (dichloromethane/methanol 5:1), and then preparative HPLC (gradient from 10% acetonitrile/90% water with 0.1% formic acid to 100% acetonitrile over 20 min, followed by 3 min of 100% acetonitrile) to give **1n** (26 mg, 54%) as a clear solid. [α]_D_^20^ 12 (*c* 0.7, MeOH); ^1^H NMR (400 MHz, CD_3_OD) δ 8.54 (s, 1H), 8.32 (s, 1H), 7.97–7.85 (m, 4H), 7.54–7.47 (m, 2H), 4.98 (dd, *J =* 14.2 Hz, 2.0 Hz, 1H, H1), 4.60 (dd, *J =* 14.2 Hz, 7.5 Hz, 1H, H1), 3.91 (dd, *J =* 2.8 Hz, 1.0 Hz, 1H, H5), 3.81 (dd, *J =* 12.0 Hz, 7.3 Hz, 1H, H7), 3.72 (dd, *J =* 12.0 Hz, 4.9 Hz, H7), 3.63–3.51 (m, 4H); ^13^C NMR (100 MHz, CD_3_OD) δ 147.3, 133.6, 133.2, 128.3, 127.80, 127.75, 127.4, 126.2, 125.9, 123.9, 123.4, 122.6, 79.0, 78.8, 74.8, 69.5, 68.4, 61.5, 51.5; HRMS (*m*/*z*): [M + H]^+^ calcd for 372.1557; found, 372.1559; purity by HPLC, UV–vis detection: 99.5%.

**3,4,5,7-Tetra-*O-*benzyl-2-deoxy-β-D-galactoheptulose (3):** Compound **2** (3.66 g, 6.82 mmol) was dissolved in dry tetrahydrofuran (130 mL) under nitrogen and cooled to 0 °C. Borane dimethyl sulfide complex in dry tetrahydrofuran (4.78 mL, 9.55 mmol, 2 M) was added slowly, and the reaction was kept at 0 °C for 2 h. The reaction mixture went from light yellow to colorless during the addition of the borane dimethyl sulfide complex. Upon complete consumption of **2**, distilled water (15 mL) was added to the reaction mixture slowly (dropwise at first) while vigorous gas evolution was observed. After completion of the gas evolution, sodium hydroxide (14 mL, 14.00 mmol, 1 M) and hydrogen peroxide (33%, 2 mL, 20.5 mmol) were added, and the reaction mixture left for 2 h. Upon completion of the reaction, 1 M hydrochloric acid was added to the reaction mixture until the pH was approximately 7. The reaction mixture was poured into brine (150 mL), extracted with dichloromethane (3 × 150 mL) after which the organic phases were pooled, dried with anhydrous sodium sulfate, filtered, and evaporated. The crude was purified with column chromatography (heptane/ethyl acetate 1:1) to give **3** (3.38 g, 89%) as a clear, viscous oil. The diastereoselectivity was determined to be α:β 1:19 by HPLC, using the same protocol as is used for purity determinations. The stereochemistry of the β diastereomer, the only one isolated, was assigned using NOESY. [α]_D_^20^ 31 (*c* 1, CH_2_Cl_2_); ^1^H NMR (400 MHz, CDCl_3_) δ 7.41–7.24 (m, 20H), 4.97 (d, *J =* 9.2 Hz, 1H), 4.94 (d, *J =* 8.4 Hz, 1H), 4.78 (d, *J =* 11.9 Hz, 1H), 4.71 (d, *J =* 11.9 Hz, 1H), 4.67 (d, *J =* 10.9 Hz, 1H), 4.62 (d, *J =* 11.6 Hz, 1H), 4.49 (d, *J =* 11.6 Hz, 1H), 4.43 (d, *J =* 11.9 Hz, 1H), 3.99 (d, *J =* 2.7 Hz, 1H, H5), 3.95 (d, *J =* 9.4 Hz, 1H, H3), 3.87 (dd, *J =* 11.9 Hz, 2.7 Hz, 1H, H1), 3.71 (dd, *J =* 11.7 Hz, 5.2 Hz, 1H, H1), 3.65 (dd, *J =* 9.4 Hz, 2.7 Hz, 1H, H4), 3.62–3.50 (m, 3H), 3.40–3.33 (m, 1H, H2); ^13^C NMR (100 MHz, CDCl_3_) δ 138.7, 138.32, 138.26, 137.8, 128.5, 128.5, 128.3, 128.2, 128.1, 128.0, 127.89, 127.85, 127.72, 127.65, 127.6, 84.7, 79.6, 77.0, 75.4, 75.3, 74.6, 73.8, 73.6, 72.4, 69.0, 62.6; ^1^H NMR (400 MHz, pyridine-*d*_5_) δ 7.60–7.20 (m, 20H), 5.19 (d, *J =* 11.1 Hz, 1H), 5.13 (d, *J =* 11.1 Hz, 1H), 4.94–4.86 (m, 2H), 4.79 (d, *J =* 11.1 Hz, 2H), 4.60–4.48 (m, 3H), 4.43 (t, *J =* 9.1 Hz, 1H, H2), 4.32–4.25 (m, 2H), 4.14 (dd, *J =* 11.7 Hz, 4.6 Hz, 1H, H3), 3.95–3.85 (m, 3H), 3.77 (dd, *J =* 7.6 Hz, 4.3 Hz, 1H, H1), 3.70–3.64 (m, 1H); ^13^C NMR (100 MHz, pyridine-*d*_5_) δ 139.6, 139.5, 139.2, 138.8, 132.53, 132.46, 130.0, 128.57, 128.55, 128.5, 128.4, 128.3, 128.2, 128.11, 128.06, 128.04, 127.99, 127.8, 127.7, 127.6, 127.5, 85.0, 81.7, 77.1, 75.8, 75.1, 75.0, 75.1, 73.3, 72.0, 69.5, 62.1; HRMS (*m*/*z*): [M + Na]^+^ calcd for 577.2526; found, 577.2566.

**3,4,5,7-Tetra-*O-*benzyl-2-deoxy-β-D-mannoheptulose (5):** Compound **4** (35 mg, 0.065 mmol) was dissolved in dry tetrahydrofuran (3 mL) under nitrogen and cooled to 0 °C. Borane dimethyl sulfide complex in dry tetrahydrofuran (40 µL, 0.078 mmol, 2 M) was added slowly and the reaction was kept at 0 °C for 2 h. The reaction mixture went from light yellow to colorless during the addition of the borane dimethyl sulfide complex. Upon complete consumption of the starting material, distilled water (0.66 mL) was added to the reaction mixture slowly (dropwise at first) while vigorous gas evolution was observed. After completion of the gas evolution, sodium hydroxide (131 µL, 0.131 mmol, 1 M) and hydrogen peroxide (33%, 13 µL, 0.131 mmol) were added, and the reaction mixture left for 2 h. Upon completion of the reaction, 1 M hydrochloric acid was added to the reaction mixture until the pH was approximately 7. The reaction mixture was poured into brine (30 mL), extracted with dichloromethane (3 × 30 mL) after which the organic phases were pooled, dried with anhydrous sodium sulfate, filtered and evaporated. The crude was purified with column chromatography (heptane/ethyl acetate 1:1) to give **5** (28 mg, 78%) as a clear, viscous oil. The diastereoselectivity was determined to be α:β 1:99 by HPLC, using the same protocol as is used for purity determinations. The stereochemistry of the β diastereomer, the only one isolated, was assigned using NOESY. [α]_D_^20^ −37 (*c* 0.1, CH_3_CN); ^1^H NMR (400 MHz, CDCl_3_) δ 7.43–7.26 (m, 18H), 7.22–7.18 (m, 2H), 5.00 (d, *J =* 12.0 Hz, 1H), 4.91 (d, 10.7 Hz, 1H), 4.83–4.74 (m, 2H), 4.72–4.54 (m, 4H), 3.95 (t, *J =* 9.8 Hz, 1H, H5), 3.90 (d, 2.6 Hz, 1H, H3), 3.87–3.70 (m, 3H), 3.65 (dd, *J =* 9.4 Hz, 2.7 Hz, 1H, H6), 3.54–3.47 (m, 2H), 3.44 (t, *J =* 5.7 Hz, 1H, H2); ^13^C NMR (100 MHz, CDCl_3_) δ 138.4, 138.2, 128.53, 128.45, 128.37, 128.35, 128.1, 127.94, 127.89, 127.8, 127.7, 127.62, 127.59, 85.0, 79.6, 78.6, 75.4, 75.3, 74.1, 73.5, 73.3, 72.7, 69.6, 62.5; ^1^H NMR (400 MHz, pyridine-*d*_5_) δ 7.43–7.22 (m, 20H), 5.23 (d, *J =* 11.2 Hz, 1H), 5.10 (d, *J =* 11.2 Hz, 1H), 4.93–4.87 (partially obscured by solvent), 4.80–4.64 (m, 3H), 4.56 (d, *J =* 4.56 Hz, 1H), 4.48 (d, *J =* 2.9 Hz, 1H, H3), 4.32 (t, *J =* 9,5 Hz, 1H, H4), 4.27 (m, 1H, H1), 4.18 (m, 1H, H1), 3.95–3.88 (m, 4H), 3.76 (dt, *J =* 9.5 Hz, 3.4 Hz, 1H, H5); ^13^C NMR (100 MHz, pyridine-*d*_5_) δ 141.3, 140.9, 140.7, 140.6, 130.1, 129.90, 129.86, 129.49, 129.45, 129.2, 129.1, 128.99, 128.95, 86.7, 81.4, 81.3, 77.4, 76.5, 76.4, 74.8, 73.3, 71.8, 62.9; HRMS (*m*/*z*): [M + H]^+^ calcd for 555.2743; found, 555.2747.

**3,4,5,7-Tetra-*O-*benzyl-2-deoxy-β-D-glucoheptulose (7):** Compound **6** (67 mg, 0.125 mmol) was dissolved in dry tetrahydrofuran (3 mL) under nitrogen and cooled to 0 °C. Borane dimethyl sulfide complex in dry tetrahydrofuran (75 µL, 0.150 mmol, 2 M) was added slowly and the reaction was kept at 0 °C for 2 h. The reaction mixture went from light yellow to colorless during the addition of the borane dimethyl sulfide complex. Upon complete consumption of the starting material, distilled water (0.66 mL) was added to the reaction mixture slowly (dropwise at first) while vigorous gas evolution was observed. After completion of the gas evolution, sodium hydroxide (250 µL, 0.251 mmol, 1 M) and hydrogen peroxide (33%, 25 µL, 0.251 mmol) were added, and the reaction mixture left for 2 h. Upon completion of the reaction, 1 M hydrochloric acid was added to the reaction mixture until the pH was approximately 7. The reaction mixture was poured into brine (30 mL), extracted with dichloromethane (3 × 30 mL) after which the organic phases were pooled, dried with anhydrous sodium sulfate, filtered and evaporated. The crude was purified with column chromatography (heptane/ethyl acetate 1:1) to give **7** (31 mg, 45%) as a diastereomeric mixture. Three consecutive preparative HPLC purifications gave 3 mg of pure α-**7**, 17 mg of pure β-**7**, and 11 mg of **7** α/β-mixture as clear and viscous oils. The diastereoselectivity was determined to be α:β 1:2.3 by HPLC using the same protocol as is used for purity determinations. The stereochemistry was assigned using NOESY. Data for α-**7**: [α]_D_^20^ 52 (*c* 0.2, CH_3_CN); ^1^H NMR (400 MHz, acetone-*d*_6_) δ 7.44–7.24 (m, 20H), 4.93 (d, *J =* 11.5 Hz, 1H), 4.84 (d, *J =* 11.0 Hz, 1H), 4.81 (d, *J =* 11.3 Hz, 1H), 4.76 (m, 2H), 4.66–4.53 (m, 3H), 4.18–4.12 (m, 1H), 4.08–4.00 (m, 1H), 3.93–3.69 (m, 5H), 3.64–3.56 (m, 2H); ^13^C NMR (100 MHz, acetone-*d*_6_) δ 139.3, 139.0, 138.9, 138.8, 128.3, 128.2, 128.13, 128.11, 127.8, 127.7, 127.6, 127.5, 127.32, 127.29, 127.2, 82.4, 79.5, 78.3, 74.6, 74.6, 74.3, 72.9, 72.6, 72.4, 69.6, 57.9, 57.8; ^1^H NMR (400 MHz, pyridine-*d*_5_) δ 7.51–7.23 (m, 20H), 5.09–5.01 (m, 2H), 5.00–4.91 (m, 2H, obstructed by solvent), 4.81–4.75 (m, 3H), 4.69–4.53 (m, 3H), 4.48 (t, *J =* 8.1 Hz, 1H, H3), 4.37 (ddd, *J =* 9.9 Hz, 4.0 Hz, 1.9 Hz, 1H, H6), 4.34–4.27 (m, 2H), 4.04 (dd, *J =* 8.9 Hz, 5.9 Hz, 1H, H2), 3.98–3.88 (m, 2H), 3.85 (dd, *J =* 10.8 Hz,2.2 Hz, 1H, H7); ^13^C NMR (100 MHz, pyridine-*d*_5_) δ 141.1, 140.8, 140.6, 140.4, 130.01, 129.99, 129.97, 129.95, 129.52, 129.49, 129.45, 129.40, 129.3, 129.12, 129.10, 84.4, 81.6, 80.3, 77.1, 76.3, 76.1, 74.8, 74.7, 74.5, 71.5, 60.2; HRMS (*m*/*z*): [M + H]^+^ calcd for 555.2755; found, 555.2747. Data for β-**7**: [α]_D_^20^ 20 (*c* 1.1, CH_3_CN); ^1^H NMR (400 MHz, acetone-*d*_6_) δ 7.43–7.24 (m, 20H), 4.93 (s, 2H), 4.89 (d, *J =* 7.8 Hz, 1H), 4.86 (d, *J =* 7.6 Hz, 1H), 4.75 (d, *J =* 11.1 Hz, 1H), 4.67 (d, *J =* 10.9 Hz, 1H), 3.87 (m, 1H), 3.80–3.68 (m, 4H), 3.60 (td, *J =* 9.3 Hz, 2.2 Hz, 2H), 3.51 (ddd, *J =* 9.7 Hz, 3.8 Hz, 2.3 Hz, 1H), 3.36 (ddd, *J =* 9.7 Hz, 4.2 Hz, 2.2 Hz, 1H); ^13^C NMR (100 MHz, acetone-*d*_6_) δ 139.2, 139.0, 138.9, 138.8, 128.20, 128.17, 128.15, 127.73, 127.71, 127.69, 127.5, 127.4, 127.34, 127.26, 87.1, 80.1, 78.9, 78.6, 78.4, 74.9, 74.43, 74.36, 73.0, 69.4, 61.4, 61.3; ^1^H NMR (400 MHz, pyridine-*d*_5_) δ 7.52–7.41 (m, 6H), 7.40–7.23 (m, 14H), 5.10–4.94 (m, partially solvent obstructed), 4.77 (d, *J =* 11.2 Hz, 1H), 4.63 (d, *J =* 11.9 Hz, 1H), 4.55 (d, *J =* 12.0 Hz, 1H), 4.27 (d, *J =* 12.1 Hz, 1H, H1), 4.11 (dd, *J =* 11.6 Hz, 4.1 Hz, 1H, H1), 4.04–3.85 (m, 5H), 3.73 (dt, *J =* 9.4 Hz, 2.9 Hz, 1H, H6), 3.65 (m, 1H, H2); ^13^C NMR (100 MHz, pyridine-*d*_5_) δ 139.5, 139.3, 139.1, 138.8, 128.51, 128.48, 128.47, 127.97, 127.95, 127.8, 127.7, 127.62, 127.55, 87.4, 81.2, 79.1, 78.81, 78.75, 75.2, 74.7, 73.3, 69.9, 61.5; HRMS (*m*/*z*): [M + H]^+^ calcd for 555.2745; found, 555.2747.

**3,4,5,7-Tetra-O-benzyl-2-deoxy-1-methanesulfonyl-β-D-galactoheptulose (8):** Compound **3** (3.38 g, 6.08 mmol) was dissolved in dry pyridine (16.5 mL) under nitrogen and cooled to 0 °C. Methanesulfonyl chloride (1.55 mL, 20.10 mmol) was added slowly. The reaction mixture turns yellow/orange upon addition. The reaction was left for 2 h, after which the reaction mixture was poured into brine (200 mL with 20 mL 5% hydrochloric acid), extracted with dichloromethane (3 × 200 mL) after which the organic phases were pooled. The organic phases were washed once with brine, dried with anhydrous sodium sulfate, filtered and evaporated. The crude was purified with column chromatography (heptane/ethyl acetate 1:1) to give **8** (3.51 g, 91%) as a cream white solid. [α]_D_^20^ 7 (*c* 1.1, CH_2_Cl_2_); ^1^H NMR (400 MHz, CDCl_3_) δ 7.52–7.25 (m, 20H), 5.06 (d, *J =* 4.0 Hz, 1H), 5.04 (d, *J =* 4.6 Hz, 1H), 4.88 (d, *J =* 11.6 Hz, 1H), 4.80 (d, *J =* 12.2 Hz, 1H), 4.75 (d, *J =* 10.7 Hz, 1H), 4.67–4.61 (m, 2H), 4.58–4.52 (m, 2H), 4.47 (dd, *J*^1^ = 12.2 Hz, 4.9 Hz, H1), 4.09 (d, *J =* 2.7 Hz, 1H, H5), 4.03 (t, *J =* 9.7 Hz, 1H, H3), 3.73 (dd, *J*^1^ = 9.1, 2.7 Hz, 1H, H4), 3.71–3.54 (m, 4H), 2.99 (s, 3H); ^13^C NMR (100 MHz, CDCl_3_) δ 138.5, 138.1, 138.0, 137.8, 128.63, 128.60, 128.4, 128.3, 128.16, 128.04, 128.02, 127.92, 127.86, 127.7, 84.5, 77.7, 77.2, 75.5, 74.8, 74.2, 73.7, 73.6, 72.3, 69.9, 68.8, 38.0; HRMS (*m*/*z*): [M + Na]^+^ calcd for 655.2343; found, 655.2342.

**1-Azido-3,4,5,7-tetra-*O-*benzyl-1,2-dideoxy-β-D-galactoheptulose (9):** Compound **8** (3.51 g, 5.55 mmol) was dissolved in dry DMF (40 mL) and sodium azide (791 mg, 12.17 mmol) was added. The reaction mixture was heated to 95 °C and left overnight. Upon completion, the reaction mixture was poured into distilled water (500 mL) and washed with dichloromethane (3 × 150 mL). The organic phases were pooled and washed with brine (450 mL), dried with anhydrous sodium sulfate, and evaporated. The crude was purified with column chromatography (heptane/ethyl acetate 1:1) to give **9** (2.90 g, 90%) as a clear, viscous oil that after about two weeks crystallized into a grey-white sticky solid. [α]_D_^20^ −5 (*c* 1.1, CH_2_Cl_2_); IR ν: 2101 cm^−1^, azide; ^1^H NMR (400 MHz, CDCl_3_) δ 7.45–7.25 (m, 20H), 5.00 (d, *J =* 3.3 Hz, 1H), 4.97 (d, *J =* 2.5 Hz, 1H), 4.80 (d, *J =* 11.7 Hz, 1H), 4.71 (d, *J =* 12.0 Hz, 1H), 4.68–4.61 (m, 2H), 4.52 (d, *J =* 11.7 Hz, 1H), 4.56 (d, *J =* 11.7 Hz, 1H), 4.04 (d, *J =* 2.6 Hz, 1H, H5), 3.90 (t, *J =* 9.4 Hz, 1H, H3), 3.67–3.58 (m, 4H), 3.53–3.46 (m, 2H), 3.39 (dd, *J =* 12.4 Hz, 7.2 Hz, 1H, H1); ^13^C NMR (100 MHz, CDCl_3_) δ 138.7, 138.2, 138.1, 137.9, 128.50, 128.46, 128.3, 128.2, 127.97, 127.96, 127.9, 127.8, 127.8, 127.6, 84.6, 79.1, 77.2, 75.7, 75.4, 74.5, 73.62, 73.55, 72.2, 69.0, 51.5; HRMS (*m*/*z*): [M + Na]^+^ calcd for 602.2634; found, 602.2631.

**3,4,5,7-Tetra-*O-*benzyl-1,2-dideoxy-1-[4-phenyl-1*H*-1,2,3-triazol-1-yl]-β-D-galactoheptulose (10a):** Compound **9** (100 mg, 0.184 mmol) and copper(I) iodide (4 mg, 0.035 mmol) were dissolved in dry acetonitrile (3 mL) under nitrogen, triethylamine (52 μL, 0.368 mmol) and ethynylbenzene (22 μL, 0.194 mmol) were added, and the reaction was left at 55 °C overnight. Upon completion, the reaction mixture was poured into ethyl acetate (20 mL) and washed with brine (20 mL). The brine was extracted with ethyl acetate (2 × 20 mL), the organic phases pooled, dried with anhydrous sodium sulfate, and evaporated. The crude product was purified with column chromatography (heptane/ethyl acetate 2:1) to give **10a** (96.8 mg, 77%) as a viscous clear oil. [α]_D_^20^ −10 (*c* 1.9, CH_2_Cl_2_); ^1^H NMR (400 MHz, CDCl_3_) δ 8.01 (s, 1H), 7.81–7.75 (m, 2H), 7.45–7.14 (m, 23H), 4.97 (d, *J =* 7.4 Hz, 1H), 4.94 (d, *J =* 6.2 Hz, 1H), 4.81 (d, *J =* 11.7 Hz, 1H), 4.76–4.68 (m, 4H), 4.57 (d, *J =* 11.7 Hz, 1H), 4.51–4.48 (m, 2H), 4.00 (s, 1H, H5), 3.75–3.55 (m, 6H); ^13^C NMR (100 MHz, CDCl_3_) δ 138.5, 138.01, 137.95, 137.8, 132.80, 132.2, 131.0, 129.2, 128.8, 128.7, 128.6, 128.54, 128.50, 128.4, 128.3, 128.0, 127.91, 127.87, 127.85, 127.7, 127.6, 127.54, 127.52, 125.8, 121.4, 84.5, 77.7, 77.2, 75.4, 74.8, 74.6, 73.8, 73.6, 72.2, 69.1, 51.1; HRMS (*m*/*z*): [M + H]^+^ calcd for 682.3288; found, 682.3281.

**3,4,5,7-Tetra-*O-*benzyl-1,2-dideoxy-1-[4-(4-fluorophenyl)-1*H*-1,2,3-triazol-1-yl]-β-D-galactoheptulose (10b):** Compound **9** (113 mg, 0.198 mmol), 1-ethynyl-4-fluorobenzene (29 mg, 0.238 mmol), and copper(I) iodide (4 mg, 0.035 mmol) were dissolved in dry acetonitrile (3 mL) under nitrogen, triethylamine (55 μL,0.397 mmol) was added, and the reaction left at room temperature overnight. Upon completion, the reaction mixture was poured into ethyl acetate (20 mL) and washed with brine (20 mL). The brine was extracted with ethyl acetate (2 × 20 mL), the organic phases pooled, dried with anhydrous sodium sulfate, and evaporated. The crude product was purified with column chromatography (heptane/ethyl acetate 3:1) to give **10b** (97 mg, 70%) as a viscous lightly yellow oil. [α]_D_^20^ −13 (*c* 0.7, CH_2_Cl_2_); ^1^H NMR (400 MHz, CDCl_3_) δ 7.95 (s, 1H), 7.73 (m, 2H), 7.44–7.15 (m, 20H), 7.10–7.03 (m, 2H), 4.96 (d, *J =* 9.2 Hz, 1H), 4.94 (d, *J =* 8.2 Hz, 1H), 4.81 (d, *J =* 11.6 Hz, 1H), 4.76–4.68 (m, 4H), 4.56 (d, *J =* 11.4 Hz, 1H), 4.52–4.49 (m, 2H), 4.00 (d, *J =* 1.8 Hz, 1H, H5), 3.74–3.55 (m, 6H); ^13^C NMR (100 MHz, CDCl_3_) δ 163.8, 161.4, 147.0, 138.6, 138.1, 138.0, 137.8, 128.7, 128.62, 128.60, 128.58, 128.4, 128.1, 128.02, 127.95, 127.9, 127.69, 127.67, 127.6, 127.5, 127.24, 127.21, 84.6, 77.7, 77.3, 75.5, 74.7, 73.9, 73.7, 72.4, 69.2, 51.2; ^19^F NMR (376 MHz, CD_3_OD) δ −114.11; HRMS (*m*/*z*): [M + H]^+^ calcd for 700.3195; found, 700.3187.

**3,4,5,7-Tetra-*O-*benzyl-1,2-dideoxy-1-[4-(3-fluorophenyl)-1*H*-1,2,3-triazol-1-yl]-β-D-galactoheptulose (10c):** Compound **9** (107 mg, 0.185 mmol) and copper(I) iodide (4 mg, 0.035 mmol) were dissolved in dry acetonitrile (3 mL) under nitrogen, triethylamine (51 μL (0.369 mmol) and 1-ethynyl-3-fluorobenzene (26 μL, 0.221 mmol) were added, and the reaction was left at room temperature overnight. Upon completion, the reaction mixture was poured into ethyl acetate (20 mL) and washed with brine (20 mL). The brine was extracted with ethyl acetate (2 × 20 mL), the organic phases pooled, dried with anhydrous sodium sulfate, and evaporated. The crude product was purified with column chromatography (heptane/ethyl acetate 2:1) to give **10c** (81 mg, 63%) as a viscous clear oil. [α]_D_^20^ −10 (*c* 1.0, CH_2_Cl_2_); ^1^H NMR (400 MHz, CDCl_3_) δ 17.98 (s, 1H), 7.53–7.46 (m, 2H), 7.43–7.14 (m, 21H), 7.06–6.99 (m, 1H), 4.95 (d, *J =* 1.9 Hz, 1H), 4.92 (s, 1H), 4.80 (d, *J =* 11.6 Hz, 1H), 4.73 (d, *J =* 2.3 Hz, 1H), 4.71–4.67 (m, 3H), 4.54 (d, *J =* 11.6 Hz, 1H), 4.50–4.47 (m, 2H), 3.99 (d, *J =* 1.8 Hz, 1H, H5), 3.73–3.52 (m, 6H); ^13^C NMR (100 MHz, CDCl_3_) δ 164.5, 162.1, 147.0, 138.4, 138.0, 137.9, 137.7, 130.4, 130.3, 128.54, 128.53, 128.49, 128.46, 128.2, 128.0, 127.93, 127.87, 127.85, 127.58, 127.55, 121,8, 121.3, 114.8, 114.5, 112.7, 112.5, 84.5, 77.6, 77.2, 75.4, 74.8, 74.6, 73.7, 73.6, 72.2, 69.0, 51.1; ^19^F NMR (376 MHz, CD_3_OD) δ −112.85; HRMS (*m*/*z*): [M + H]^+^ calcd for 700.3185; found, 700.3187.

**3,4,5,7-Tetra-*O-*benzyl-1,2-dideoxy-1-[4-(2-fluorophenyl)-1*H*-1,2,3-triazol-1-yl]-β-D-galactoheptulose (10d):** Compound **9** (102 mg, 0.179 mmol) and copper(I) iodide (4 mg, 0.035 mmol) were dissolved in dry acetonitrile (3 mL) under nitrogen, triethylamine (49 μL, 0.352 mmol) and 1-ethynyl-2-fluorobenzene (24 μL, 0.211 mmol) were added, and the reaction was left at room temperature overnight. Upon completion, the reaction mixture was poured into ethyl acetate (20 mL) and washed with brine (20 mL). The brine was extracted with ethyl acetate (2 × 20 mL), the organic phases pooled, dried with anhydrous sodium sulfate, and evaporated. The crude product was purified with column chromatography (heptane/ethyl acetate 2:1) to give **10d** (78 mg, 63%) as a viscous lightly yellow oil. [α]_D_^20^ −10 (*c* 0.8, CH_2_Cl_2_); ^1^H NMR (400 MHz, CDCl_3_) δ 8.32 (td, *J*^1^ = 7.6 Hz, 2.0 Hz, 1H), 8.18 (d, *J =* 3.9 Hz, 1H), 7.45–7.09 (m, 22H), 4.96 (d, *J =* 10.5 Hz, 1H), 4.93 (d, *J =* 11.7 Hz, 1H), 4.81–4.59 (m, 6H), 4.57 (d, *J =* 11.5 Hz, 1H), 4.49 (d, *J =* 11.7 Hz, 1H), 4.43 (d, *J =* 11.7 Hz, 1H), 3.98 (d, *J =* 0.8 Hz, 1H, H5), 3.74–3.65 (m, 3H), 3.65–3.52 (m, 3H); ^13^C NMR (100 MHz, CDCl_3_) δ 158.0, 141.1, 138.5, 138.02, 137.96, 137.8, 129.1, 129.0, 128.53, 128.46, 128.4, 128.3, 128.2, 128.0, 127.9, 127.8, 127.7, 127.6, 127.4, 124.8, 124.6, 124.5, 115.8, 115.6, 84.5, 77.7, 77.2, 75.4, 74.9, 74.5, 73.6, 73.5, 73.2, 72.2, 69.0, 51.1; ^19^F NMR (376 MHz, CD_3_OD) δ −114.53; HRMS (*m*/*z*): [M + H]^+^ calcd for 700.3186; found, 700.3187.

**3,4,5,7-Tetra-*O-*benzyl-1,2-dideoxy-1-[4-(3,4-difluorophenyl)-1*H*-1,2,3-triazol-1-yl]-β-D-galactoheptulose (10e):** Compound **9** (103 mg, 0.176 mmol) and copper(I) iodide (4 mg, 0.035 mmol) were dissolved in dry acetonitrile (3 mL) under nitrogen, triethylamine (49 μL, 0.352 mmol) and 1-ethynyl-3,4-difluorobenzene (25 μL, 0.211 mmol) were added, and the reaction was left at room temperature overnight. Upon completion, the reaction mixture was poured into ethyl acetate (20 mL) and washed with brine (20 mL). The brine was extracted with ethyl acetate (2 × 20 mL), the organic phases pooled, dried with anhydrous sodium sulfate, and evaporated. The crude product was purified with column chromatography (heptane/ethyl acetate 2:1) to give **10e** (86 mg, 68%) as a viscous lightly yellow oil. [α]_D_^20^ −9 (*c* 1.0, CH_2_Cl_2_); ^1^H NMR (400 MHz, CDCl_3_) δ 7.93 (s, 1H), 7.59–7.52 (m, 1H), 7.46–7.02 (m, 22H), 4.95 (d, *J =* 2.9 Hz, 1H), 4.92 (d, *J =* 2.1 Hz, 1H), 4.80 (d, *J =* 11.6 Hz, 1H), 4.75–4.66 (m, 4H), 4.54 (d, *J =* 11.6 Hz, 1H), 4.51–4.47 (m, 2H), 3.99 (d, *J =* 2.0 Hz, 1H, H5), 3.73–3.53 (m, 6H); ^13^C NMR (100 MHz, CDCl_3_) δ 138.4, 137.93, 137.86, 137.7, 128.6, 128.54, 128.50, 128.46, 128.2, 127.99, 127.96, 127.9, 127.6, 121.7, 121.5, 117.7, 117.5, 114.8, 114.6, 84.5, 77.5, 77.2, 75.4, 74.7, 74.6, 73.7, 73.6, 72.3, 69.1, 51.2; ^19^F NMR (376 MHz, CD_3_OD) δ −137.34, −137.40, −138.89, −138.95; HRMS (*m*/*z*): [M + H]^+^ calcd for 718.3102; found, 718.3093.

**3,4,5,7-Tetra-*O-*benzyl-1,2-dideoxy-1-[4-(3,5-difluorophenyl)-1*H*-1,2,3-triazol-1-yl]-β-D-galactoheptulose (10f):** Compound **9** (106 mg, 0.183 mmol) and copper(I) iodide (4 mg, 0.035 mmol) were dissolved in dry acetonitrile (3 mL) under nitrogen, triethylamine (51 μL, 0.366 mmol) and 1-ethynyl-3,5-difluorobenzene (26 μL, 0.220 mmol) was added, and the reaction was left at room temperature overnight. Upon completion, the reaction mixture was poured into ethyl acetate (20 mL) and washed with brine (20 mL). The brine was extracted with ethyl acetate (2 × 20 mL), the organic phases pooled, dried with anhydrous sodium sulfate, and evaporated. The crude product was purified with column chromatography (heptane/ethyl acetate 2:1) to give **10f** (88 mg, 67%) as a viscous lightly yellow oil. [α]_D_^20^ −12 (*c* 1.1, CH_2_Cl_2_); ^1^H NMR (400 MHz, CDCl_3_) δ 7.96 (s, 1H), 7.44–7.17 (m, 22H), 6.78 (tt, *J =* 8.9 Hz, 2.3 Hz, 1H), 4.95 (d, *J =* 4.0 Hz, 1H), 4.92 (d, *J =* 4.8 Hz, 1H), 4.80 (d, *J =* 11.8 Hz, 1H), 4.75–4.66 (m, 4H), 4.53 (d, *J =* 11.6 Hz, 1H), 4.51–4.48 (m, 2H), 3.98 (d, *J =* 2.1 Hz, 1H, H5), 3.72–3.52 (m, 6H); ^13^C NMR (100 MHz, CDCl_3_) δ 164.6, 164.5, 162.2, 162.0, 138.3, 137.9, 137.9, 137.6, 128.56, 128.55, 128.50, 128.46, 128.2, 128.0, 127.91, 127.87, 127.60, 127.58, 127.57, 122.2, 108.6, 108.3, 103.3, 103.0, 102.8, 84.5, 77.5, 77.2, 75.4, 74.7, 74.6, 73.7, 73.6, 72.2, 69.0, 51.2; ^19^F NMR (376 MHz, CD_3_OD) δ −109.45; HRMS (*m*/*z*): [M + H]^+^ calcd for 718.3097; found, 718.3093.

**3,4,5,7-Tetra-*O-*benzyl-1,2-dideoxy-1-[4-(2,4-difluorophenyl)-1*H*-1,2,3-triazol-1-yl]-β-D-galactoheptulose (10g):** Compound **9** (150 mg, 0.259 mmol), 1-ethynyl-2,4-difluorobenzene (40 mg, 0.285 mmol), and copper(I) iodide (10 mg, 0.052 mmol) were dissolved in dry acetonitrile (4 mL) under nitrogen, triethylamine (72 μL, 0.518 mmol) was added, and the reaction left at 60 °C overnight. Upon completion, the reaction mixture was poured into ethyl acetate (20 mL) and washed with brine (20 mL). The brine was extracted with ethyl acetate (2 × 20 mL), the organic phases pooled, dried with anhydrous sodium sulfate, and evaporated. The crude product was purified with column chromatography (heptane/ethyl acetate 3:1) to give **10g** (136 mg, 73%) as a sticky clear solid. [α]_D_^20^ −10 (*c* 1.6, CH_3_CN); ^1^H NMR (400 MHz, CDCl_3_) δ 8.31–8.25 (m, 2H), 8.11 (d, *J =* 4.0 Hz, 1H), 7.45–7.17 (m, 20H), 7.01 (td, *J =* 8.6 Hz, 2.3 Hz, 1H), 6.89–6.83 (m, 1H), 4.98–4.90 (m, 2H), 4.81–4.69 (m, 4H), 4.68–4.60 (m, 1H), 4.56 (d, *J =* 11.3 Hz, 1H), 4.51–4.42 (m, 2H), 3.98 (s, 1H, H5), 3.72–3.54 (m, 6H); ^13^C NMR (100 MHz, CDCl_3_) δ 163.3, 163.2, 160.1, 160.0, 158.1, 158.0, 140.3, 138.4, 137.91, 137.85, 137.7, 128.73, 128.66, 128.62, 128.56, 128.44, 128.35, 128.3, 128.2, 128.1, 127.9, 127.7, 127.6, 127.52, 127.49, 127.4, 124.2, 124.1, 115.3, 111.9, 111.8, 111.7, 104.2, 103.9, 103.7, 84.4, 77.5, 77.1, 75.3, 74.8, 74.4, 73.51, 73.47, 72.1, 69.0, 51.1; ^19^F NMR (376 MHz, CD_3_OD) δ −110.77, −110.96; HRMS (*m*/*z*): [M + H]^+^ calcd for 718.3091; found, 718.9093.

**3,4,5,7-Tetra-*O-*benzyl-1,2-dideoxy-1-[4-(4-methylphenyl)-1*H*-1,2,3-triazol-1-yl]-β-D-galactoheptulose (10h):** Compound **9** (103 mg, 0.178 mmol) and copper(I) iodide (4 mg, 0.035 mmol) were dissolved in dry acetonitrile (3 mL) under nitrogen, triethylamine (50 μL, 0.356 mmol) and 4-ethynyltoluene (27 μL, 0.214 mmol) were added, and the reaction was left at 50 °C overnight. Upon completion, the reaction mixture was poured into ethyl acetate (20 mL) and washed with brine (20 mL). The brine was extracted with ethyl acetate (2 × 20 mL), the organic phases pooled, dried with anhydrous sodium sulfate, and evaporated. The crude product was purified with column chromatography (heptane/ethyl acetate 2:1) to give **10h** (79 mg, 64%) as viscous clear oil. [α]_D_^20^ −12 (*c* 0.7, CH_2_Cl_2_); ^1^H NMR (400 MHz, CDCl_3_) δ 7.95 (s, 1H), 7.69–7.64 (m, 2H), 7.44–7.14 (m, 22H), 4.96 (d, *J =* 5.1 Hz, 1H), 4.93 (d, *J =* 4.3 Hz, 1H), 4.79 (d, *J =* 11.6 Hz, 1H), 4.75–4.61 (m, 4H), 4.56 (d, *J =* 11.6 Hz, 1H), 4.50–4.45 (m, 2H), 3.99 (s, 1H, H5), 3.75–3.52 (m, 6H), 2.41 (s, 3H); ^13^C NMR (100 MHz, CDCl_3_) δ 138.5, 138.0, 137.9, 137.7, 137.6, 129.4, 128.54, 128.52, 128.49, 128.3, 128.1, 128.0, 127.9, 127.8, 127.6, 127.5, 125.7, 121.0, 84.5, 77.8, 77.2, 75.4 74.9, 74.6, 73.7, 73.5, 72.2, 69.0, 51.1, 21.3; HRMS (*m*/*z*): [M + H]^+^ calcd for 696.3450; found, 696.3437.

**3,4,5,7-Tetra-*O-*benzyl-1,2-dideoxy-1-[4-(3-methylphenyl)-1*H*-1,2,3-triazol-1-yl]-β-D-galactoheptulose (10i):** Compound **9** (106 mg, 0.183 mmol) and copper(I) iodide (4 mg, 0.035 mmol) were dissolved in dry acetonitrile (3 mL) under nitrogen, triethylamine (50 μL, 0.366 mmol) and 3-ethynyltoluene (27 μL, 0.214 mmol) were added, and the reaction was left at 50 °C overnight. Upon completion, the reaction mixture was poured into ethyl acetate (20 mL) and washed with brine (20 mL). The brine was extracted with ethyl acetate (2 × 20 mL), the organic phases pooled, dried with anhydrous sodium sulfate, and evaporated. The crude product was purified with column chromatography (heptane/ethyl acetate 2:1) to give **10i** (83 mg, 65%) as a viscous lightly yellow oil. [α]_D_^20^ −7.8 (*c* 0.8, CH_2_Cl_2_); ^1^H NMR (400 MHz, CDCl_3_) δ 7.97 (s, 1H), 7.69 (s, 1H), 7.52 (d, *J =* 7.63 Hz, 1H), 7.44–7.11 (m, 22H), 4.96 (d, *J =* 3.2 Hz, 1H), 4.93 (d, *J =* 1.6 Hz, 1H), 4.79 (d, *J =* 11.7 Hz, 1H), 4.75–4.59 (m, 4H), 4.56 (d, *J =* 11.7 Hz, 1H), 4.50–4.45 (m, 2H), 3.98 (d, *J =* 1.1 Hz, 1H, H5), 3.76–3.52 (m, 6H), 2.40 (s, 3H); ^13^C NMR (100 MHz, CDCl_3_) δ 147.7, 138.5, 138.4, 138.0, 137.9, 137.7, 130.7, 128.7, 128.54, 128.52, 128.48, 128.0, 127.9, 127.8, 127.57, 127.55, 127.5, 126.5, 122.9, 121.4, 84.5, 77.7, 77.2, 75.4, 74.9, 74.5, 73.7, 73.5, 72.2, 69.0, 51.1, 21.5; HRMS (*m*/*z*): [M + H]^+^ calcd for 696.3442; found, 696.3437.

**3,4,5,7-Tetra-*O-*benzyl-1,2-dideoxy-1-[4-(4-trifluoromethoxyphenyl)-1*H*-1,2,3-triazol-1-yl]-β-D-galactoheptulose (10j):** Compound **9** (100 mg, 0.173 mmol), 4-(trifluoromethoxy)ethynylbenzene (34 mg, 0.181 mmol), and copper(I) iodide (7 mg, 0.035 mmol) were dissolved in dry acetonitrile (3 mL) under nitrogen, (50 μL, 0.356 mmol) triethylamine was added, and the reaction left at room temperature overnight. Upon completion, the reaction mixture was poured into ethyl acetate (20 mL) and washed with brine (20 mL). The brine was extracted with ethyl acetate (2 × 20 mL), the organic phases pooled, dried with anhydrous sodium sulfate, and evaporated. The crude product was purified with column chromatography (heptane/ethyl acetate 2:1) to give **10j** (103 mg, 78%) as a clear solid. [α]_D_^20^ −19 (*c=* 0.8, CHCl_3_); ^1^H NMR (400 MHz, CDCl_3_) δ 8.00 (s, 1H), 7.76–7.71 (m, 2H), 7.44–7.15 (m, 22H), 4.98–4.91 (m, 2H), 4.81 (d, *J =* 11.8 Hz, 1H), 4.77–4.66 (m, 4H), 4.55 (d, *J =* 11.8 Hz, 1H), 4.52–4.49 (m, 2H), 4.00 (d, *J =* 2.3 Hz, 1H, H5), 3.75–3.55 (m, 6H); ^13^C NMR (100 MHz, CDCl_3_) δ 148.7, 146.5, 138.4, 138.0, 137.9, 137.7, 129.7, 128.56, 128.54, 128.51, 128.48, 128.4, 128.2, 128.0, 127.93, 127.87, 127.8, 127.59, 127.55, 127.5, 127.1, 121.8, 121.6, 121.4, 84.5, 77.6, 77.2, 75.4, 74.7, 74.6, 73.8, 73.6, 72.3, 69.2, 51.1; ^19^F NMR (376 MHz, CD_3_OD) δ −57.78; HRMS (*m*/*z*): [M + H]^+^ calcd for 766.3108; found, 766.3104.

**3,4,5,7-Tetra-*O-*benzyl-1,2-dideoxy-1-[4-(4-trifluoromethylphenyl]-*1H*-1,2,3-triazol-1-yl)-β-D-galactoheptulose (10k):** Compound **9** (100 mg, 0.178 mmol), 4-(trifluoromethane)ethynylbenzene (31 mg, 0.181 mmol), and copper(I) iodide (7 mg, 0.035 mmol) were dissolved in dry acetonitrile (3 mL) under nitrogen, triethylamine (50 μL, 0.356 mmol) was added, and the reaction left at room temperature overnight. Upon completion, the reaction mixture was poured into ethyl acetate (20 mL) and washed with brine (20 mL). The brine was extracted with ethyl acetate (2 × 20 mL), the organic phases pooled, dried with anhydrous sodium sulfate, and evaporated. The crude product was purified with column chromatography (heptane/ethyl acetate 2:1) to give **10k** (94 mg, 73%) as a white solid. [α]_D_^20^ −22 (*c* 1.0, CHCl_3_); ^1^H NMR (400 MHz, CDCl_3_) δ 8.08 (s, 1H), 7.85–7.80 (m, 2H), 7.65–7.60 (m, 2H), 7.46–7.15 (m, 20H), 4.98 (d, *J =* 7.1 Hz, 1H), 4.95 (d, *J =* 6.1 Hz, 1H), 4.82 (d, *J =* 11.6 Hz, 1H), 4.77–4.69 (m, 4H), 4.56 (d, *J =* 11.4 Hz, 1H), 4.54–4.51 (m, 2H), 4.02 (d, *J =* 2.4 Hz, 1H, H5), 3.78–3.56 (m, 6H); ^13^C NMR (100 MHz, CDCl_3_) δ 146.3, 138.4, 138.0, 137.9, 137.7, 134.3, 132.9, 129.8, 129.4, 129.1, 128.7, 128.58, 128.56, 128.53, 128.49, 128.4, 128.02, 127.96, 127.9, 127.84, 127.75, 127.6, 127.5, 125.8, 122.2, 84.5, 77.5, 77.2, 75.5, 74.72, 74.66, 73.8, 73.6, 72.3, 69.2, 51.2; ^19^F NMR (376 MHz, CD_3_OD) δ −62.48; HRMS (*m*/*z*): [M + H]^+^ calcd for 750.3162; found, 750.3155.

**3,4,5,7-Tetra-*O-*benzyl-1,2-dideoxy-1-(4-naphth-1-yl-1*H*-1,2,3-triazol-1-yl)-β-D-galactoheptulose (10l):** Compound **9** (228 mg, 0.393 mmol), 1-ethynylnaphtalene (63 mg, 0.413 mmol), and copper(I) iodide (15 mg, 0.079 mmol) were dissolved in dry acetonitrile (4 mL) under nitrogen, triethylamine (110 μL, 0.786 mmol) was added, and the reaction left at 60 °C overnight. Upon completion, the reaction mixture was poured into ethyl acetate (20 mL) and washed with brine (20 mL). The brine was extracted with ethyl acetate (2 × 20 mL), the organic phases pooled, dried with anhydrous sodium sulfate, and evaporated. The crude product was purified with column chromatography (heptane/ethyl acetate 3:1) to give **10l** (120 mg, 42%) as a sticky clear solid. [α]_D_^20^ −11 (*c* 1.1, CHCl_3_); ^1^H NMR (400 MHz, CDCl_3_) δ 8.40 (d, *J =* 8.5 Hz, 1H), 8.04 (s, 1H), 7.94–7.88 (m, 2H), 7.66 (dd, *J =* 7.2 Hz, 1.1 Hz, 1H), 7.54–7.12 (m, 23H), 5.00 (d, *J =* 10.9 Hz, 1H), 4.94 (d, *J =* 11.8 Hz, 1H), 4.85–4.77 (m, 3H), 4.76–4.68 (m, 3H), 4.59 (d, *J =* 11.8 Hz, 1H), 4.47–4.44 (m, 2H), 4.01 (s, 1H, H5), 3.77–3.69 (m, 3H), 3.68–3.56 (m, 3H); ^13^C NMR (100 MHz, CDCl_3_) δ 146.6, 138.4, 138.04, 137.95, 137.7, 133.9, 131.2, 128.63, 128.55, 128.52, 128.46, 128.4, 128.3, 128.2, 128.0, 127.9, 127.8, 127.61, 127.55, 127.2, 126.5, 125.9, 125.6, 125.4, 124.5, 84.6, 77.7, 77.2, 75.4, 74.9, 74.6, 73.8, 73.6, 72.3, 69.0, 51.1; HRMS (*m*/*z*): [M + H]^+^ calcd for 732.3442; found, 732.3437.

**3,4,5,7-Tetra-*O-*benzyl-1,2-dideoxy-1-[4-(4-biphenyl)-1*H*-1,2,3-triazol-1-yl]-β-D-galactoheptulose (10m):** Compound **9** (103 mg, 0.178 mmol), 4-ethynylbiphenyl (38 mg (0.213 mmol), and copper(I) iodide (4 mg, 0.035 mmol) were dissolved in dry acetonitrile (3 mL) under nitrogen, triethylamine (50 μL, 0.356 mmol) was added, and the reaction left at room temperature overnight. Upon completion, the reaction mixture was poured into ethyl acetate (20 mL) and washed with brine (20 mL). The brine was extracted with ethyl acetate (2 × 20 mL), the organic phases pooled, dried with anhydrous sodium sulfate, and evaporated. The crude product was purified with column chromatography (heptane/ethyl acetate 2:1) to give **10m** (72 mg, 53%) as a white solid. [α]_D_^20^ −45 (*c* 1.0, CH_2_Cl_2_); ^1^H NMR (400 MHz, CDCl_3_) δ 8.03 (s, 1H), 7.86–7.80 (m. 2H), 7.69–7.58 (m, 4H), 7.53–7.46 (m, 2H), 7.44–7.15 (m, 21H), 4.97 (d, *J =* 7.1 Hz, 1H), 4.94 (d, *J =* 6.1 Hz, 1H), 4.80 (d, *J =* 11.7 Hz, 1H), 4.76–4.69 (m, 4H), 4.56 (d, *J =* 11.5 Hz, 1H), 4.52–4.48 (m, 2H), 4.00 (d, *J =* 1.9 Hz, 1H, H5), 3.77–3.55 (m, 6H); ^13^C NMR (100 MHz, CDCl_3_) δ 147.4, 140.8, 140.6, 138.5, 138.0, 137.9, 137.7, 129.9, 128.8, 128.54, 128.53, 128.50, 128.3, 128.0, 127.90, 127.85, 127.58, 127.55, 127.51, 127.48, 127.4, 127.0, 126.11, 121.4, 84.5, 77.7, 772, 75.4, 74.8, 74.6, 73.7, 73.6, 72.2, 69.1, 51.1; HRMS (*m*/*z*): [M + H]^+^ calcd for 758.3600; found, 758.3594.

**3,4,5,7-Tetra-*O-*benzyl-1,2-dideoxy-1-(4-napht-2-yl-1*H*-1,2,3-triazol-1-yl)-β-D-galactoheptulose (10n):** Compound **9** (150 mg, 0.259 mmol), 2-ethynylnaphthalene (43 mg, 0.285 mmol), and copper(I) iodide (10 mg, 0.052 mmol) were dissolved in dry acetonitrile (4 mL) under nitrogen, triethylamine (72 μL, 0.518 mmol) was added, and the reaction left at 60 °C overnight. Upon completion, the reaction mixture was poured into ethyl acetate (20 mL) and washed with brine (20 mL). The brine was extracted with ethyl acetate (2 × 20 mL), the organic phases pooled, dried with anhydrous sodium sulfate, and evaporated. The crude product was purified with column chromatography (heptane/ethyl acetate 3:1) to give **10n** (143 mg of product with a yield of, 75%) as a sticky clear solid. [α]_D_^20^ −25 (*c* 1.6,CH_3_CN); ^1^H NMR (400 MHz, CDCl_3_) δ 8.33 (s, 1H), 8.11 (s, 1H), 7.90–7.84 (m, 4H), 7.55–7.48 (m, 2H), 7.47–7.27 (m, 15H), 7.26–7.21 (m, 2H), 7.18–7.13 (m, 3H), 4.98 (d, *J =* 3.6 Hz, 1H), 4.95 (d, *J =* 4.7 Hz, 1H), 4.83–4.71 (m, 5H), 4.57 (d, *J =* 11.7 Hz, 1H), 4.51 (s, 2H), 4.01 (d, *J =* 0.9 Hz, 1H, H5), 3.75–3.58 (m, 6H); ^13^C NMR (100 MHz, CDCl_3_) δ 147.7, 138.5, 138.0, 137.9, 137.8, 133.6, 133.1, 128.7, 128.54, 128.49, 128.34, 128.28, 128.2, 128.0, 127.9, 127.8, 127.7, 127.59, 127.57, 127.5, 126.3, 126.0, 124.4, 124.0, 121.7, 84.6, 77.8, 77.2, 75.4, 74.9, 74.6, 73.7, 73.6, 73.3, 72.3, 69.1, 51.2; HRMS (*m*/*z*): [M + H]^+^ calcd for 732.3443; found, 732.3443.

### Fluorescence polarization experiments

Human galectin-1 [36] and galectin-3 [37] were expressed and purified as earlier described. Fluorescence polarization experiments were performed on a PheraStarFS plate reader with software PHERAstar Mars version 2.10 R3 (BMG, Offenburg, Germany). Specific experimental conditions were a galectin-1 concentration of 0.5 µM together with the fluorescent probe (3,3’-dideoxy-3-[4-(fluorescein-5-ylcarbonylaminomethyl)-1*H*-1,2,3-triazol-1-yl]-3’-(3,5-dimethoxybenzamido)-1,1’-sulfanediyl-di-β-D-galactopyranoside [36]) concentration of 20 nM and a galectin-3 concentration of 0.2 µM together with the fluorescent probe (3,3’-dideoxy-3-[4-(fluorescein-5-ylcarbonylaminomethyl)-1*H*-1,2,3-triazol-1-yl]-3’-(3,5-dimethoxybenzamido)-1,1’-sulfanediyl-di-β-D-galactopyranoside) concentration of 20 nM, respectively. Inhibitors were dissolved in dimethyl sulfoxide (analytical grade) to a concentration of 20 mM, diluted with PBS to 3–6 different concentrations, and tested in duplicate twice for galectin affinity using a competitive fluorescence polarization assay as earlier described [33]. The highest inhibitor concentrations tested were 1.5 mM due to solubility limitations at higher concentration. Dissociation constants average and SEM were calculated from two to eight single point measurements showing between 20–80% inhibition.

### Molecular modelling

Molecular dynamics simulations were performed with the OPLS3 force field in Desmond Desmond (Schrödinger Release 2017-3: Desmond Molecular Dynamics System, D. E. Shaw Research, New York, NY, 2017. Maestro-Desmond Interoperability Tools, Schrödinger, New York, NY, 2017) using default settings except for the length of the simulation and the use of light harmonic constraints (1 kcal/mol/Å) on all stranded backbone atoms and on the galactose O4 atom. Non-minimized conformations of **1b** were positioned to replace lactose in the binding site of galectin-1 (pdb id 1GWZ) with the galactose ring in an orientation identical to that in lactose and with the phenyl triazole rings extending into bulk between the Trp68-Gly69-Thr70 and His52 was subjected to a 200 ns molecular dynamics simulation. Non-minimized conformations of **1b** were positioned to replace lactose in the binding site of galectin-3 (pdb id 1KJL) with the galactose ring in an orientation identical to that in *N*-acetyllactosamine and with the phenyl triazole rings extending into bulk between the Trp68-Gly69-Thr70 and His52 was subjected to a 200 ns molecular dynamics simulation. Energy minimizations of four different MD snapshots of **1b** in complex with galectin-1 and with galectin-3 were performed with the OPLS3 force field in MacroModel (performed with the OPLS3 force field in MacroModel (Schrödinger Release 2017-3: MacroModel, Schrödinger, LLC, New York, NY, 2017) using default settings.

## Supporting Information

File 1 Copies of ^1^H NMR and ^13^C NMR spectra for compounds **1**–**10**.
