# Supplementary material for: Stereo- and regioselective hydroboration of 1-exo-methylene pyranoses: discovery of aryltriazolylmethyl C-galactopyranosides as selective galectin-1 inhibitors
Source: Beilstein J Org Chem. 2019 May 7;15:1046–60. doi: 10.3762/bjoc.15.102 (PMC6541369; doi:10.3762/bjoc.15.102)
Supplement: File 1 — Copies of 1H NMR and 13C NMR spectra for compounds 1–10. [file Beilstein_J_Org_Chem-15-1046-s001.pdf]

BEILSTEIN JOURNAL OF ORGANIC CHEMISTRY

Supporting Information

for

Stereo- and regioselective hydroboration of 1-*exo*-methylene pyranoses: discovery of aryltriazolylmethyl C-galactopyranosides as selective galectin-1 inhibitors

Alexander Dahlqvist, Axel Furevi, Niklas Warlin, Hakon Leffler and Ulf J. Nilsson

Beilstein J. Org. Chem. **2019,** *15,* 1046–1060. doi:10.3762/bjoc.15.102

Copies of ^{1}H NMR and ^{13}C NMR spectra for compounds 1–10

License and Terms: This is a supporting information file under the terms of the Creative Commons Attribution License (http://creativecommons.org/licenses/by/4.0). Please note that the reuse, redistribution and reproduction in particular requires that the authors and source are credited.
The license is subject to the *Beilstein Journal of Organic Chemistry* terms and conditions: (https://www.beilstein-journals.org/bjoc)

Compound **1a**:

^{1}H:

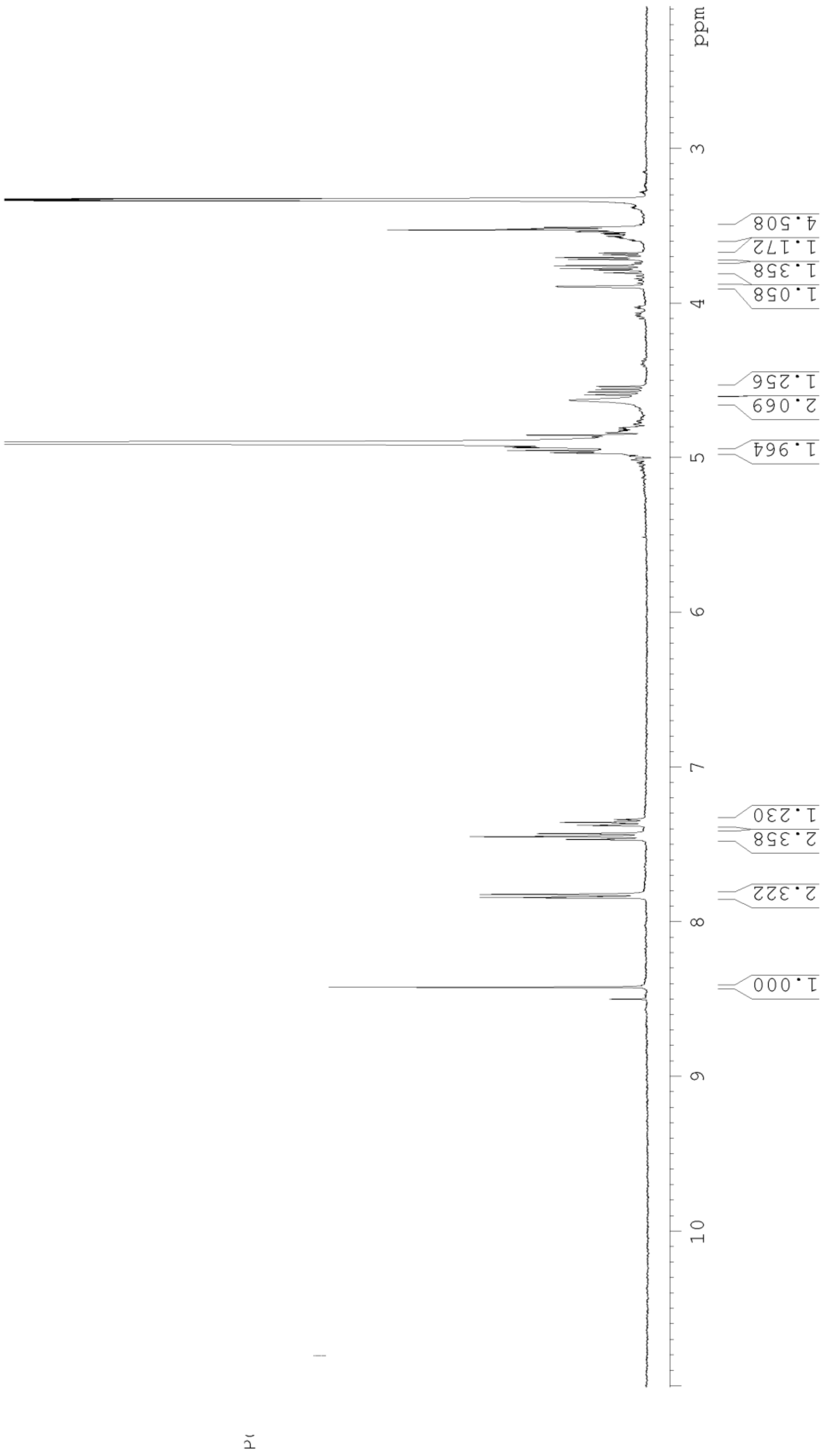

^{13}C:

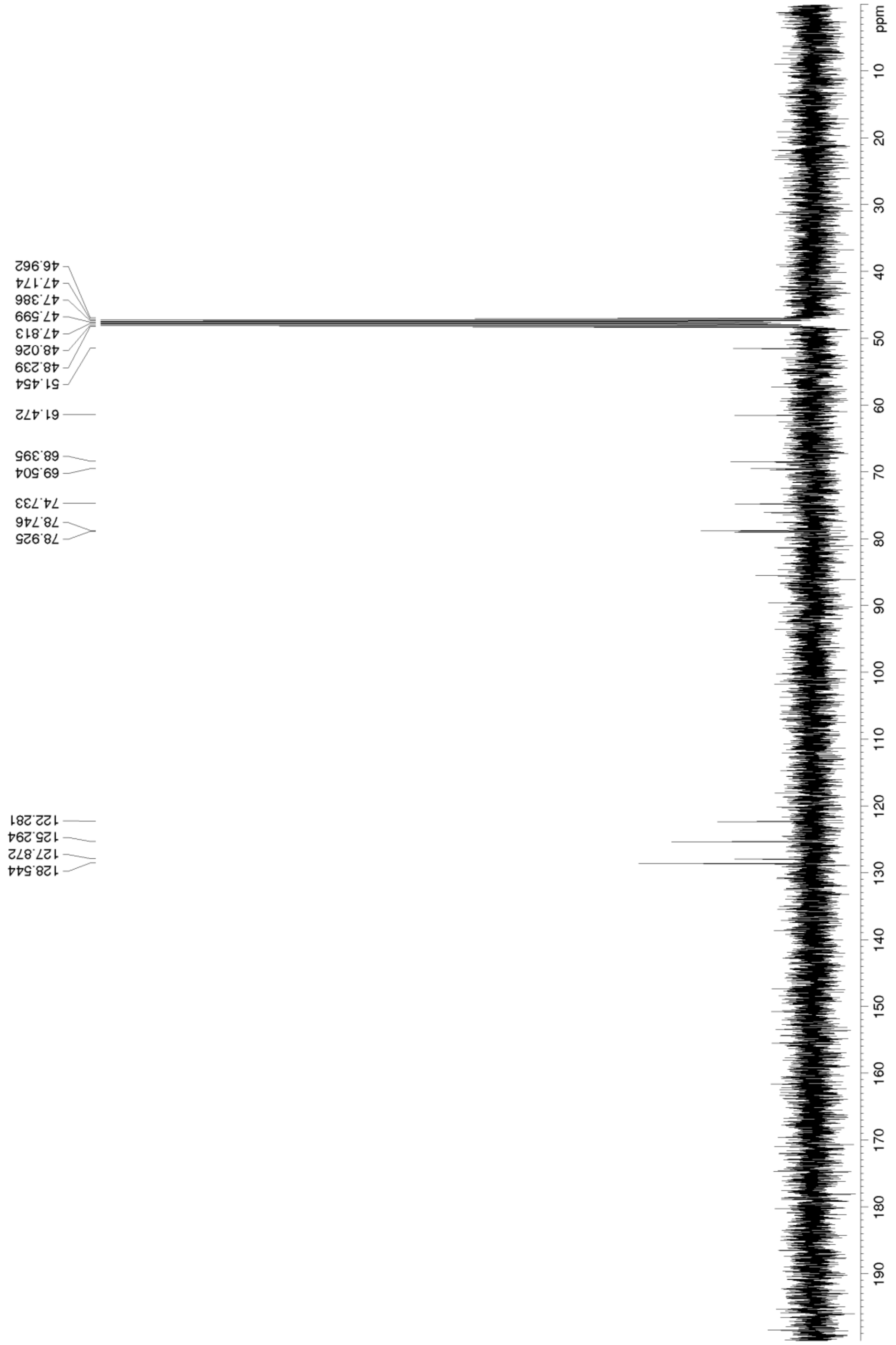
46.962
47.174
47.386
47.599
47.813
48.026
48.239
51.454
61.472
68.395
69.504
74.733
78.746
78.925
122.281
125.294
127.872
128.544
ppm
10
20
30
40
50
60
70
80
90
100
110
120
130
140
150
160
170
180
190

Compound **1b**:

^{1}H:

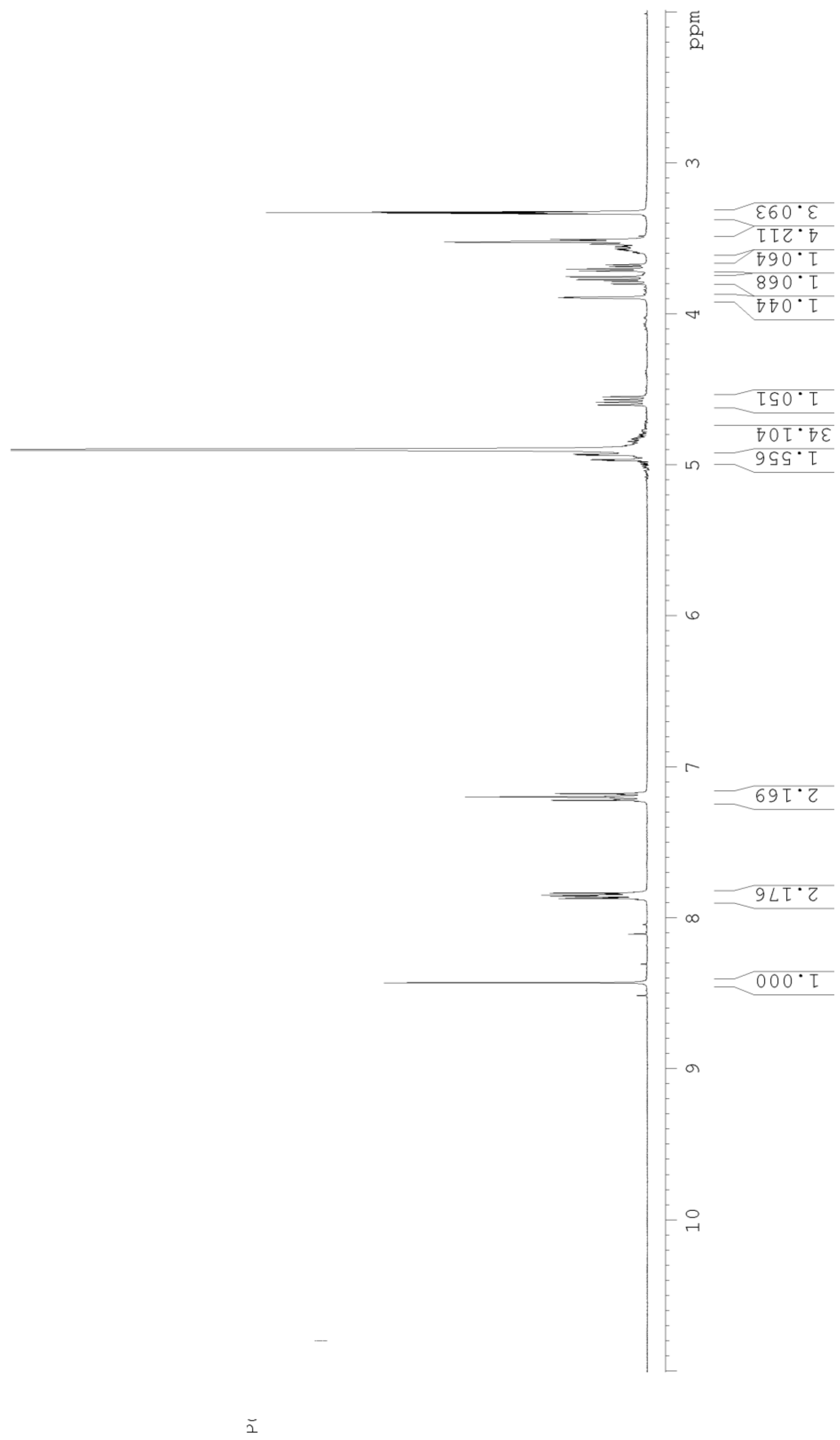

^{13}C:

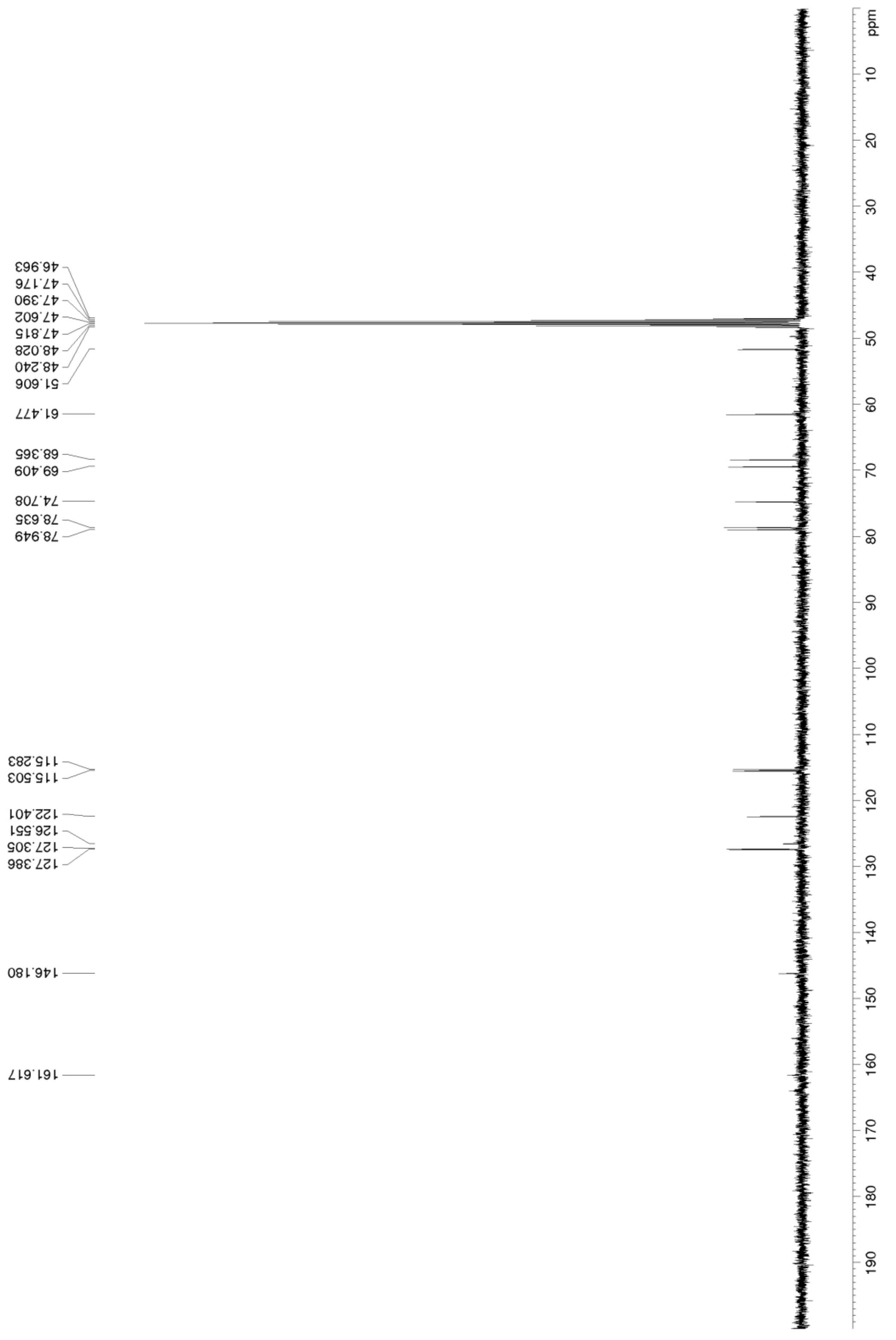
46.963
47.176
47.390
47.602
47.815
48.028
48.240
51.606
61.477
68.365
69.409
74.708
78.635
78.949
115.283
115.503
122.401
126.551
127.305
127.386
146.180
161.617
ppm

Compound **1c**:

^{1}H:

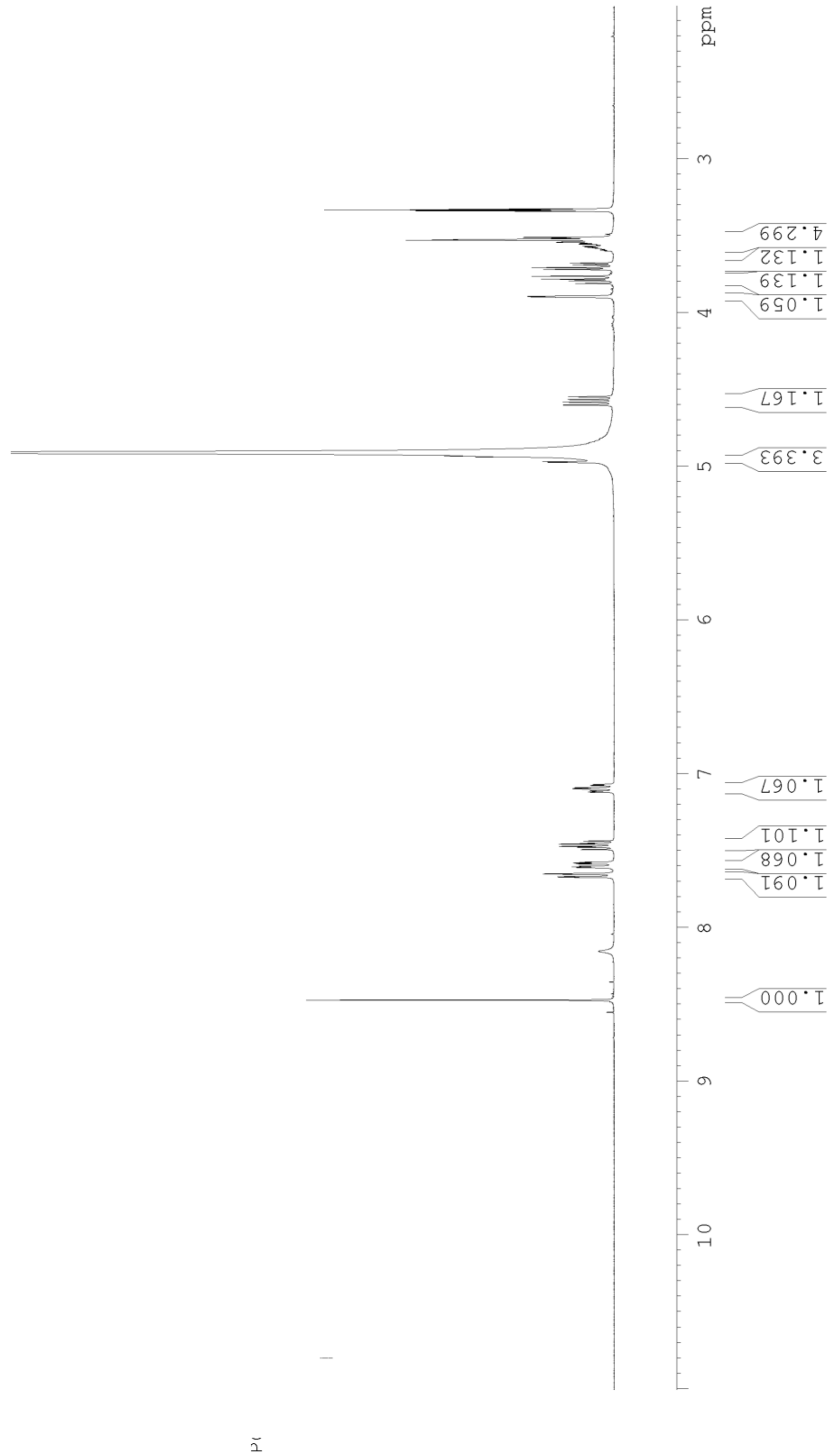

^{13}C:

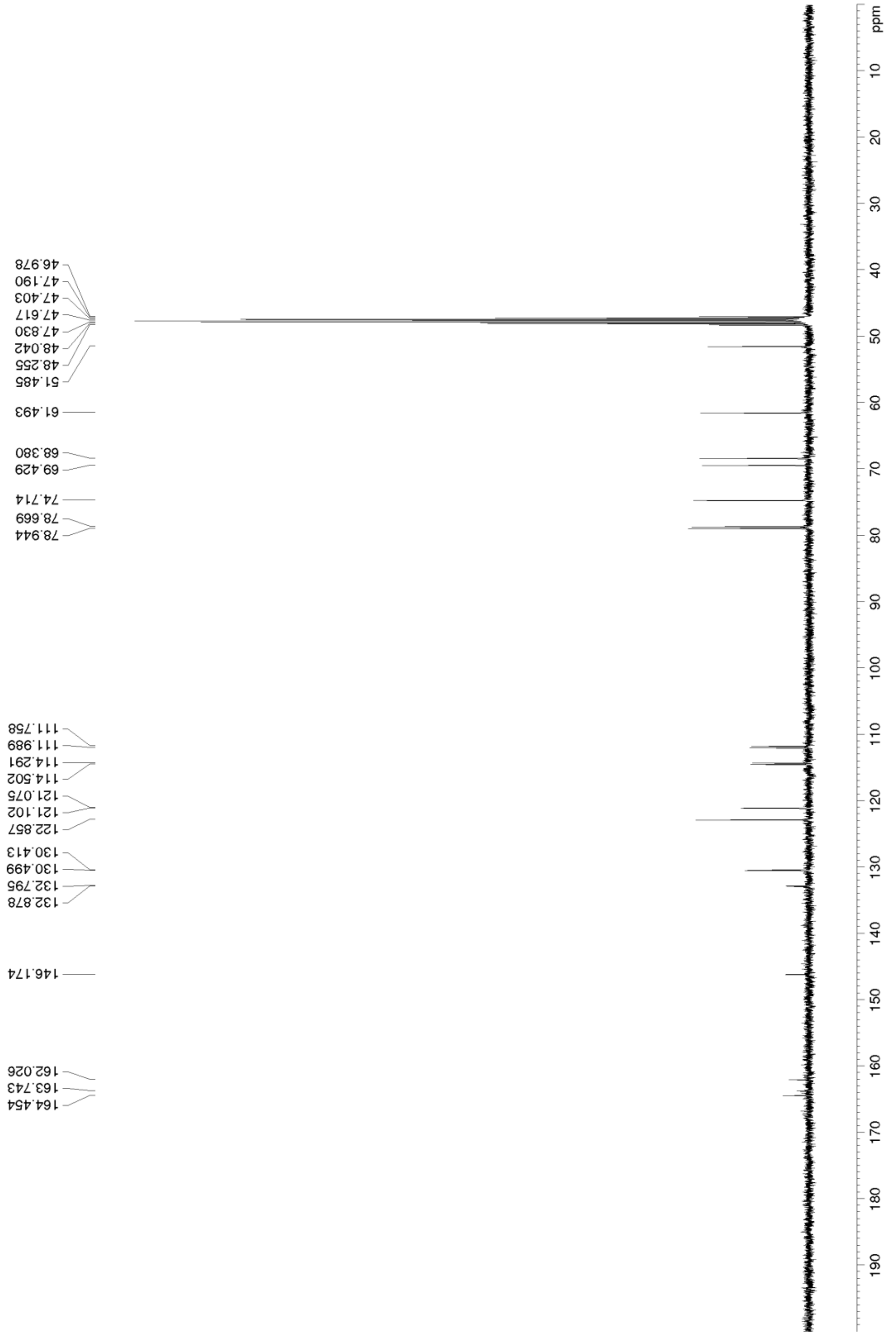
46.978
47.190
47.403
47.617
47.830
48.042
48.255
51.485
61.493
68.380
69.429
74.714
78.669
78.944
111.758
111.989
114.291
114.502
121.075
121.102
122.857
130.413
130.499
132.795
132.878
146.174
162.026
163.743
164.454
ppm

Compound **1d**:

^{1}H:

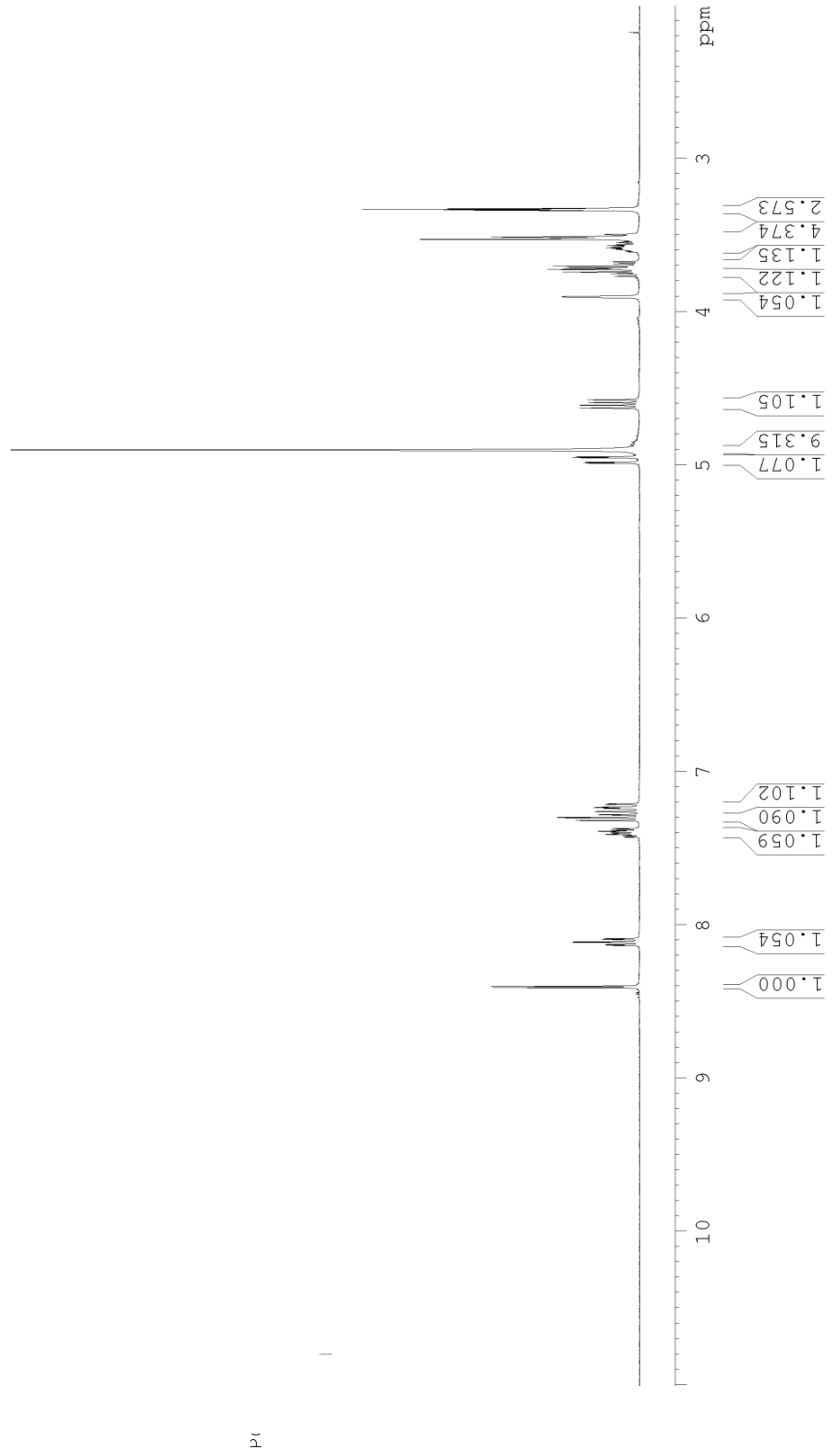

[13]C:

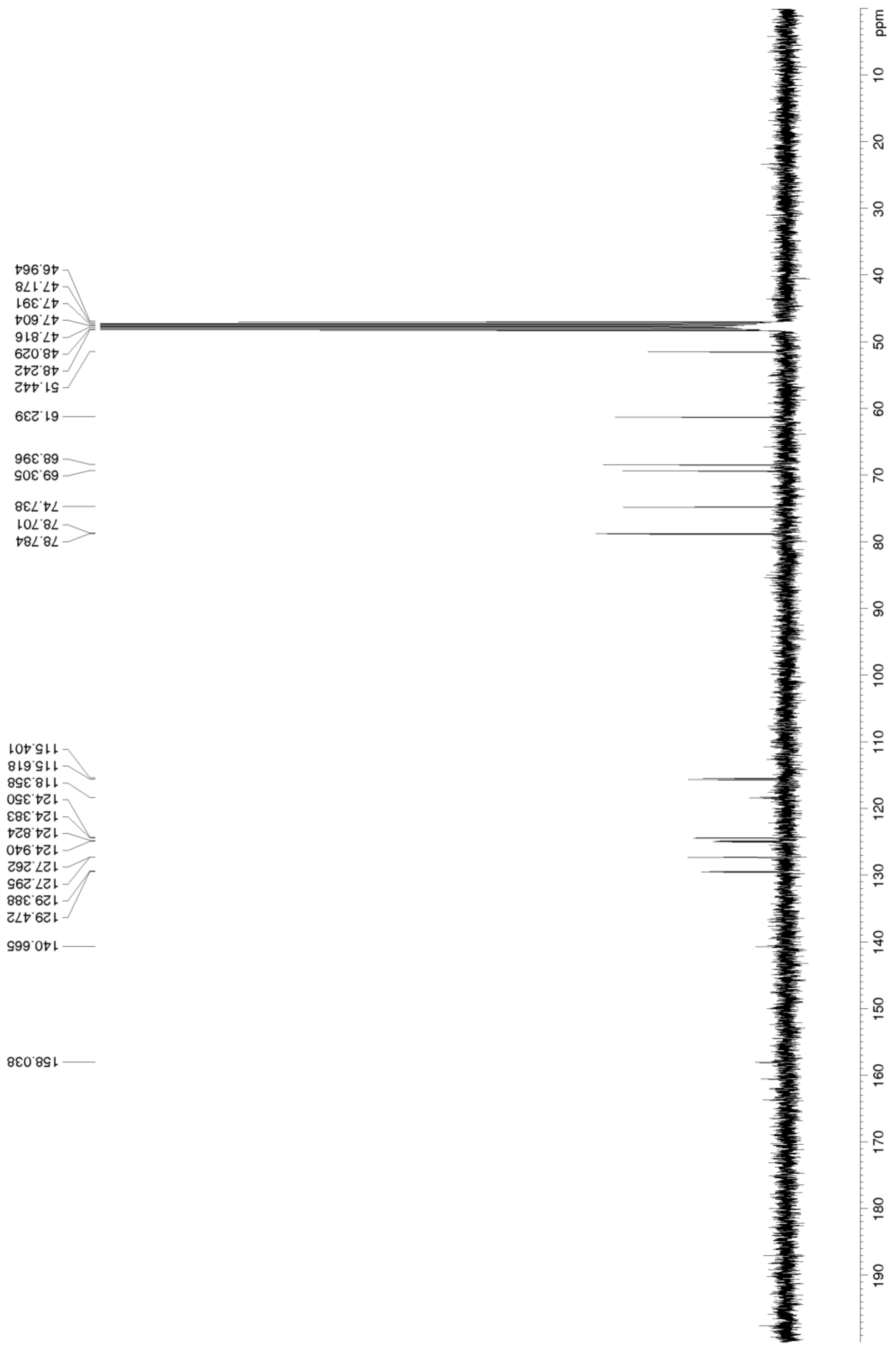
46.964
47.178
47.391
47.604
47.816
48.029
48.242
51.442
61.239
68.396
69.305
74.738
78.701
78.784
115.401
115.618
118.358
124.350
124.383
124.824
124.940
127.262
127.295
129.388
129.472
140.665
158.038
ppm
10
20
30
40
50
60
70
80
90
100
110
120
130
140
150
160
170
180
190

Compound **1e**:

^{1}H:

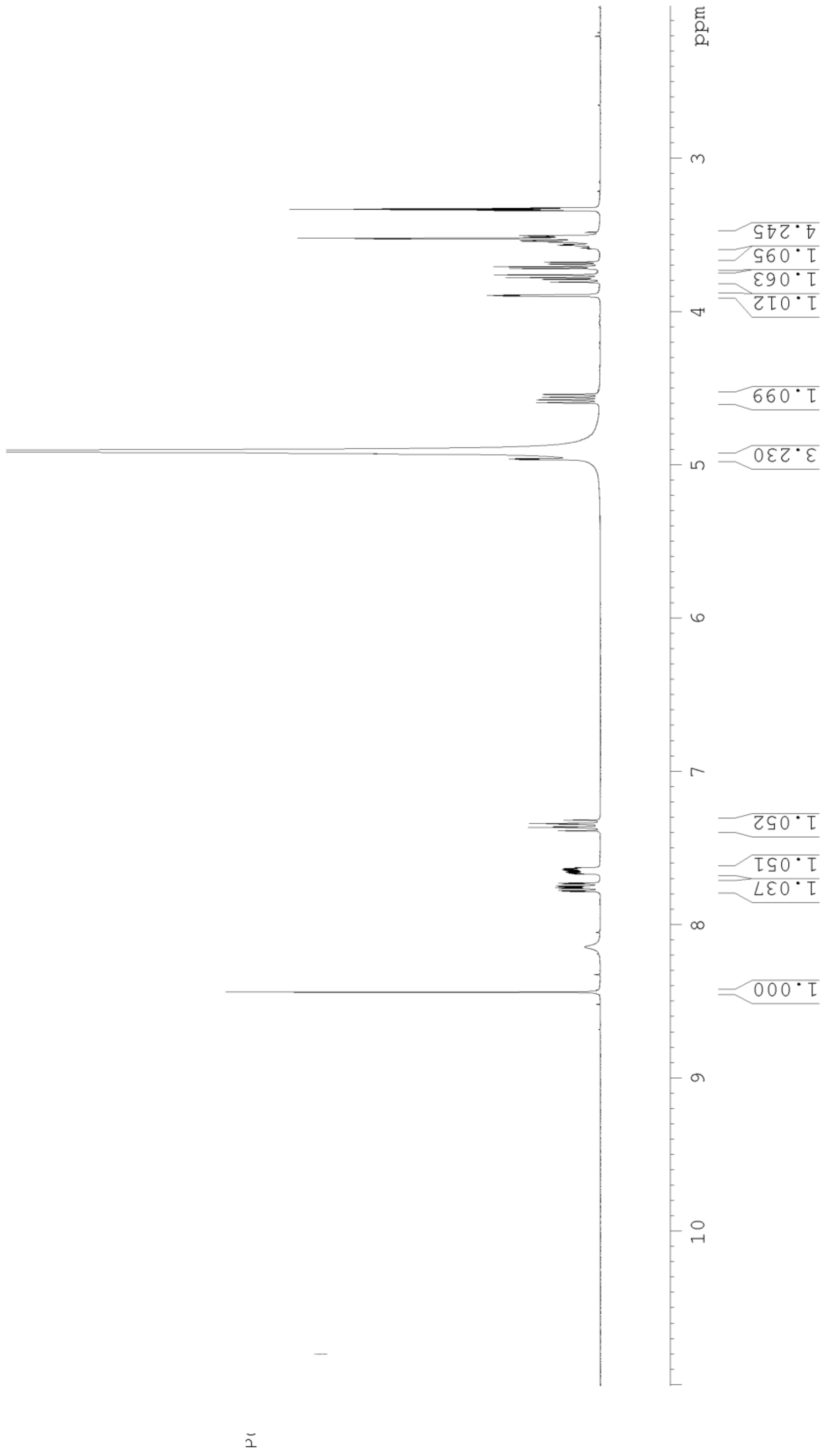

^{13}C:

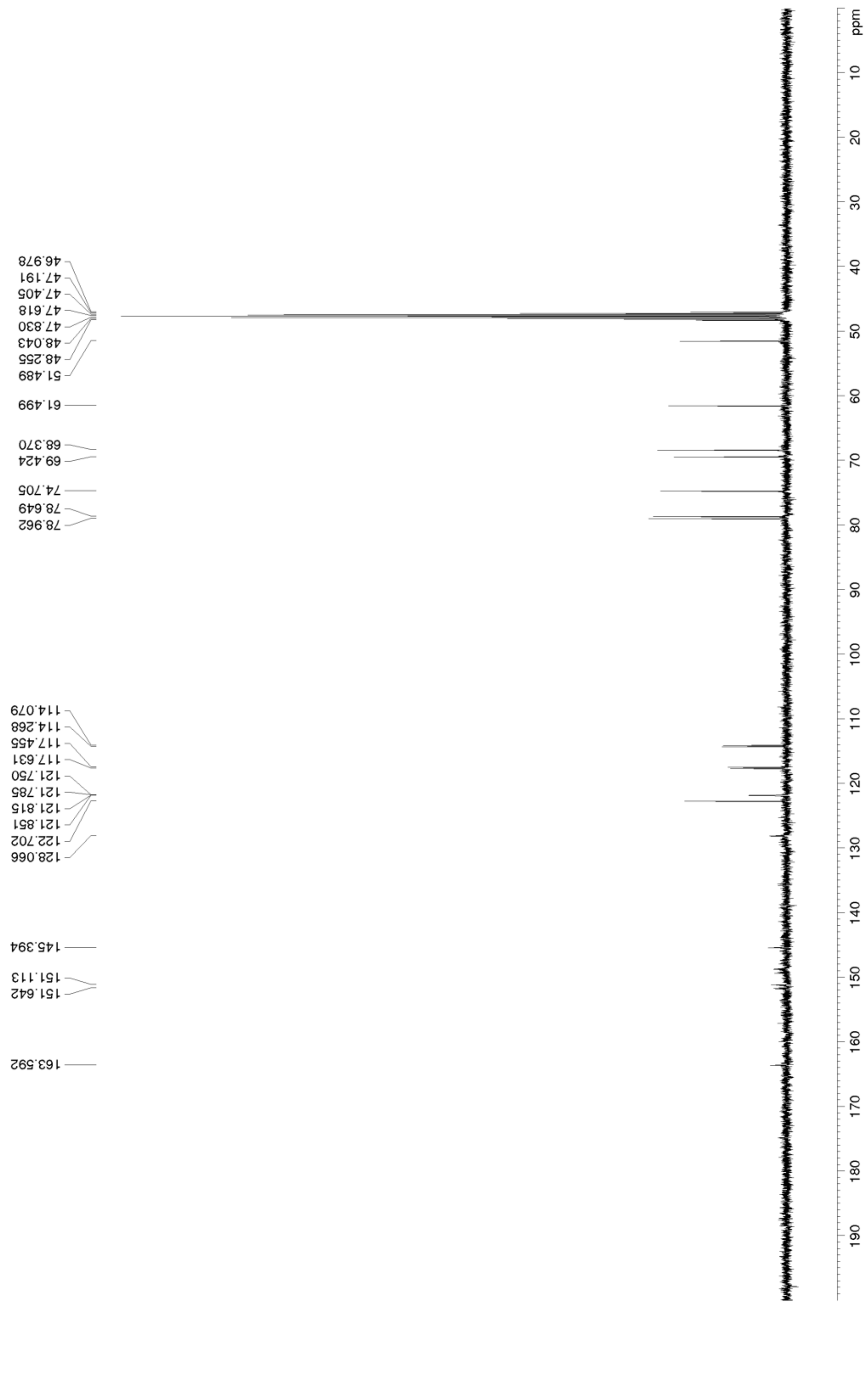
46.978
47.191
47.405
47.618
47.830
48.043
48.255
51.489
61.499
68.370
69.424
74.705
78.649
78.962
114.079
114.268
117.455
117.631
121.750
121.785
121.815
121.851
122.702
128.066
145.394
151.113
151.642
163.592
ppm
10
20
30
40
50
60
70
80
90
100
110
120
130
140
150
160
170
180
190

Compound **1f**:

^{1}H:

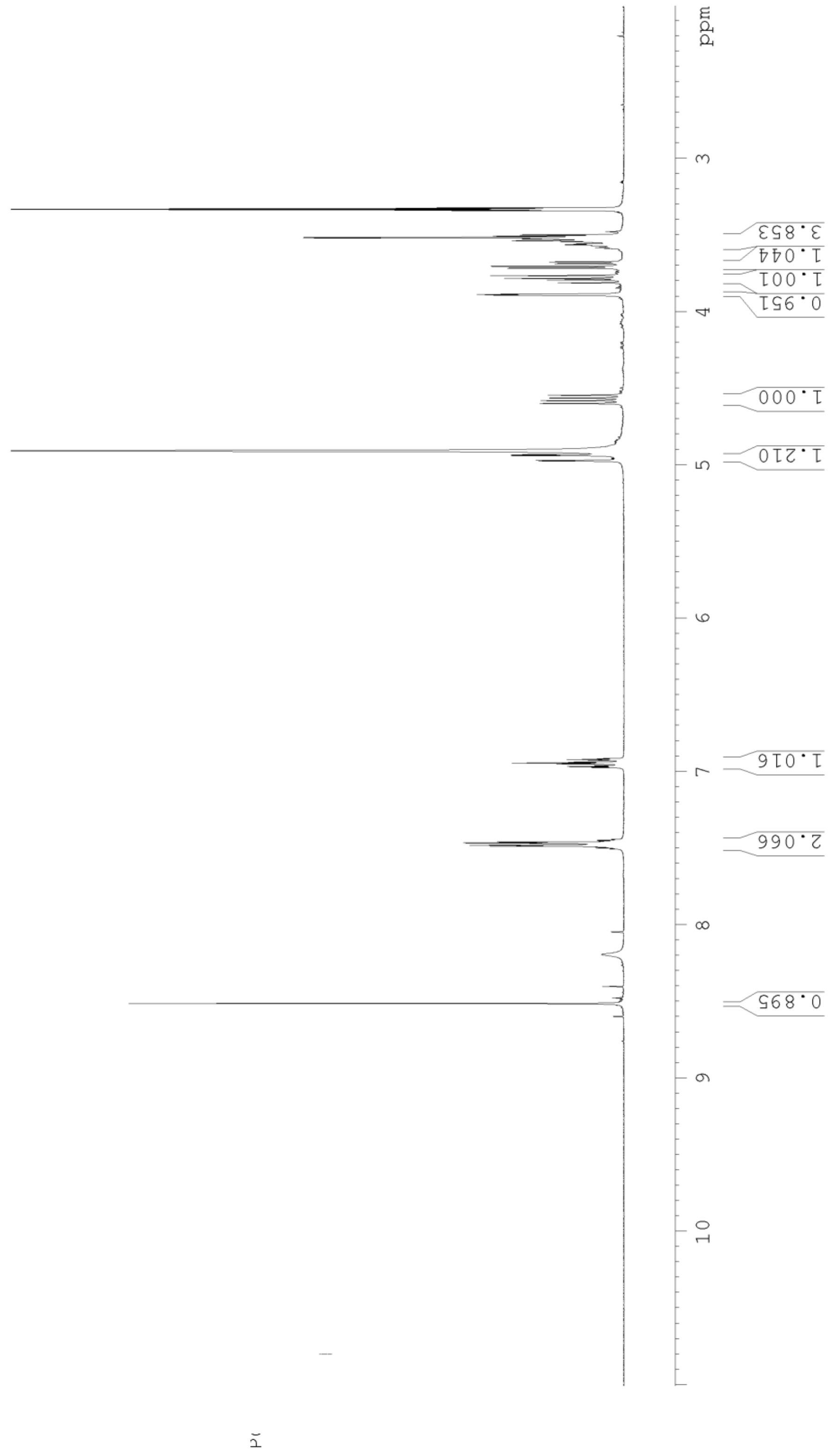

^{13}C:

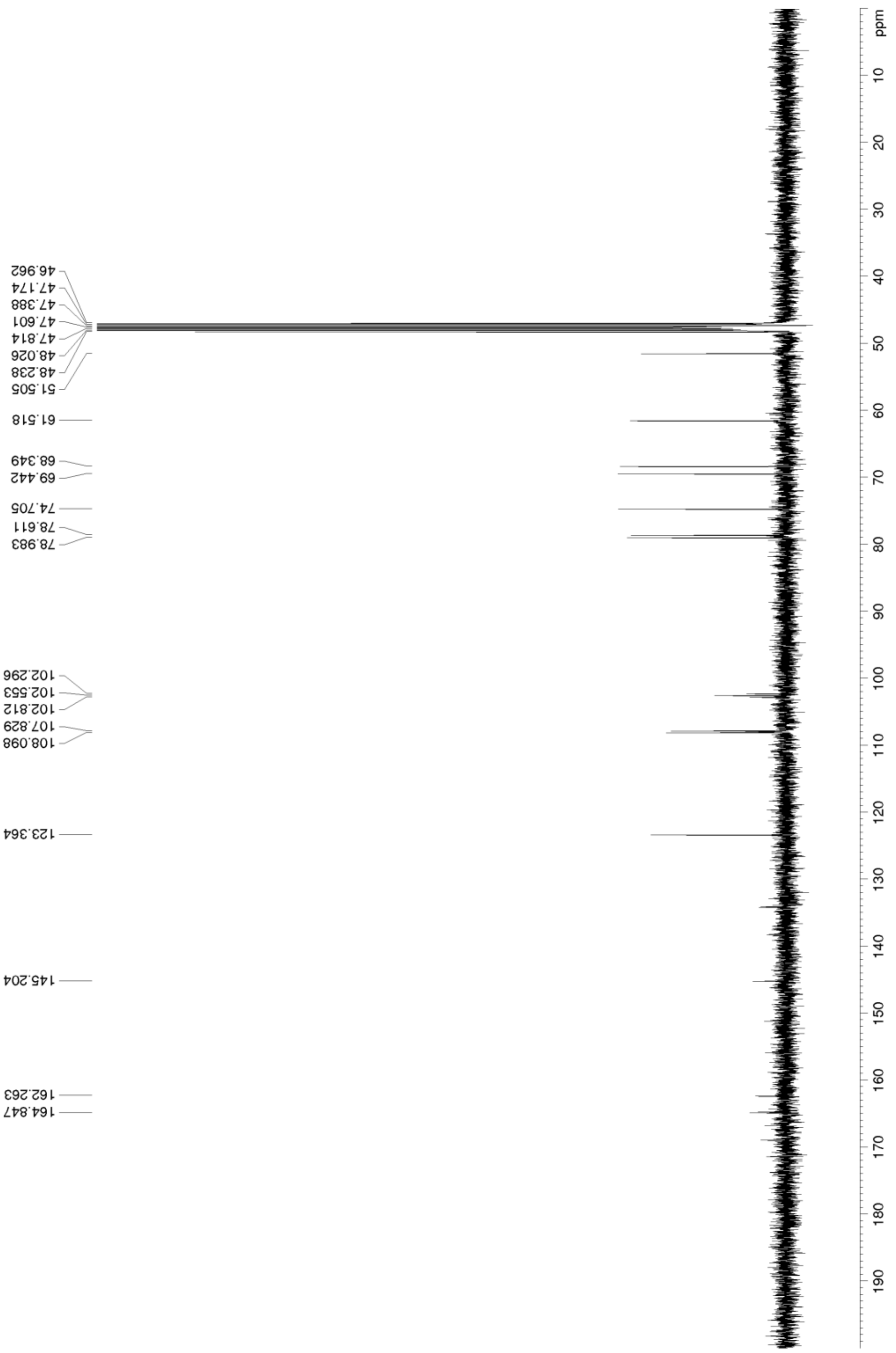
46.962
47.174
47.388
47.601
47.814
48.026
48.238
51.505
61.518
68.349
69.442
74.705
78.611
78.983
102.296
102.553
102.812
107.829
108.098
123.364
145.204
162.263
164.847
ppm

Compound **1g**:

^{1}H:

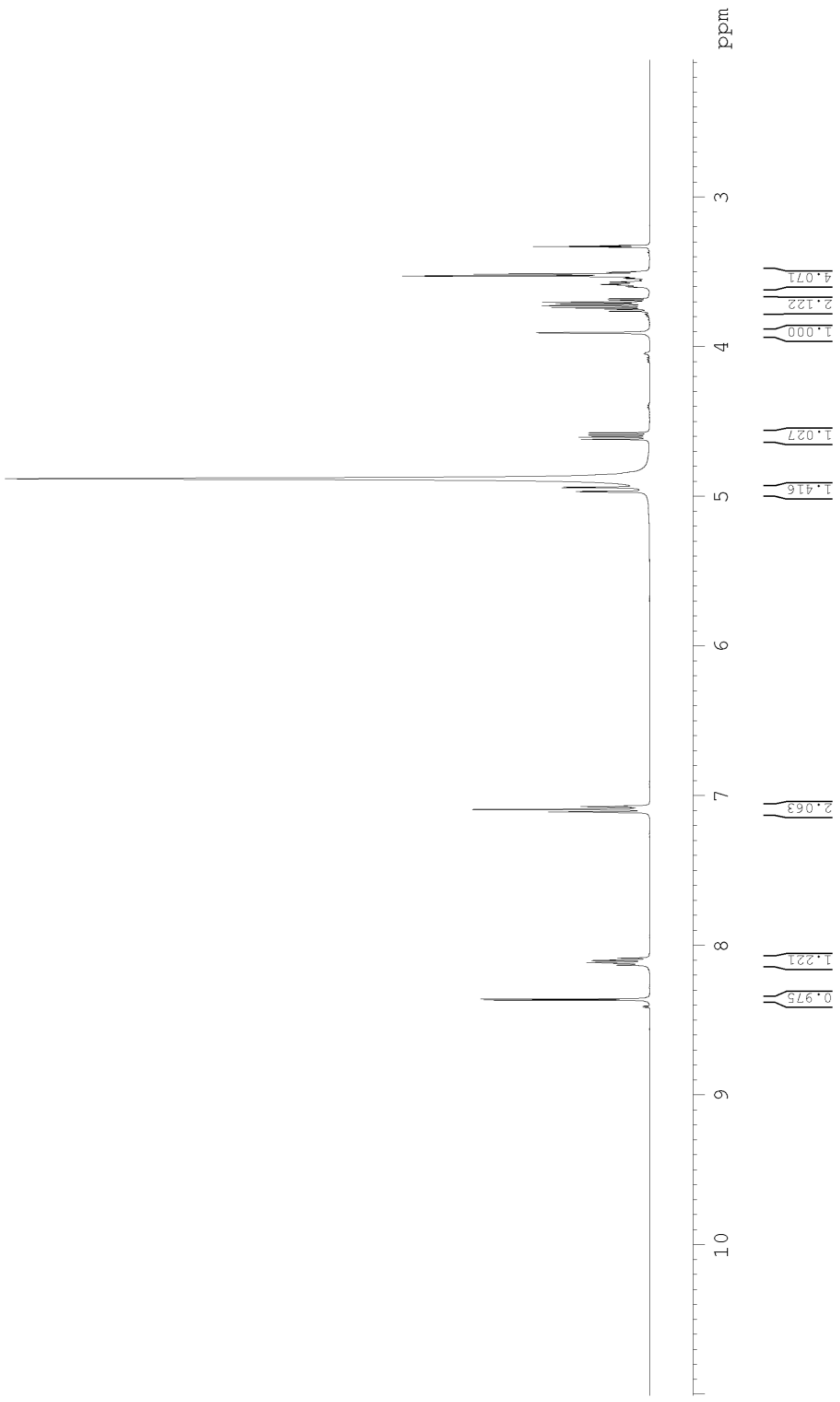

^{13}C:

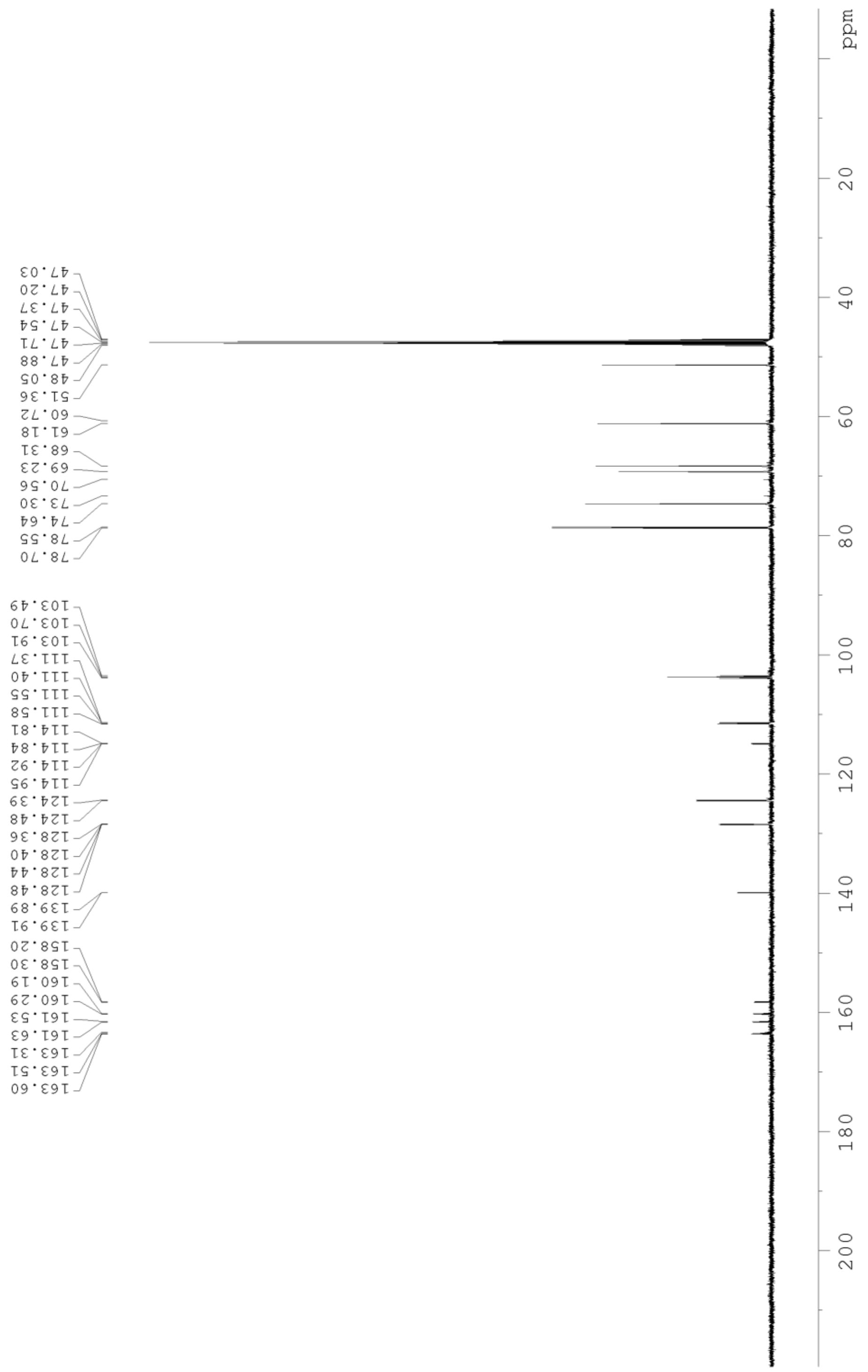

47.03
47.20
47.37
47.54
47.71
47.88
48.05
51.36
60.72
61.18
68.31
69.23
70.56
73.30
74.64
78.55
78.70
103.49
103.70
103.91
111.37
111.40
111.55
111.58
114.81
114.84
114.92
114.95
124.39
124.48
128.36
128.40
128.44
128.48
139.89
139.91
158.20
158.30
160.19
160.29
161.53
161.63
163.31
163.51
163.60
ppm
20
40
60
80
100
120
140
160
180
200

Compound **1h**:

^{1}H:

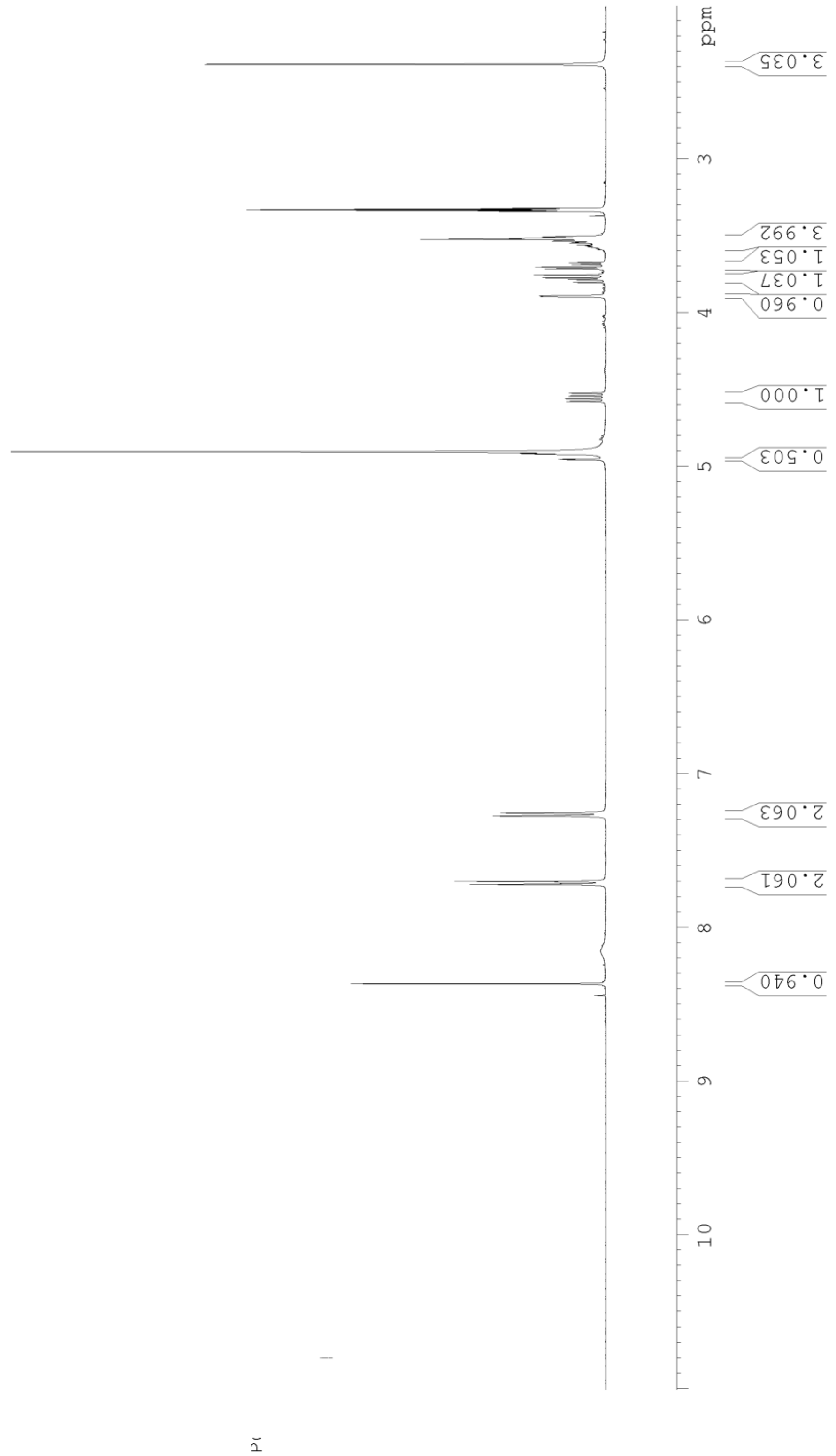

^{13}C:

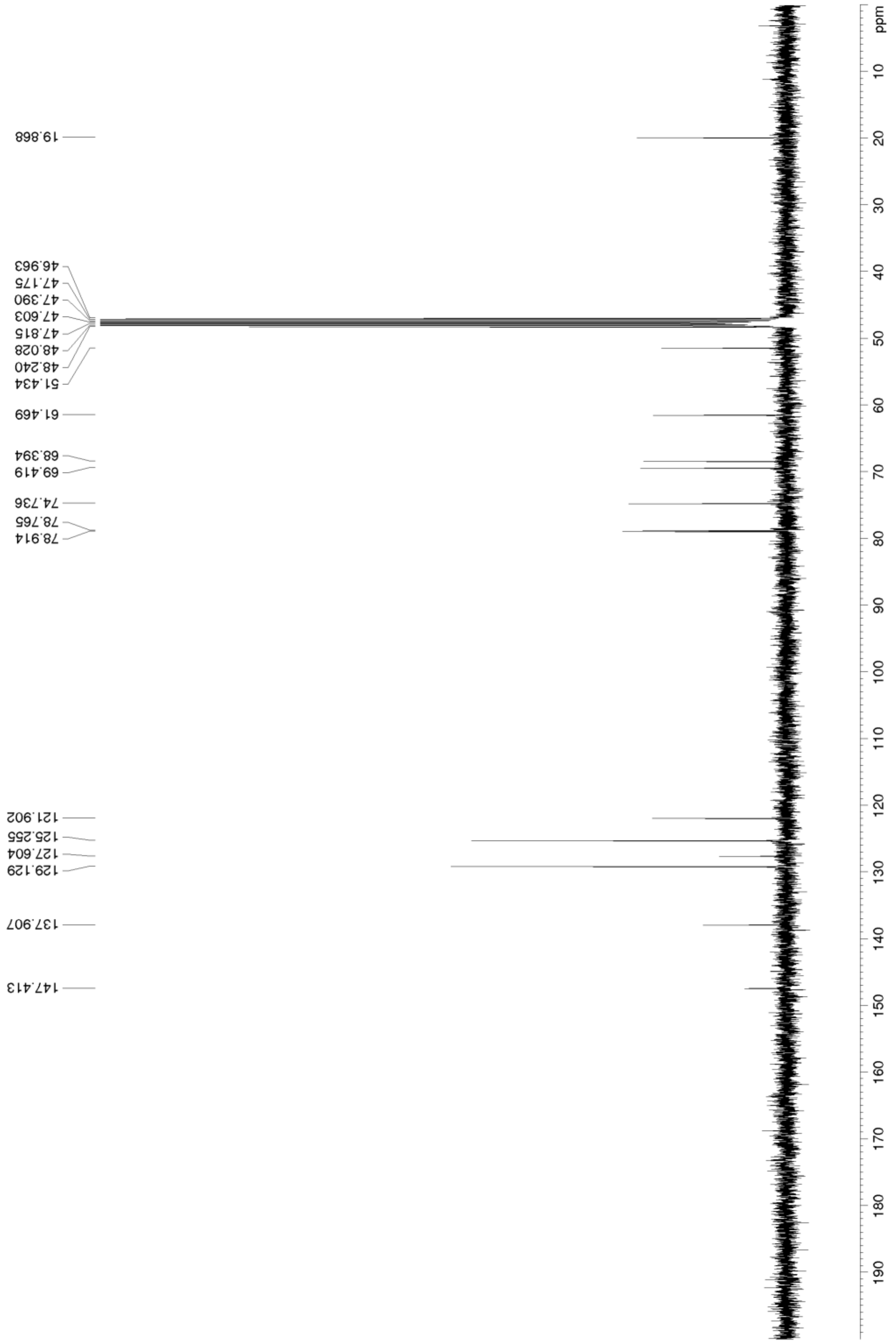

19.868
46.963
47.175
47.390
47.603
47.815
48.028
48.240
51.434
61.469
68.394
69.419
74.736
78.765
78.914
121.902
125.255
127.604
129.129
137.907
147.413
ppm

Compund **1i**:

^{1}H:

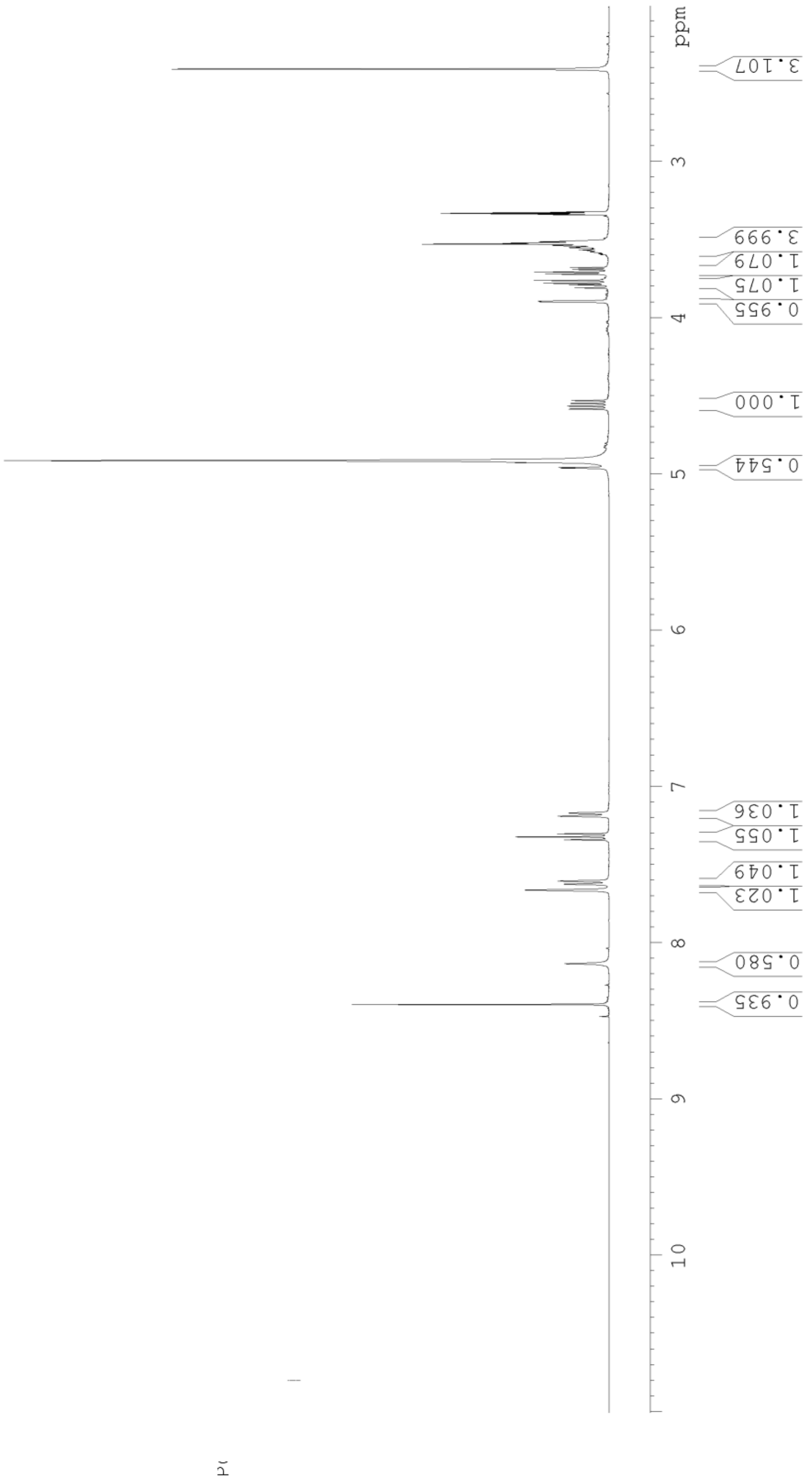

^{13}C:

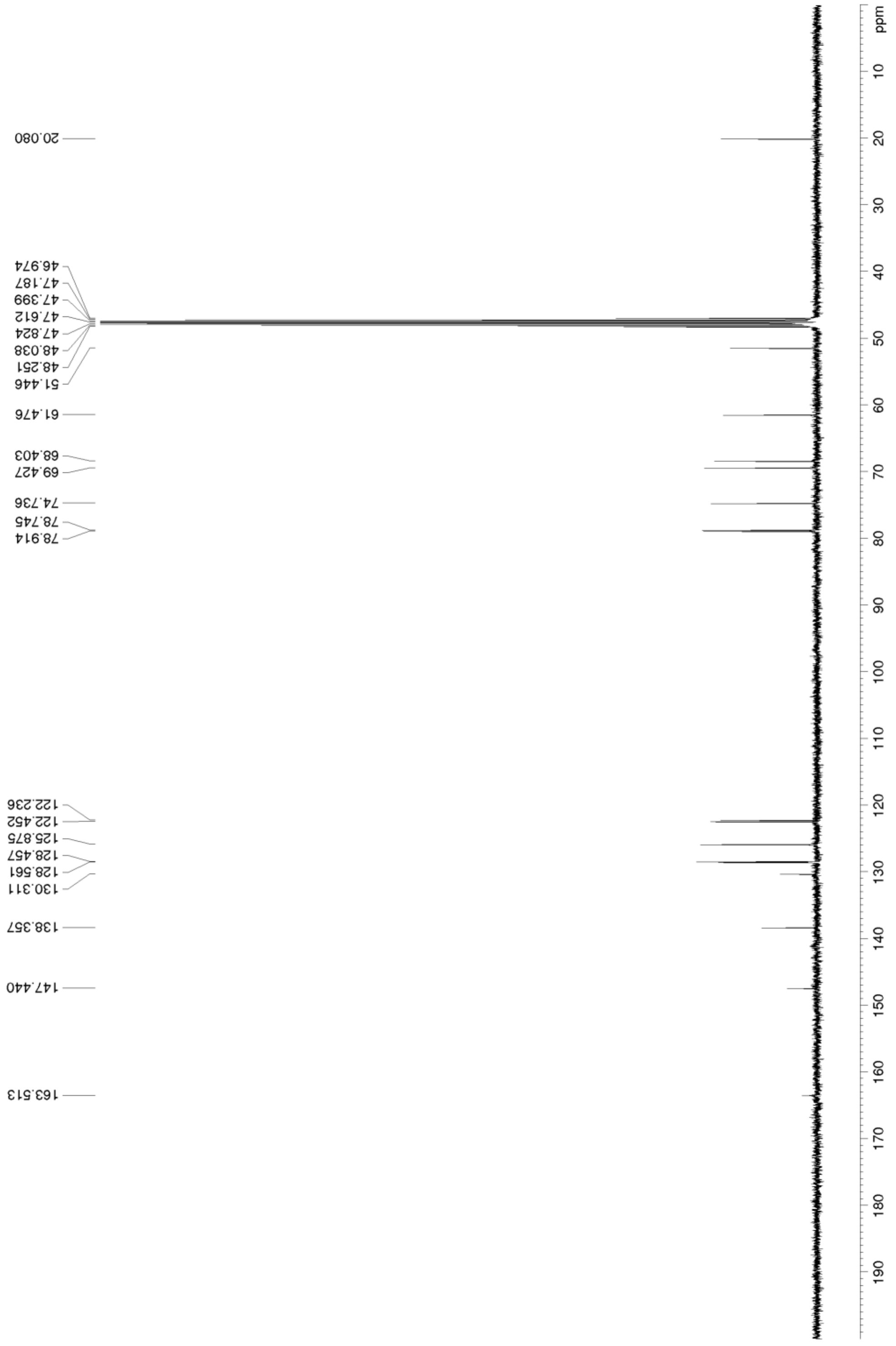
20.080
46.974
47.187
47.399
47.612
47.824
48.038
48.251
51.446
61.476
68.403
69.427
74.736
78.745
78.914
122.236
122.452
125.875
128.457
128.561
130.311
138.357
147.440
163.513
ppm

Compound **1j**:

^{1}H:

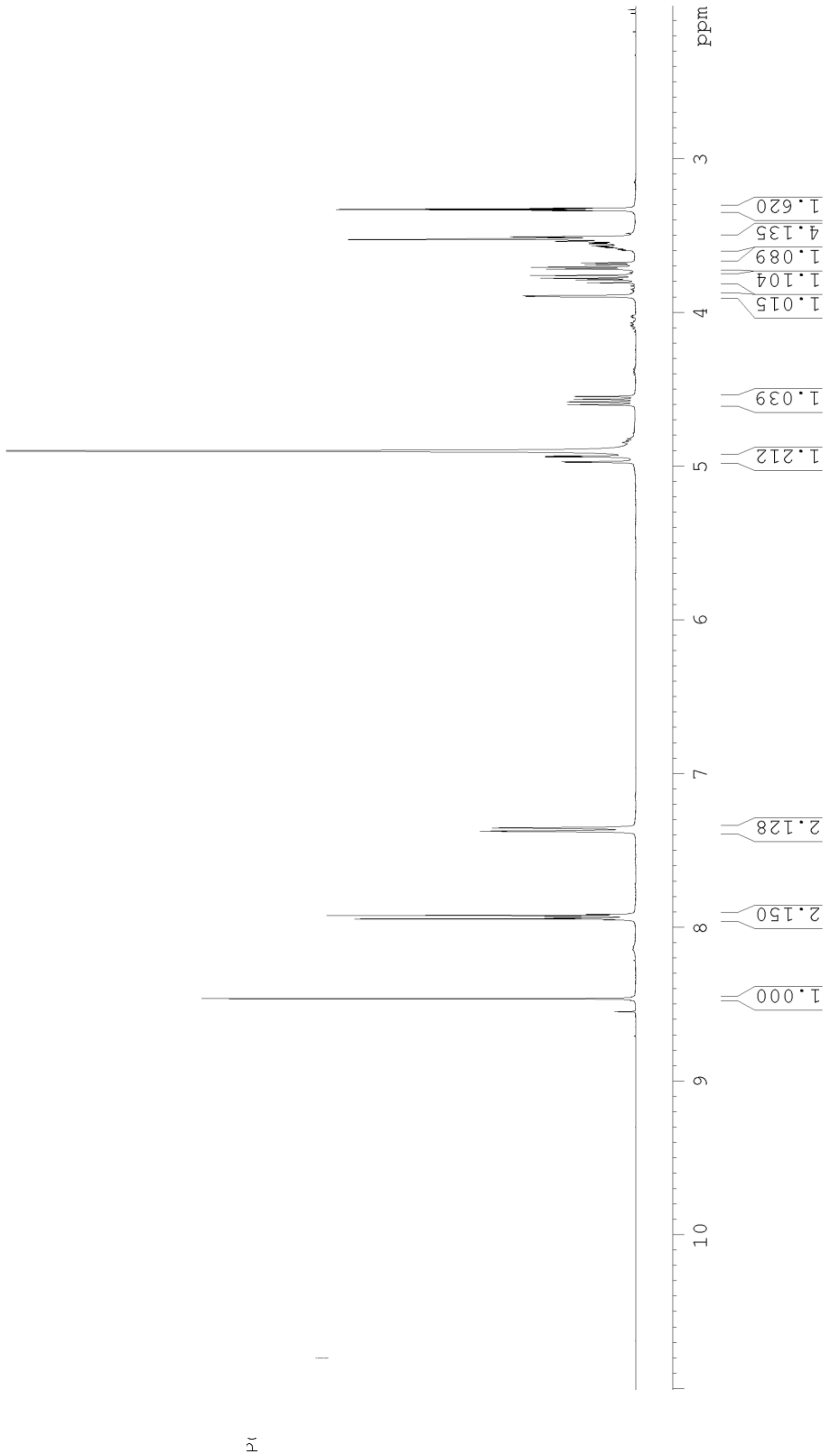

^{13}C:

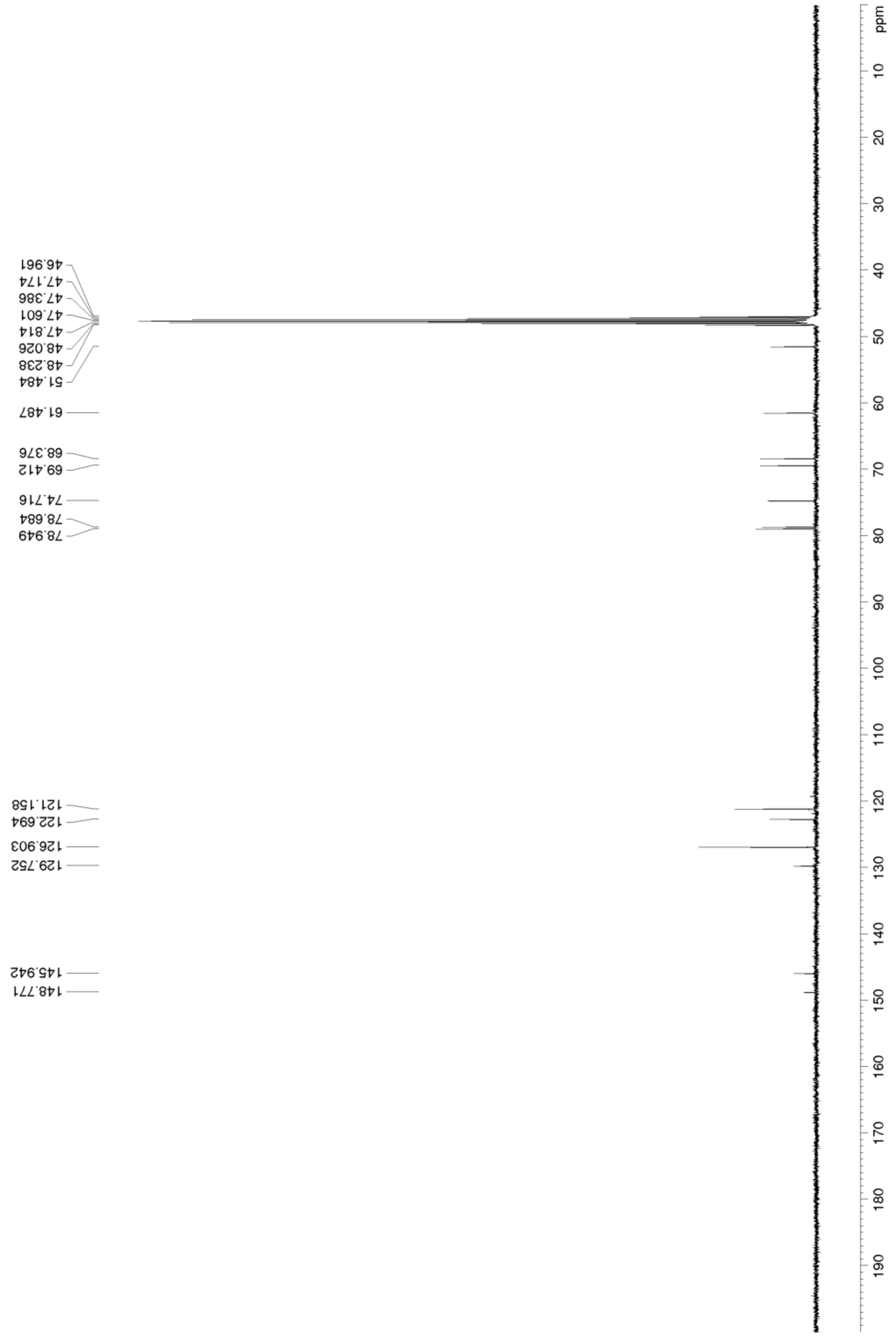
46.961
47.174
47.386
47.601
47.814
48.026
48.238
51.484
61.487
68.376
69.412
74.716
78.684
78.949
121.158
122.694
126.903
129.752
145.942
148.771
ppm
10
20
30
40
50
60
70
80
90
100
110
120
130
140
150
160
170
180
190

Compound **1k**:

^{1}H:

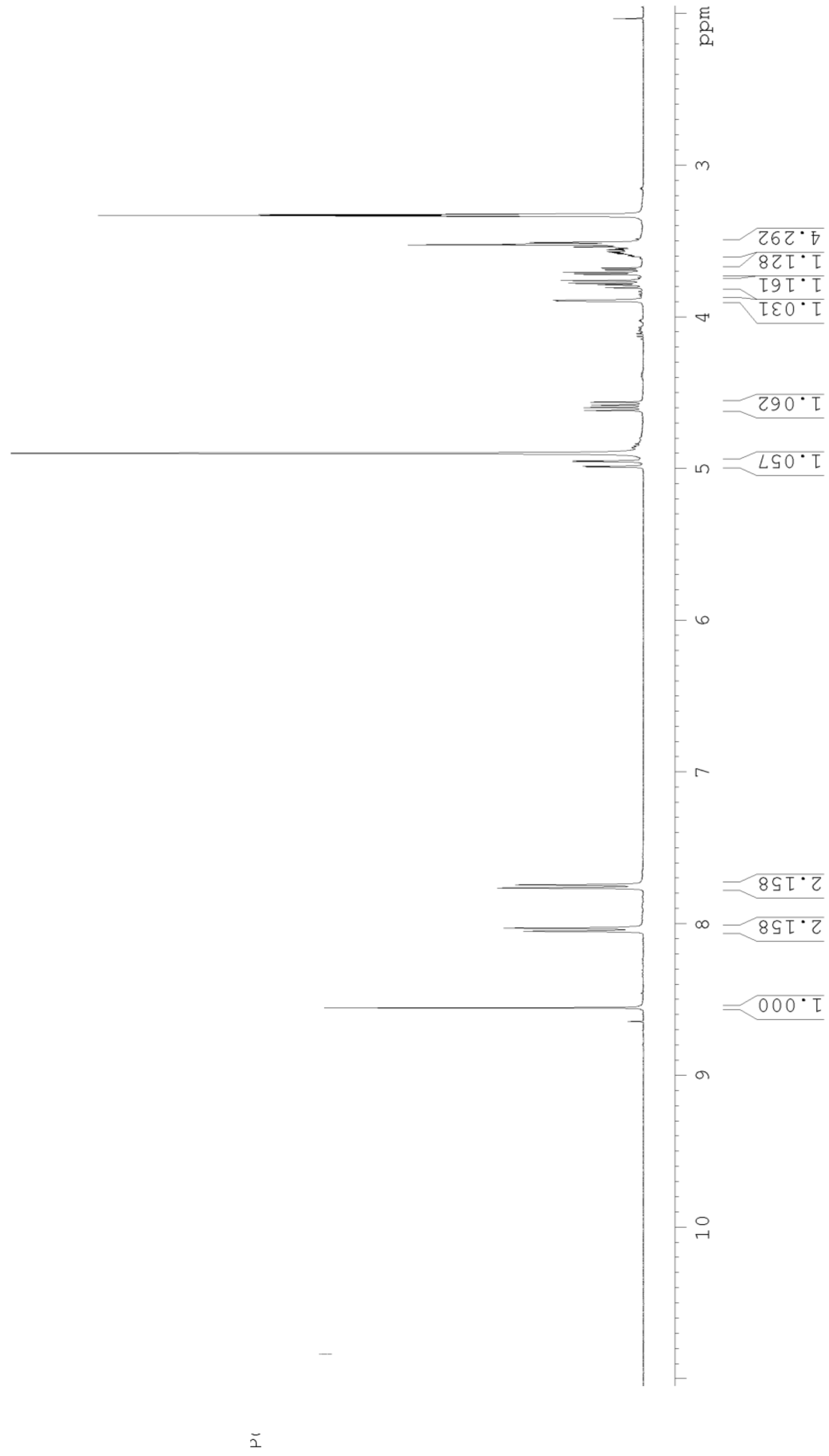

^{13}C:

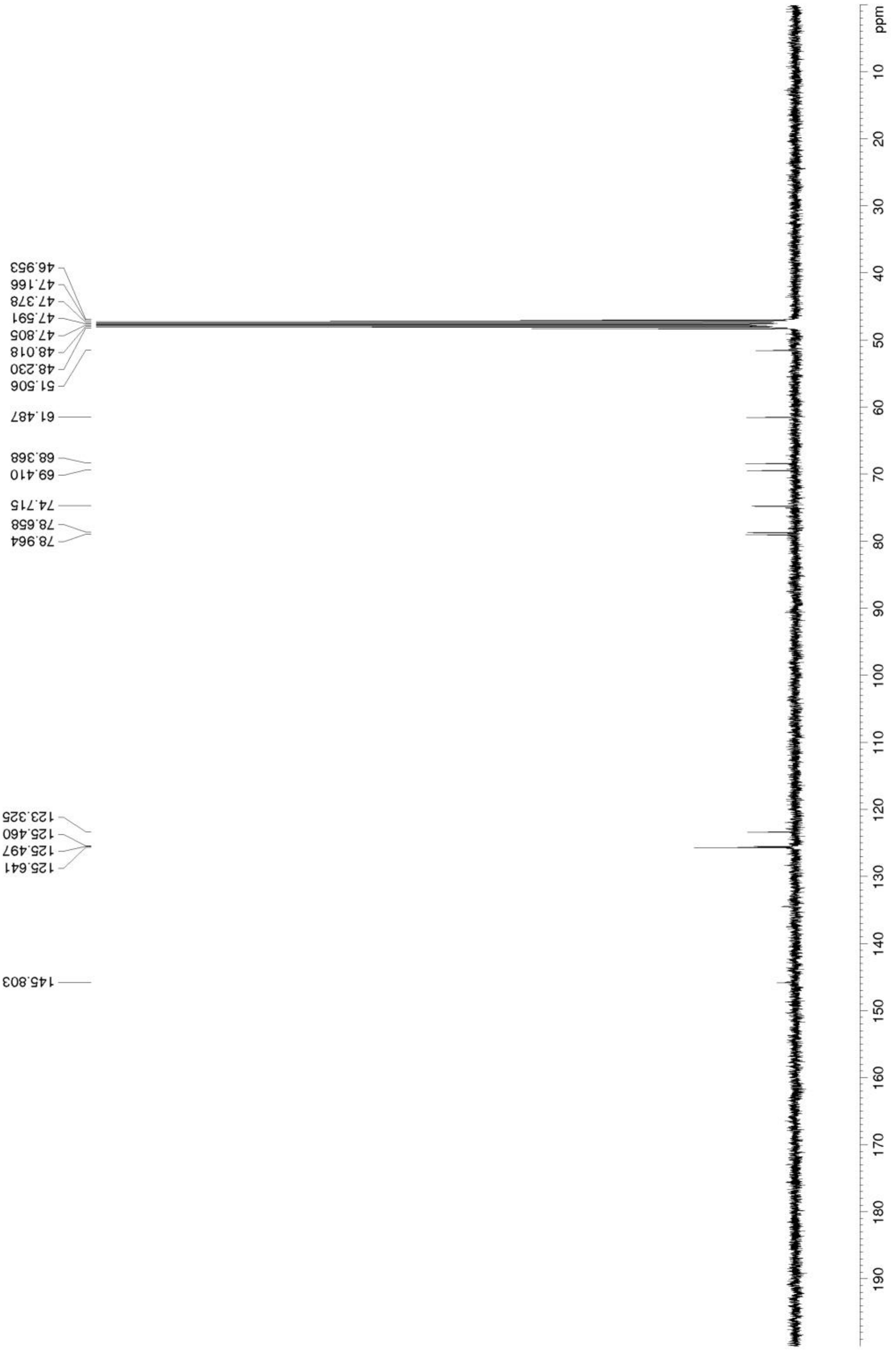

46.953
47.166
47.378
47.591
47.805
48.018
48.230
51.506
61.487
68.368
69.410
74.715
78.658
78.964
123.325
125.460
125.497
125.641
145.803
ppm
10
20
30
40
50
60
70
80
90
100
110
120
130
140
150
160
170
180
190

Compound **1l**:

^{1}H:

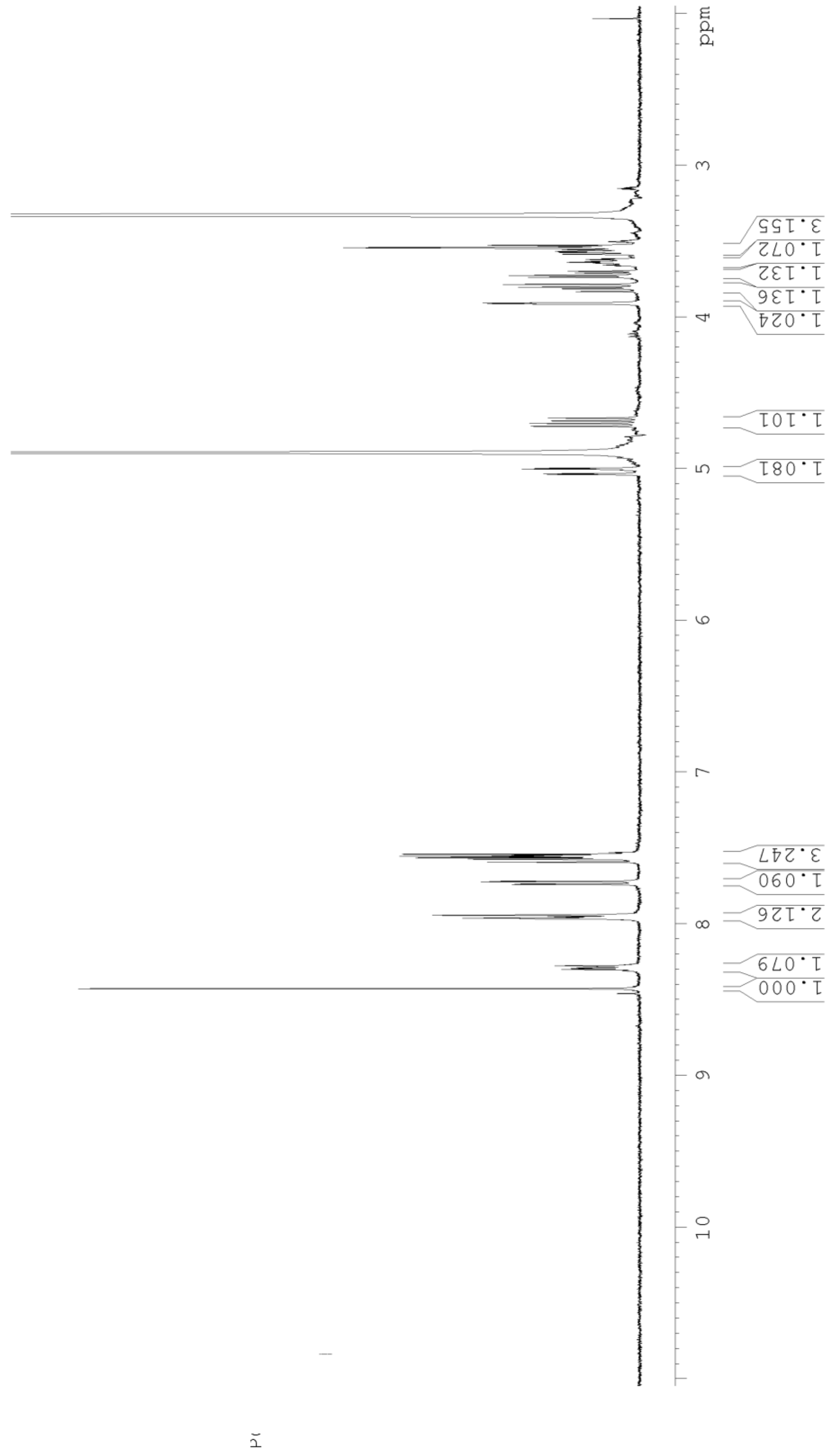

^{13}C:

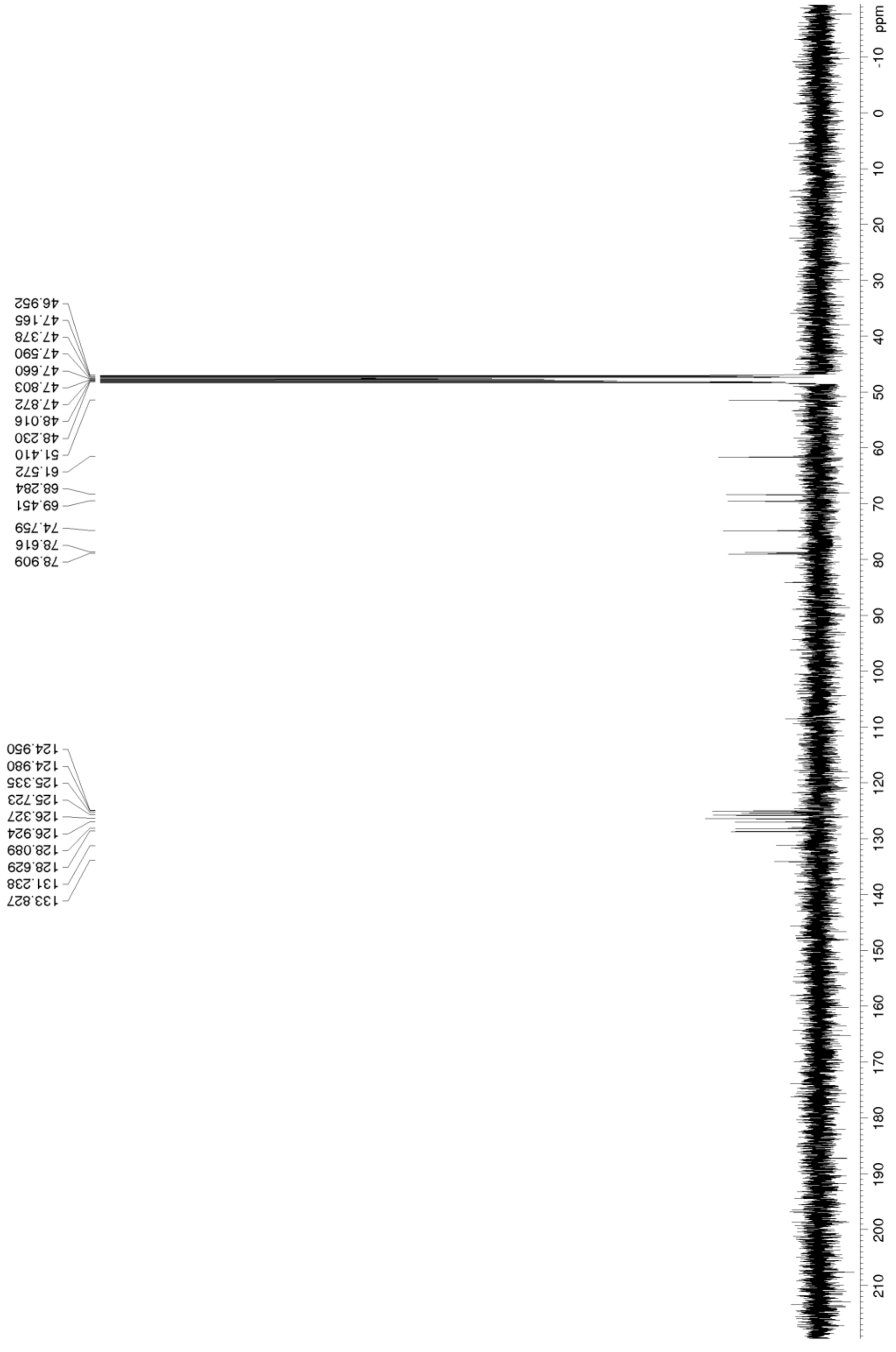
46.952
47.165
47.378
47.590
47.660
47.803
47.872
48.016
48.230
51.410
61.572
68.284
69.451
74.759
78.616
78.909
124.950
124.980
125.335
125.723
126.327
126.924
128.089
128.629
131.238
133.827
ppm
-10
0
10
20
30
40
50
60
70
80
90
100
110
120
130
140
150
160
170
180
190
200
210

Compound **1m**:

^{1}H:

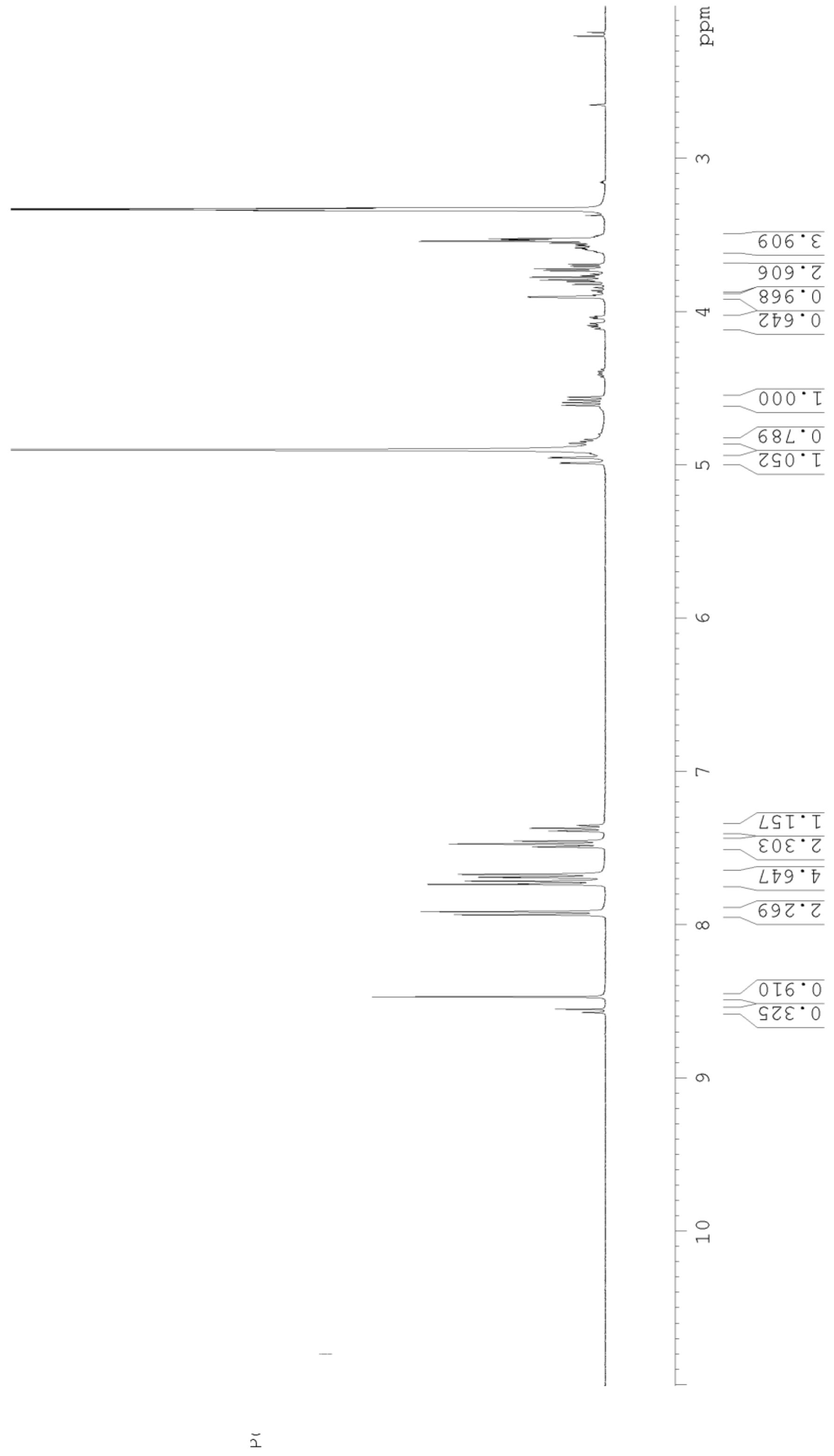

^{13}C:

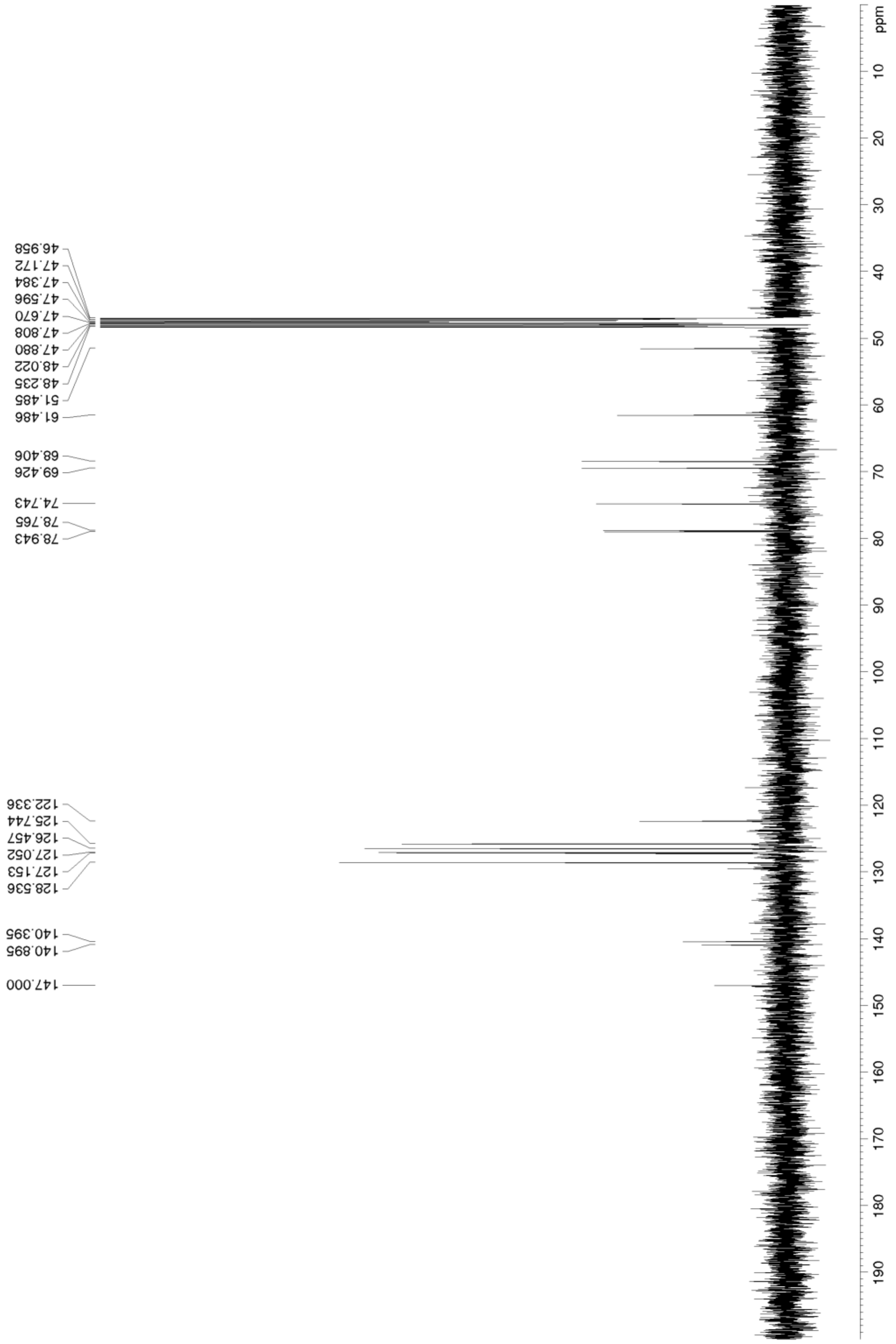

46.958
47.172
47.384
47.596
47.670
47.808
47.880
48.022
48.235
51.485
61.486
68.406
69.426
74.743
78.765
78.943
122.336
125.744
126.457
127.052
127.153
128.536
140.395
140.895
147.000
ppm

Compound **1n**:

^{1}H:

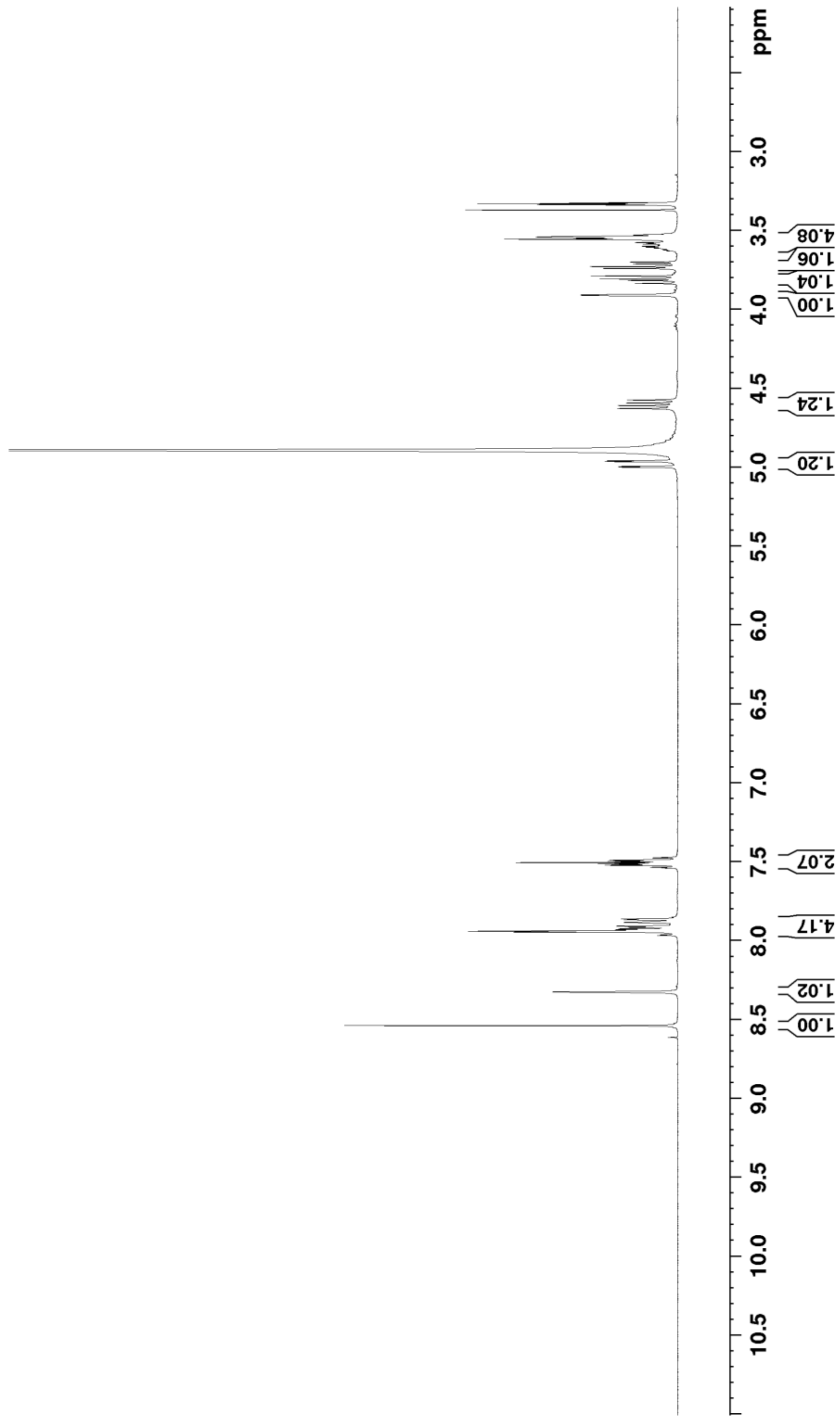

^{13}C:

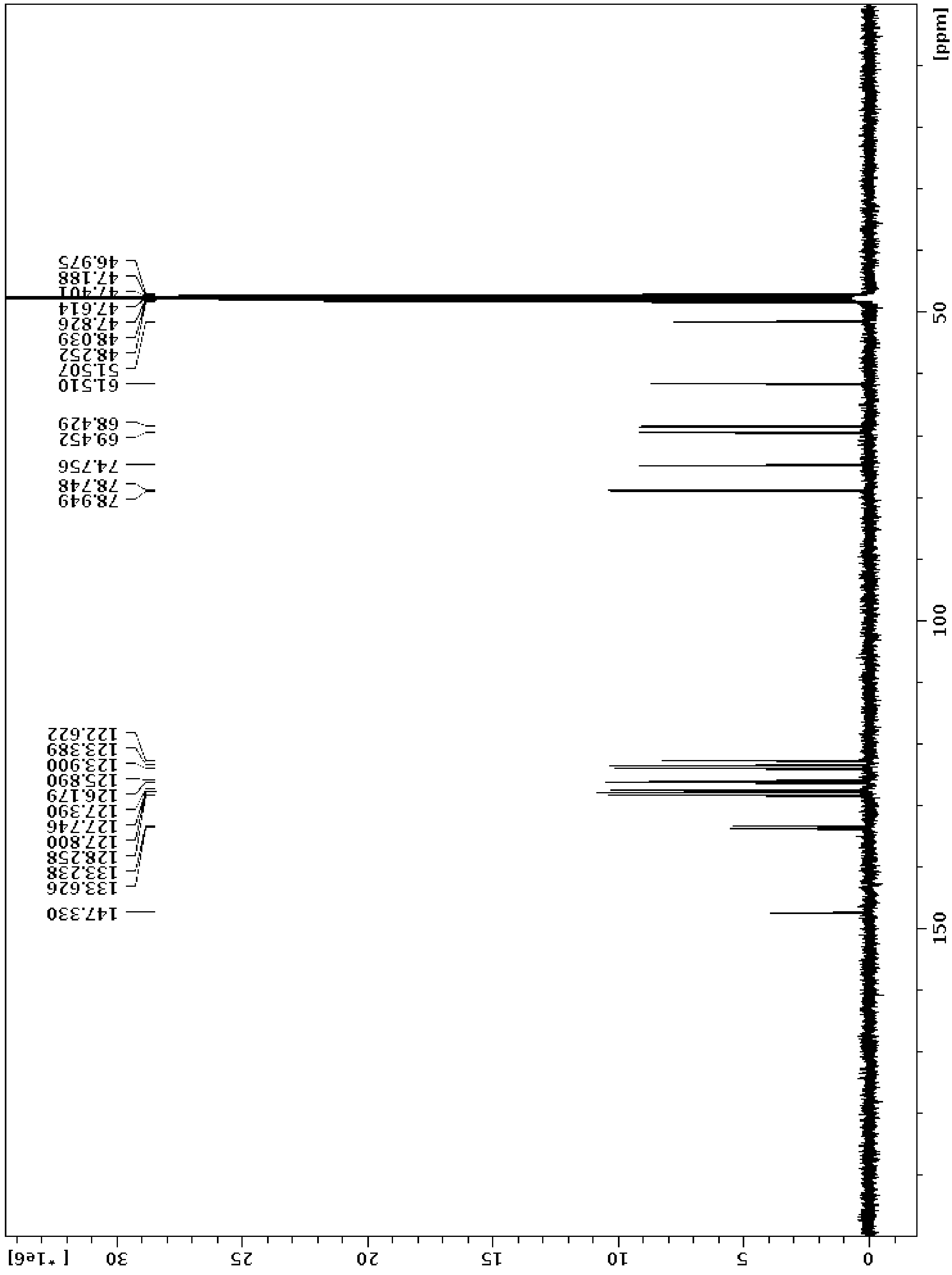
46.975
47.188
47.401
47.614
47.826
48.039
48.252
51.507
61.510
68.429
69.452
74.756
78.748
78.949
122.622
123.389
123.900
125.890
126.179
127.390
127.746
127.800
128.258
133.238
133.626
147.330
[ppm]
50
100
150
[*1e6]
30
25
20
15
10
5
0

Compound **3**:

^{1}H:

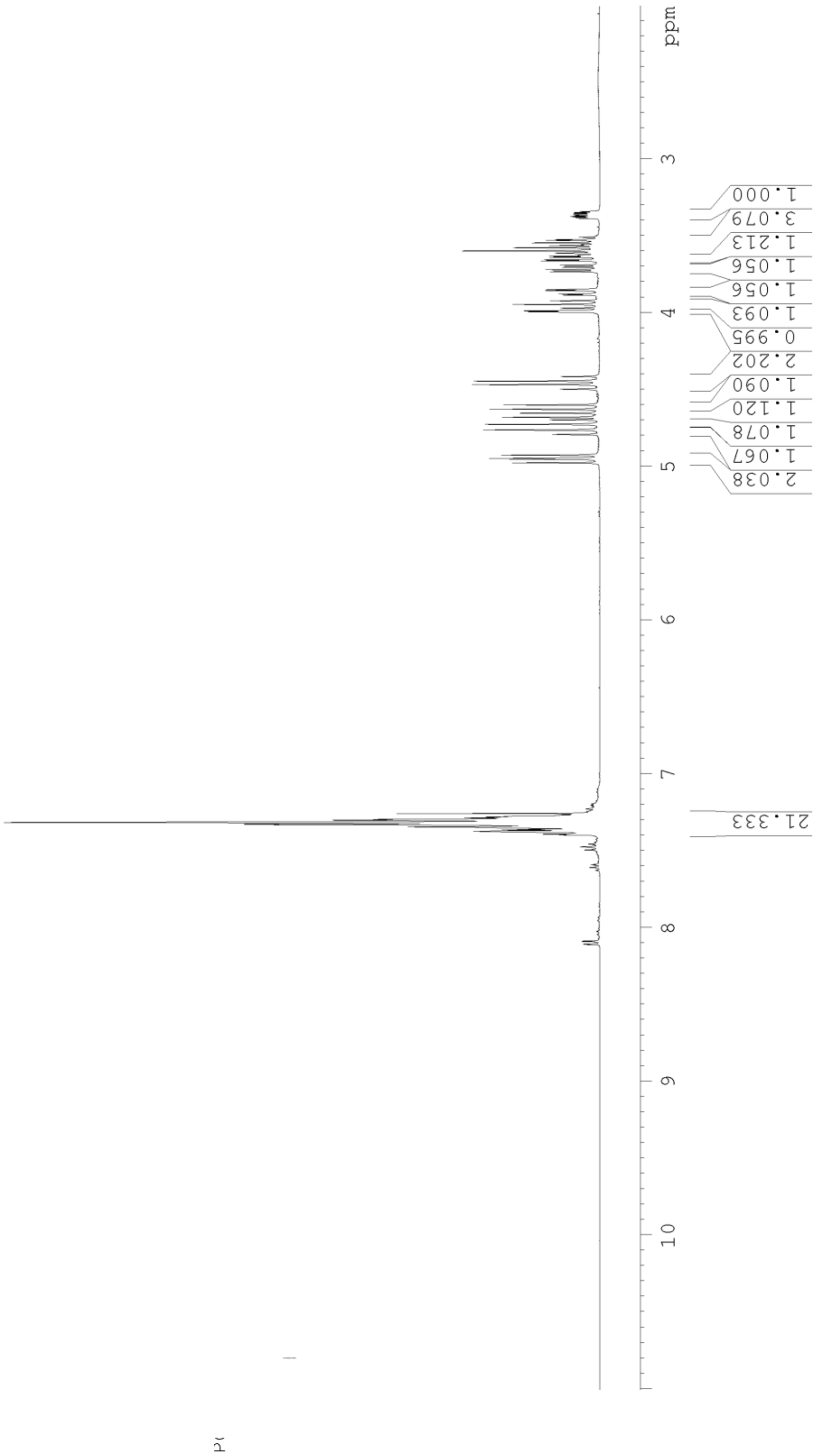

^{13}C:

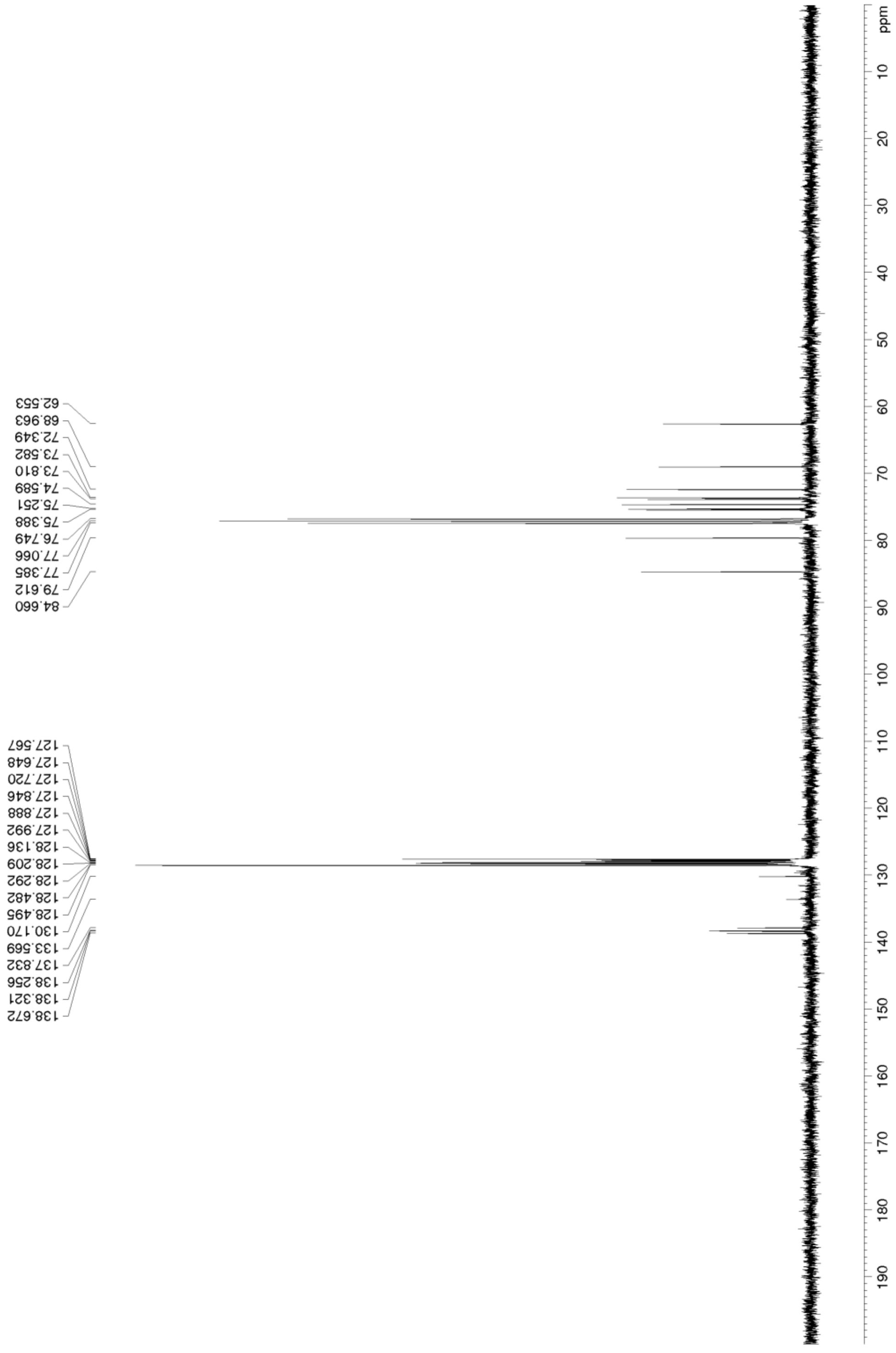
62.553
68.963
72.349
73.582
73.810
74.589
75.251
75.388
76.749
77.066
77.385
79.612
84.660
127.567
127.648
127.720
127.846
127.888
127.992
128.136
128.209
128.292
128.482
128.495
130.170
133.569
137.832
138.256
138.321
138.672
ppm

Compound **3** continued:

[1]H pyridine-d5:

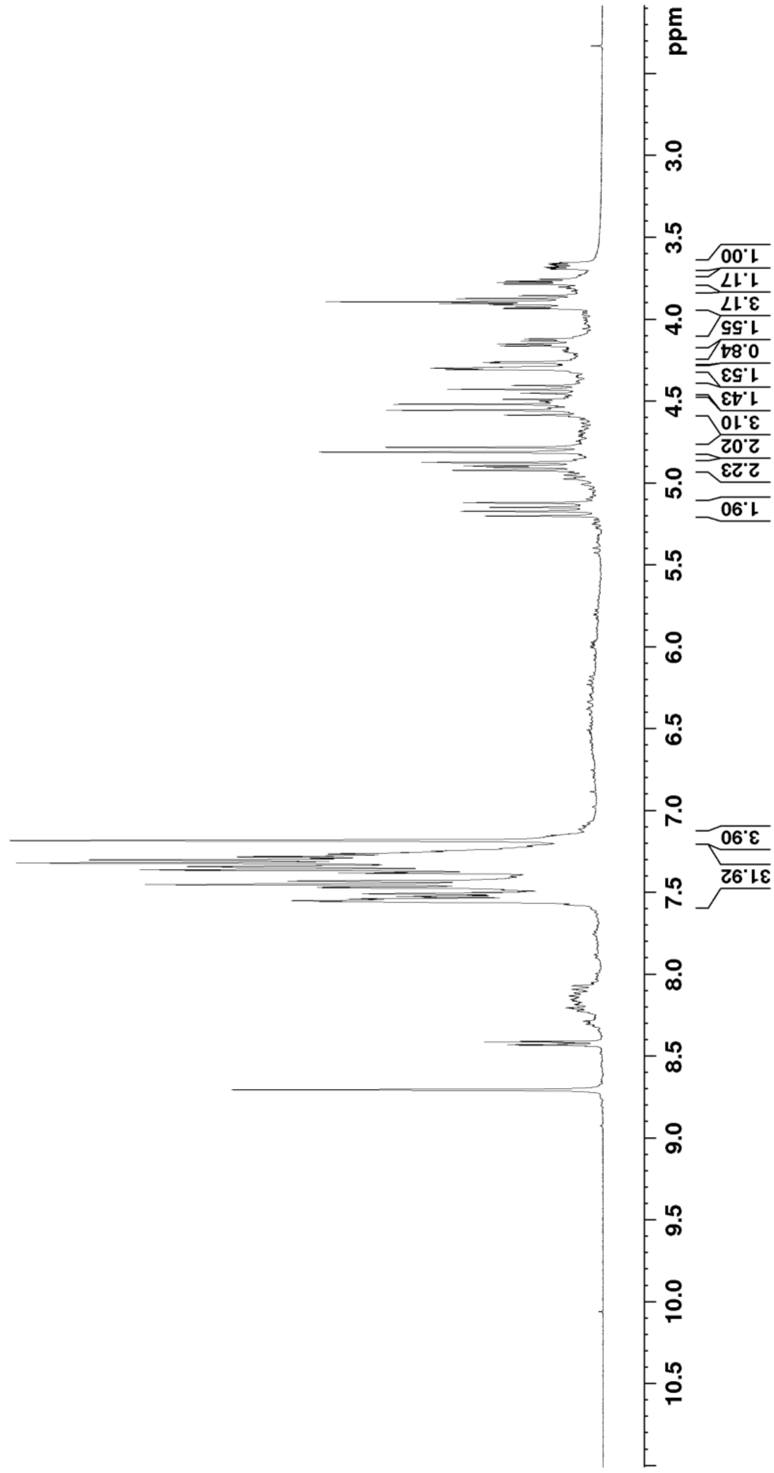

NOESY pyridine-d5:

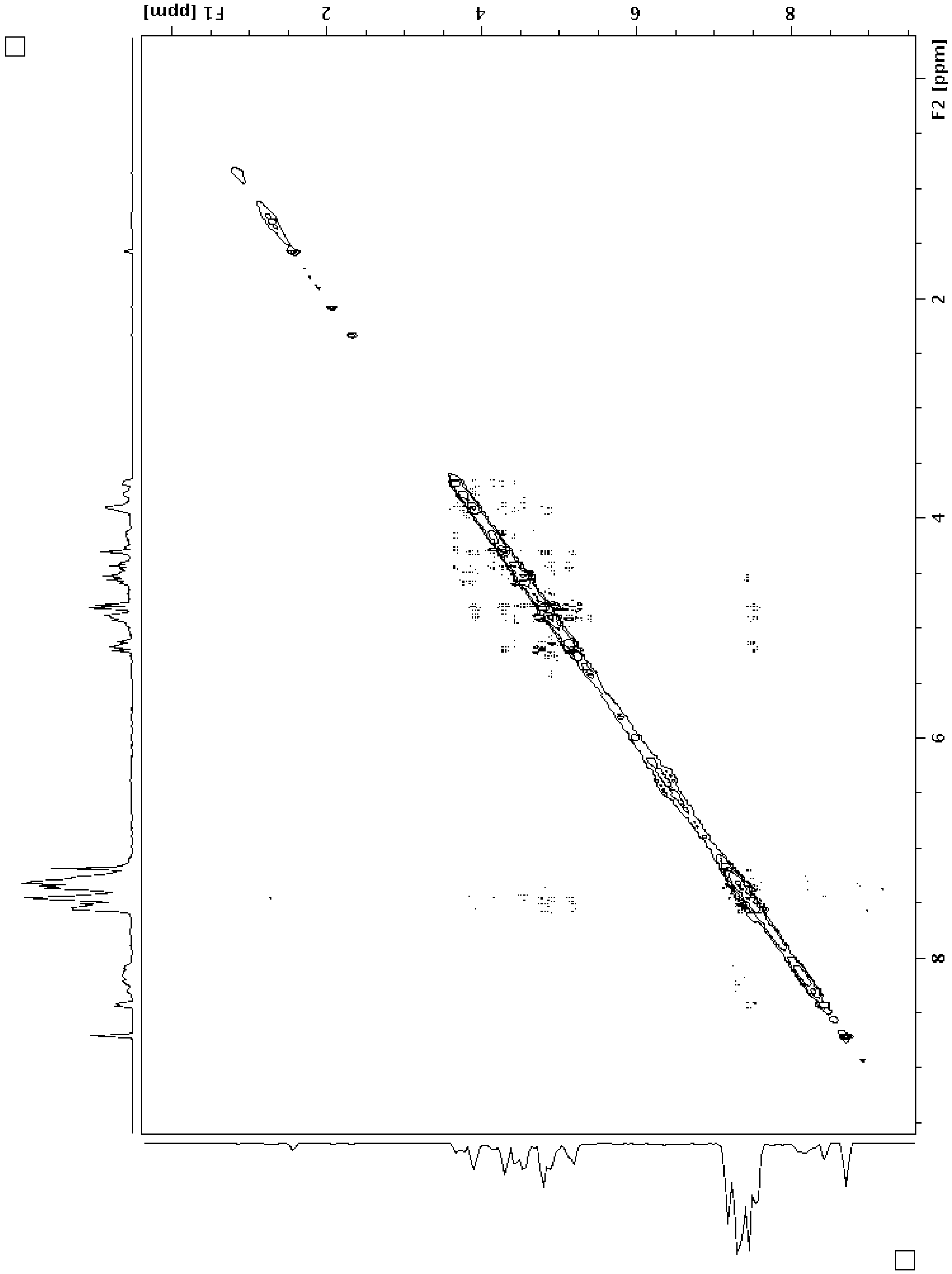

Compound **5**:

^{1}H:

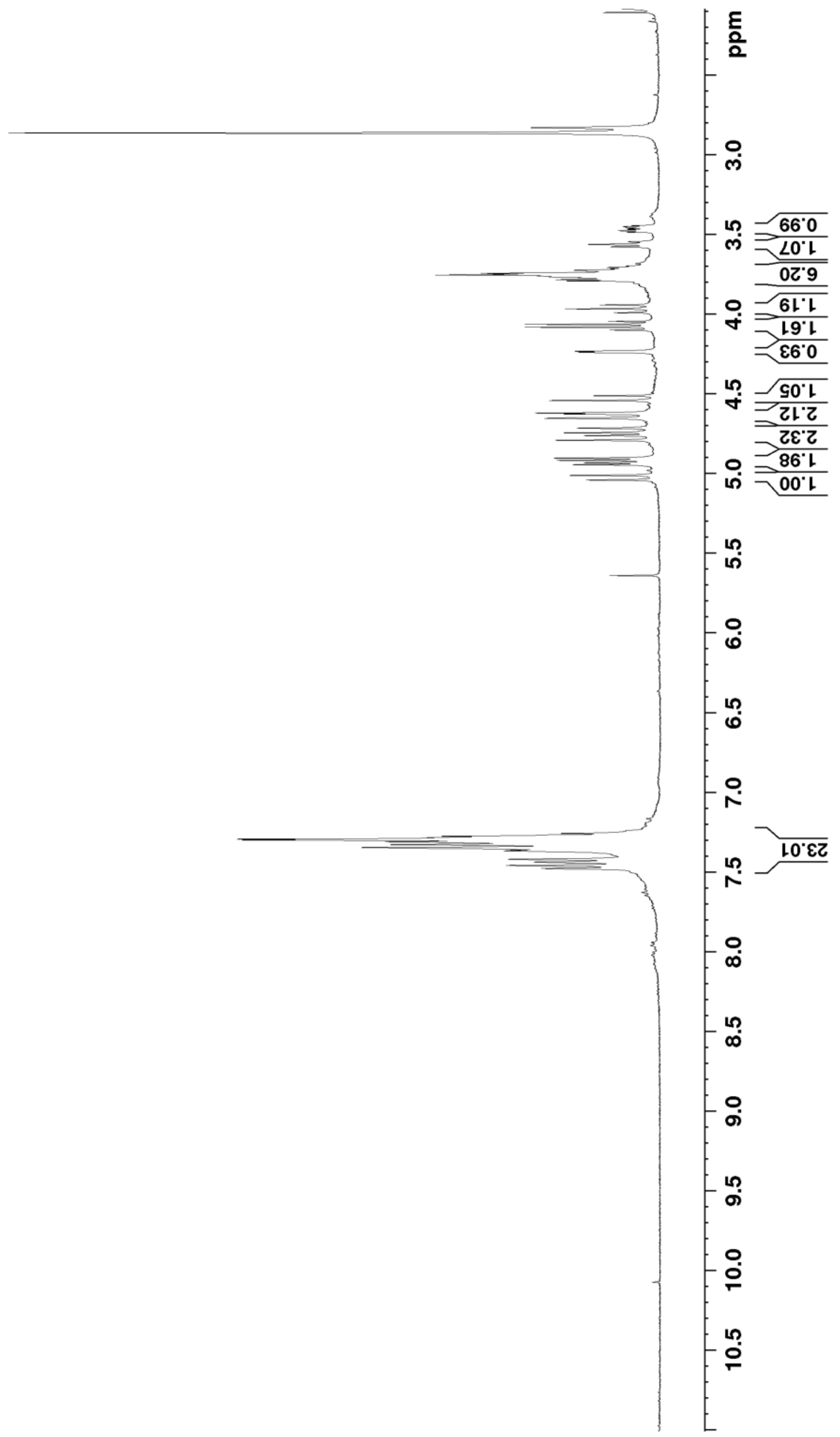

[13]C:

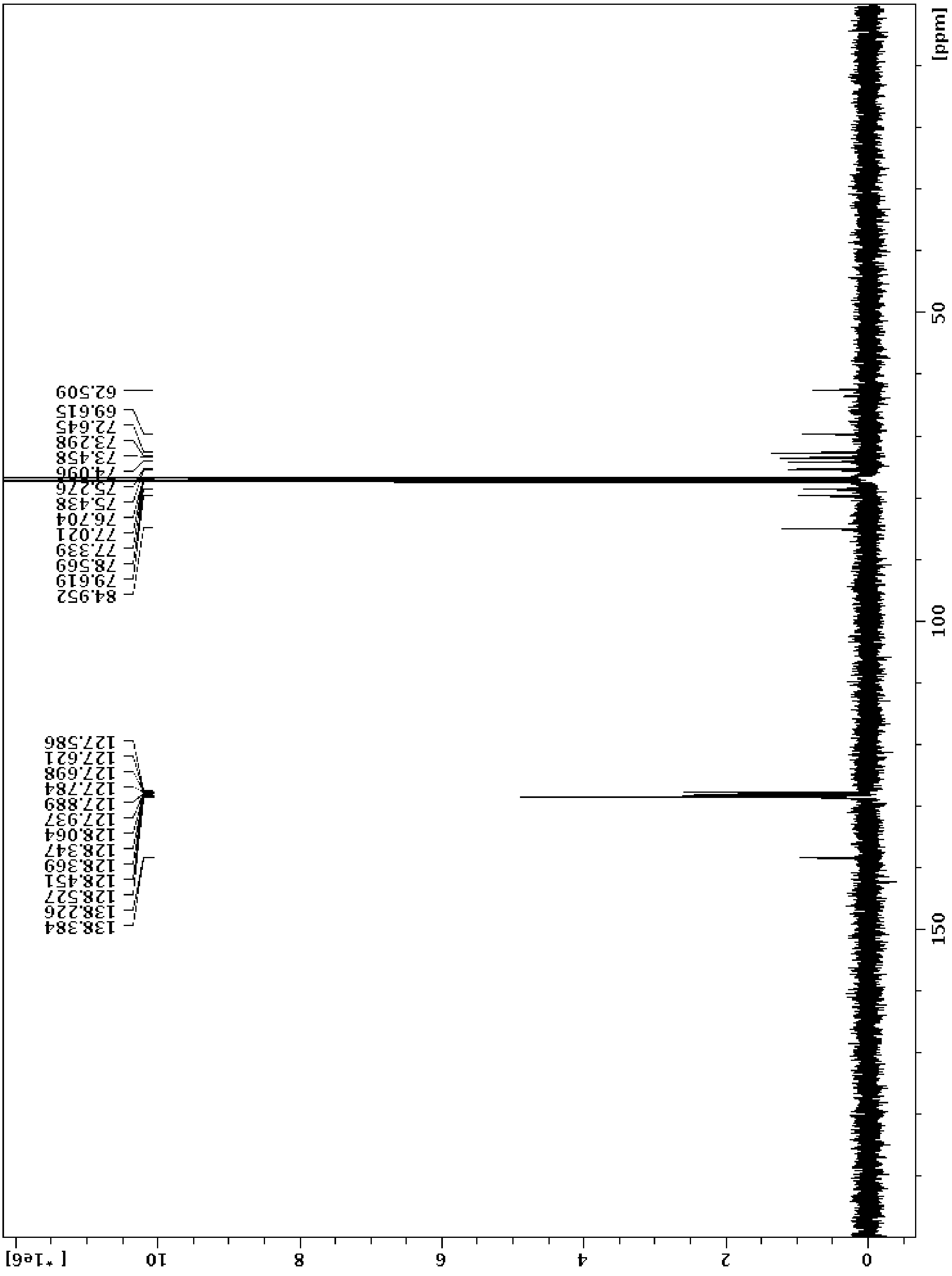

62.509
69.615
72.645
73.298
73.458
74.096
75.276
75.438
76.704
77.021
77.339
78.569
79.619
84.952
127.586
127.621
127.698
127.784
127.889
127.937
128.064
128.347
128.369
128.451
128.527
138.226
138.384
[ppm]
50
100
150
[*1e6]
10
8
6
4
2
0

Compound **5** continued:

^{1}H pyridine-d5:

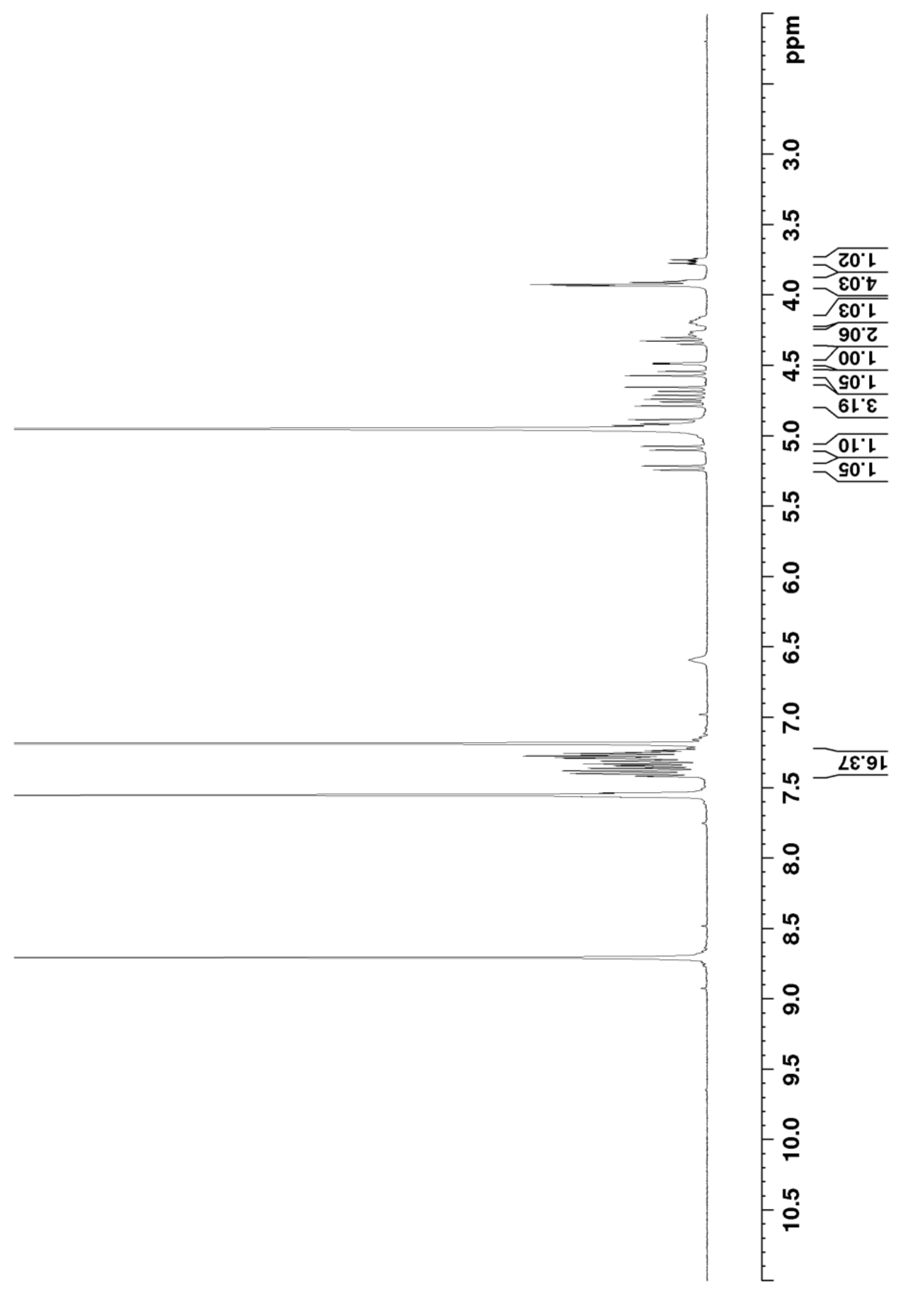

NOESY pyridine-d5:

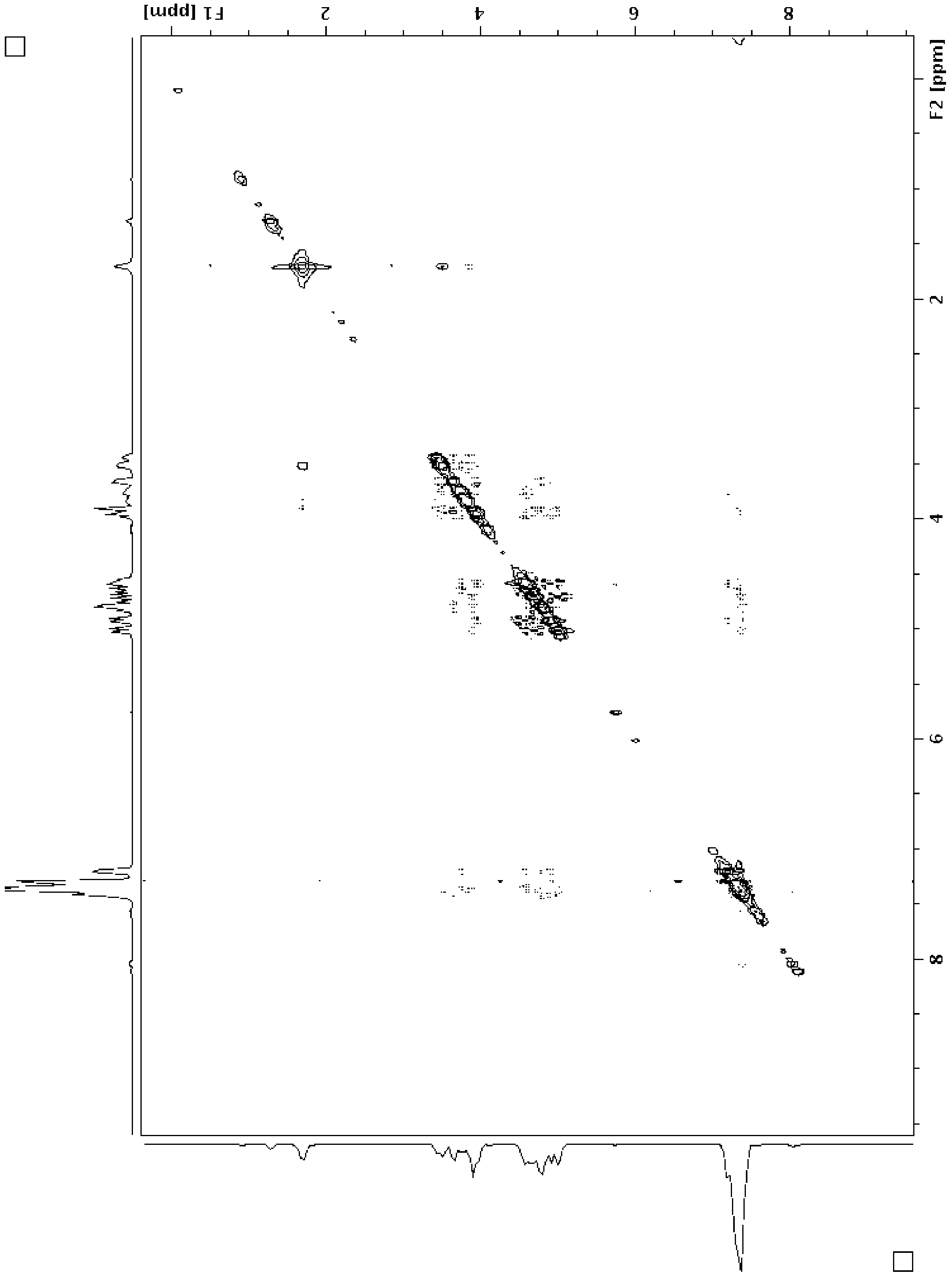

Compound β-**7**
^{1}H:

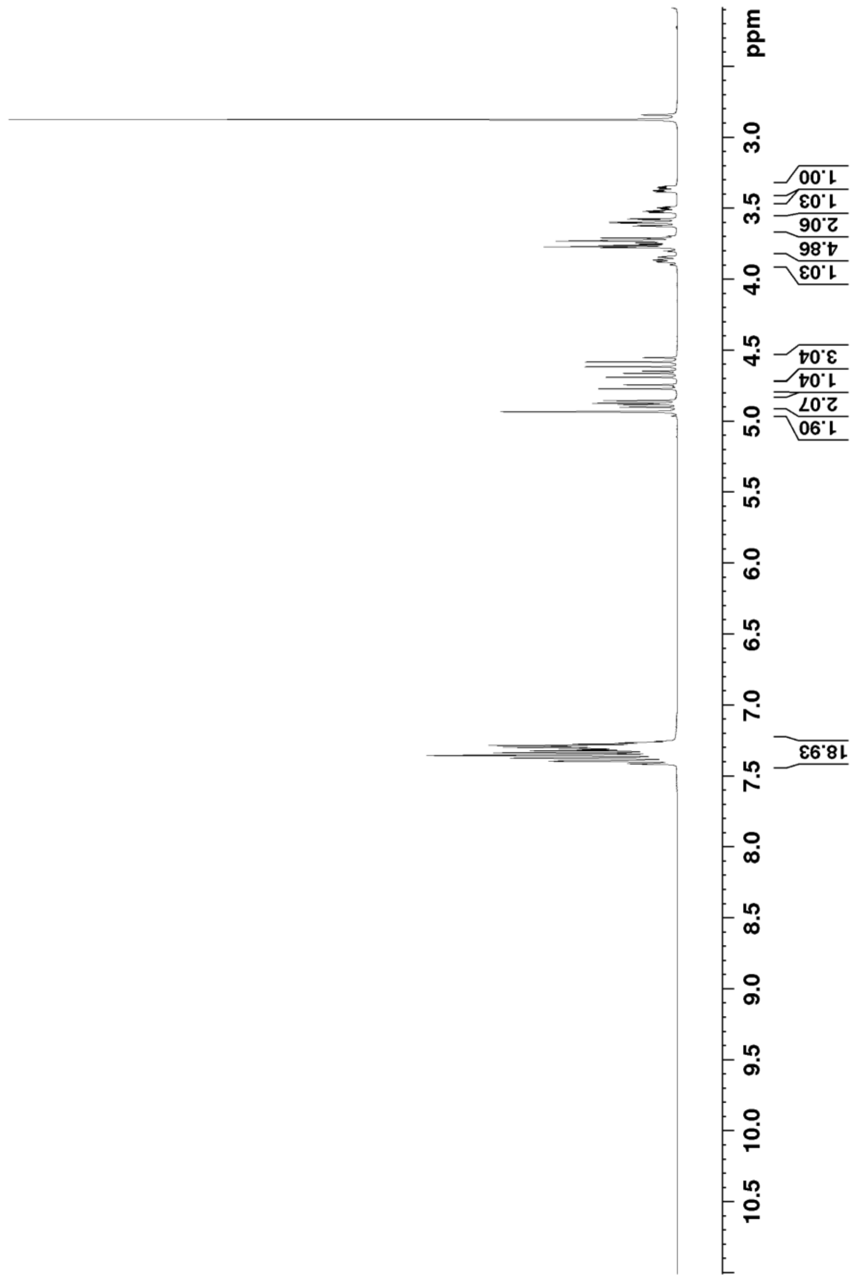

^{13}C:

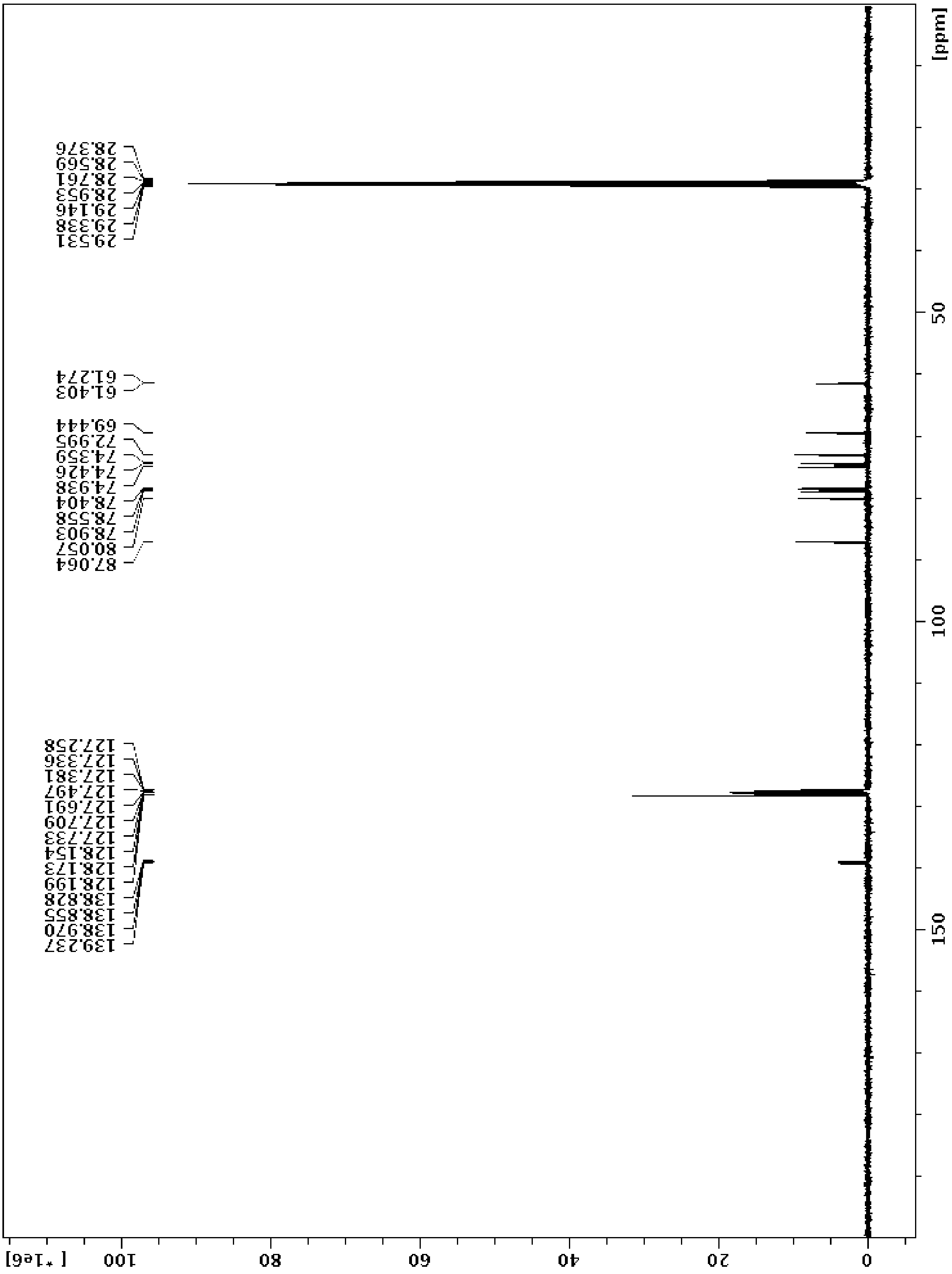

28.376
28.569
28.761
28.953
29.146
29.338
29.531
61.274
61.403
69.444
72.995
74.359
74.426
74.938
78.404
78.558
78.903
80.057
87.064
127.258
127.336
127.381
127.497
127.691
127.709
127.733
128.154
128.173
128.199
138.828
138.855
138.970
139.237
[ppm]
50
100
150
100
80
60
40
20
0
[*1e6]

Compound β-**7**

^{1}H pyridine-d5

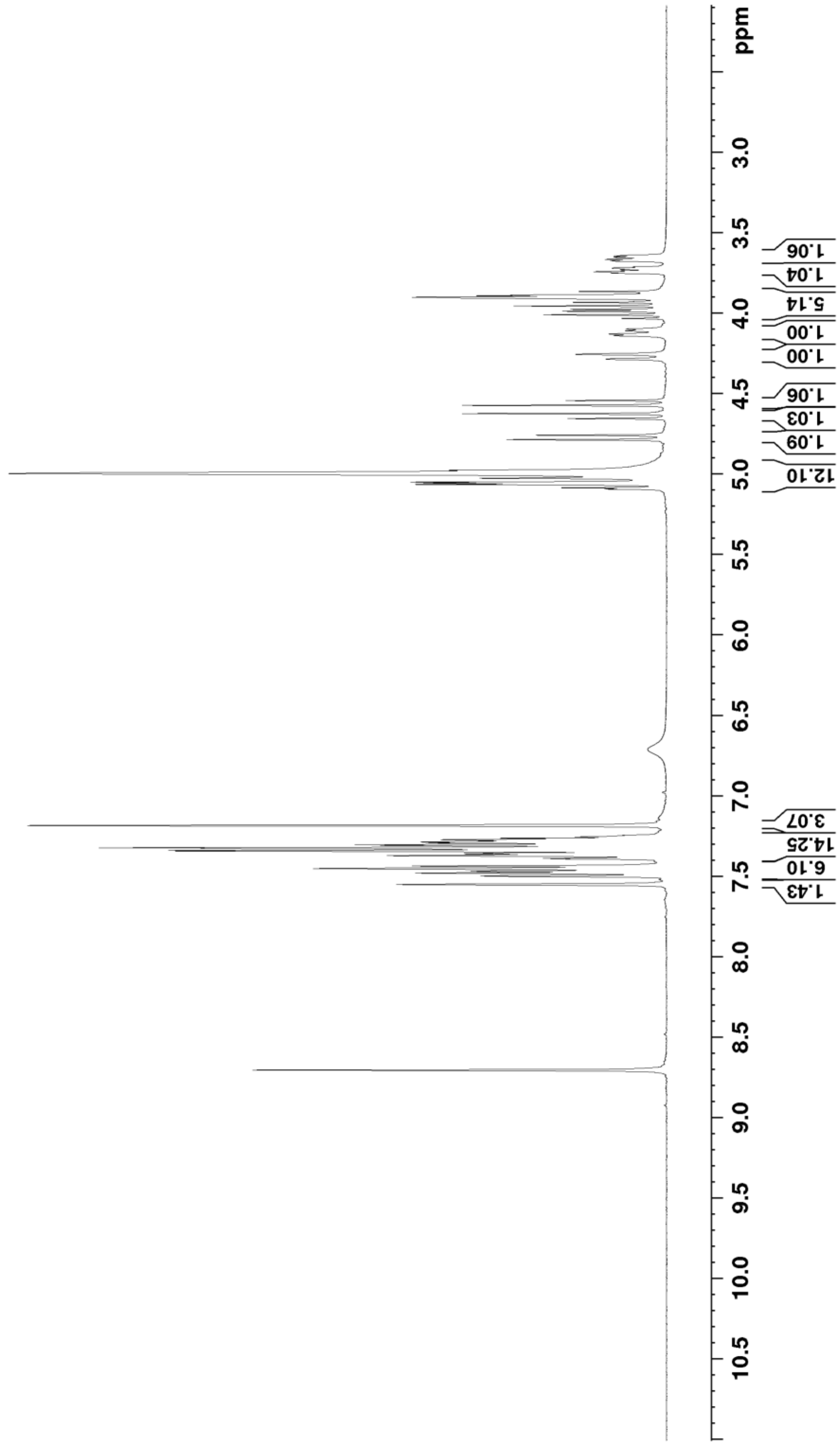

NOESY pyridine-d5:

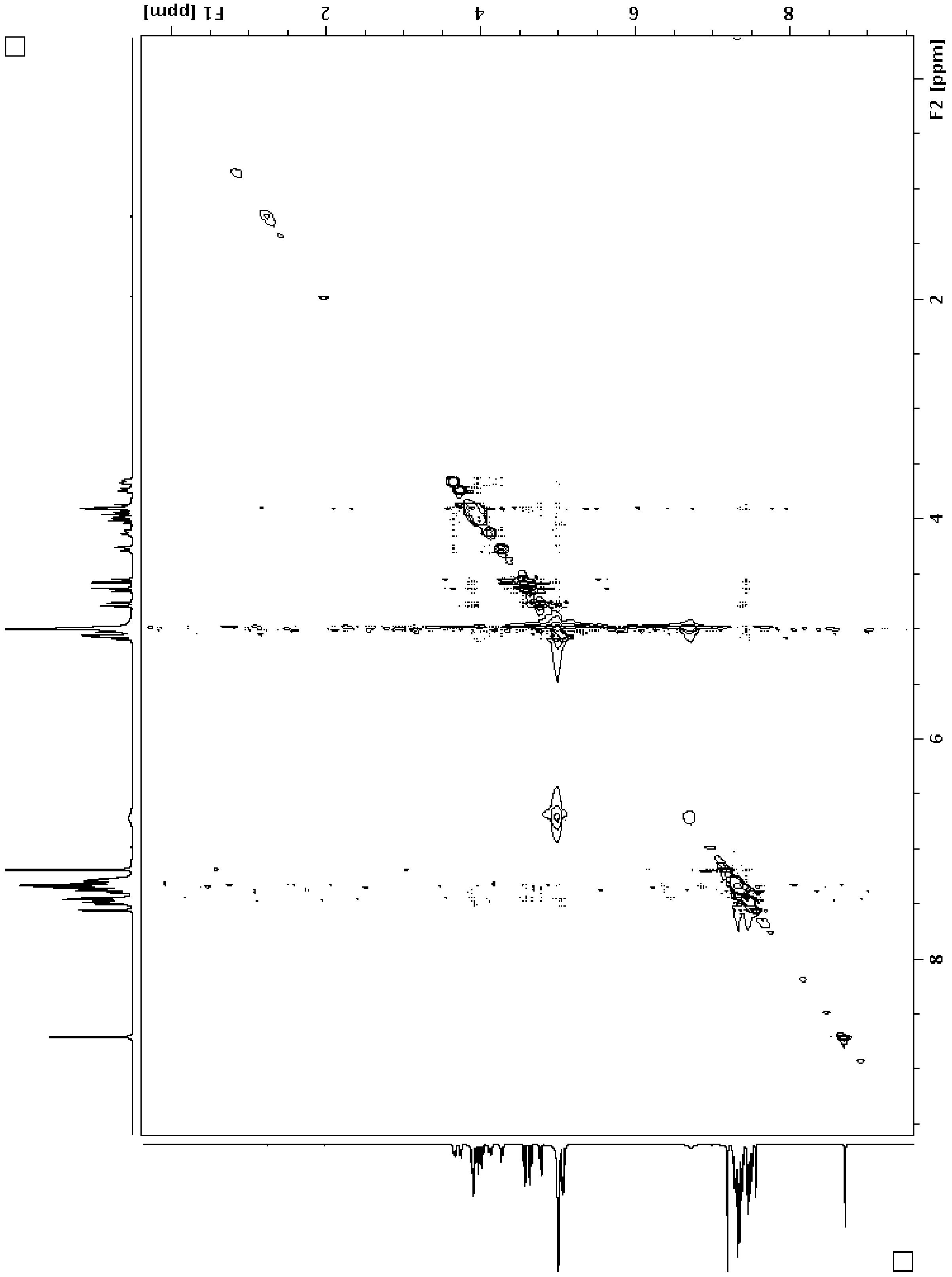

Compound α-**7**
^{1}H:

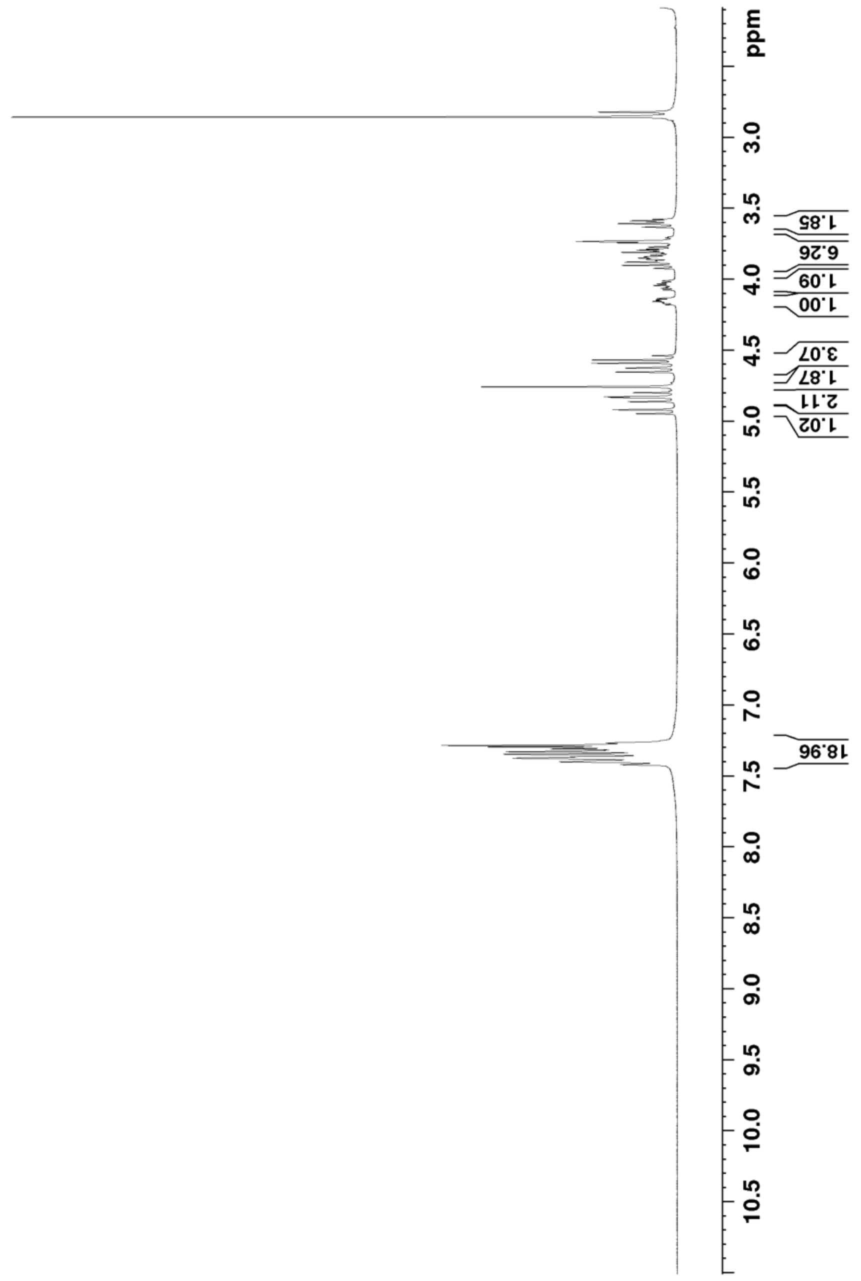

^{13}C:

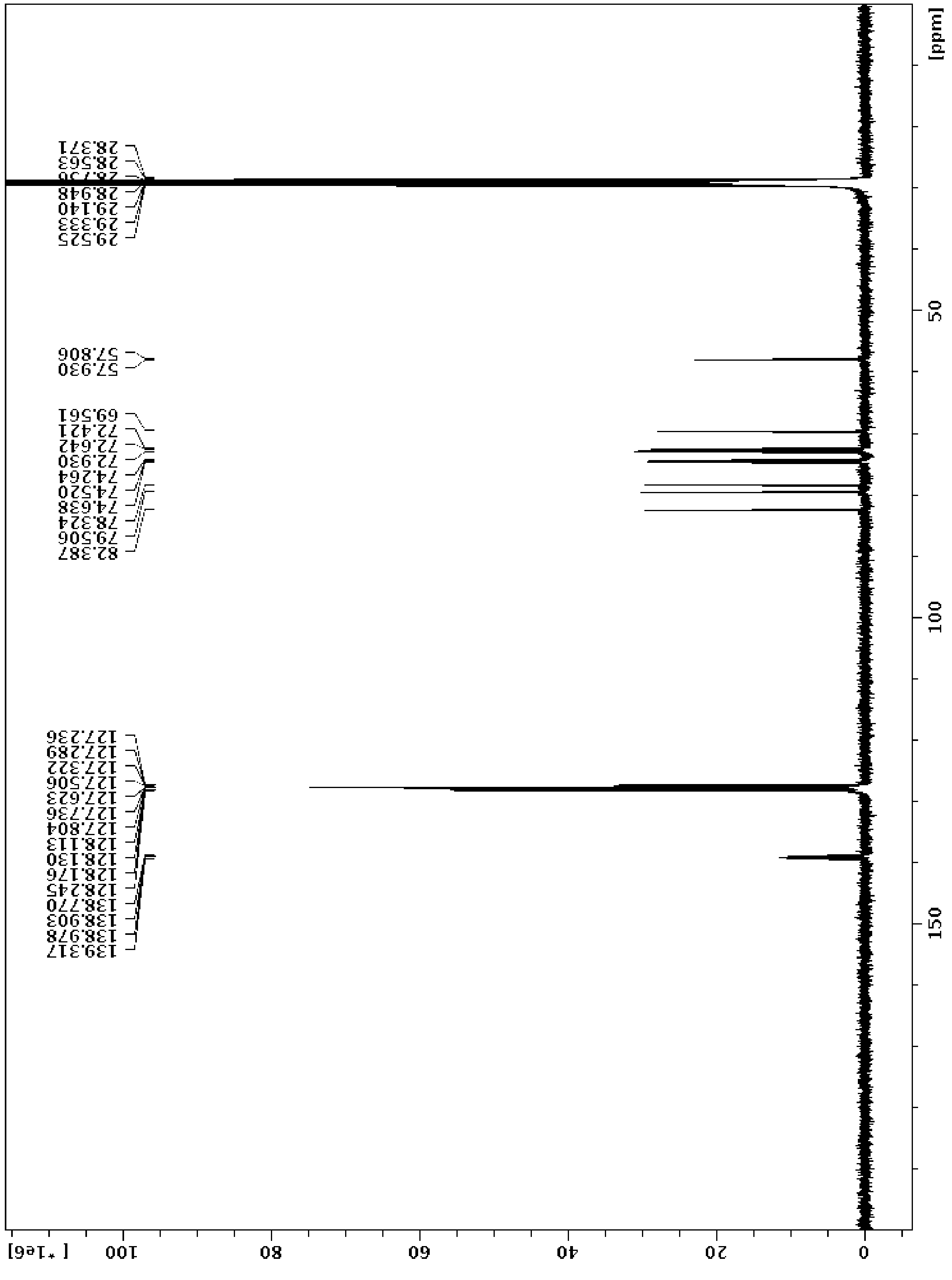

28.371
28.563
28.948
29.140
29.333
29.525
57.806
57.930
69.561
72.421
72.642
72.930
74.264
74.520
74.638
78.324
79.506
82.387
127.236
127.289
127.322
127.506
127.623
127.736
127.804
128.113
128.130
128.176
128.245
138.770
138.903
138.978
139.317
[ppm]
50
100
150
[*1e6]
100
80
60
40
20
0

Compound β-**7**
[1]H pyridine-d5:

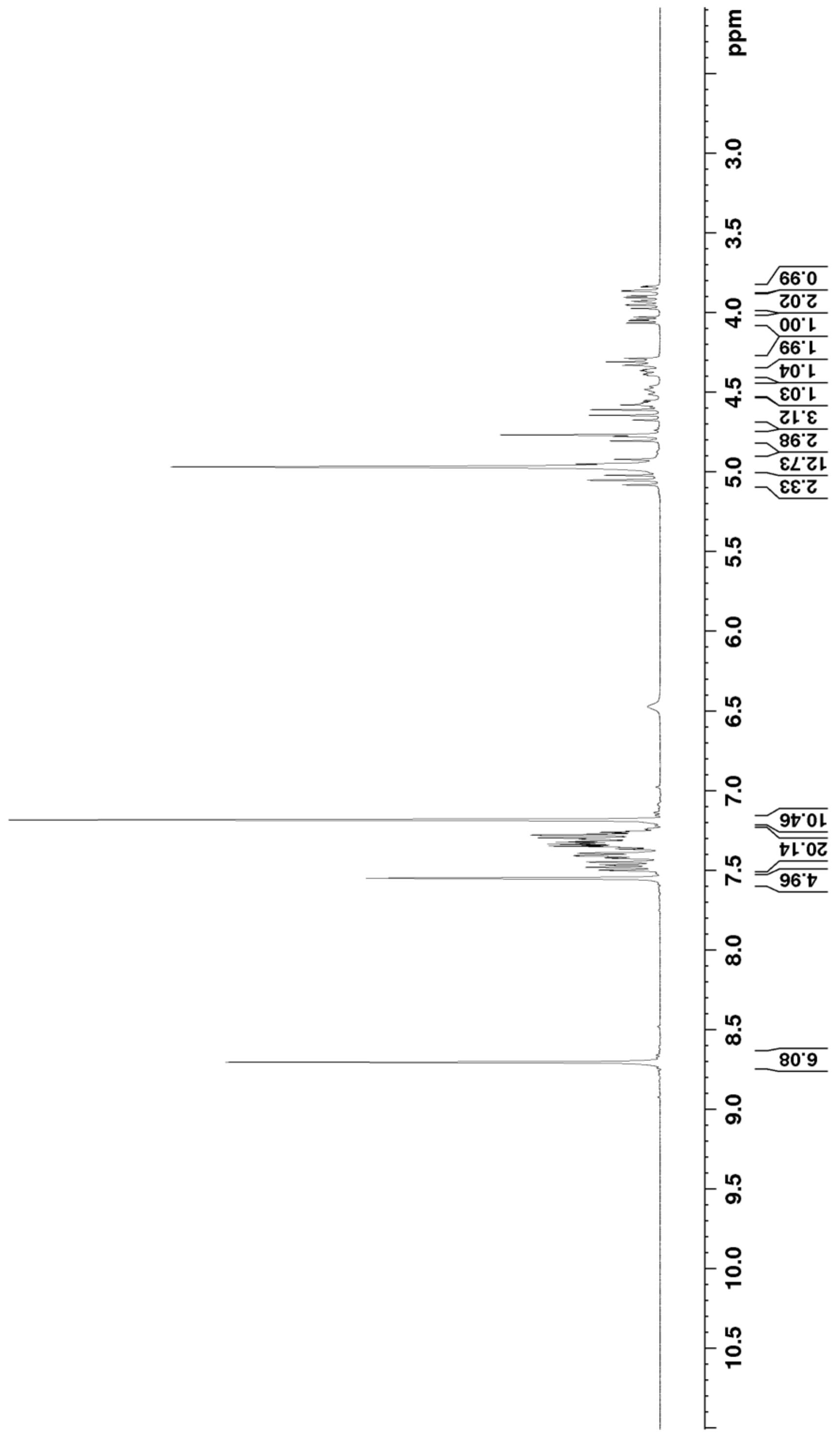

NOESY pyridine-d5:

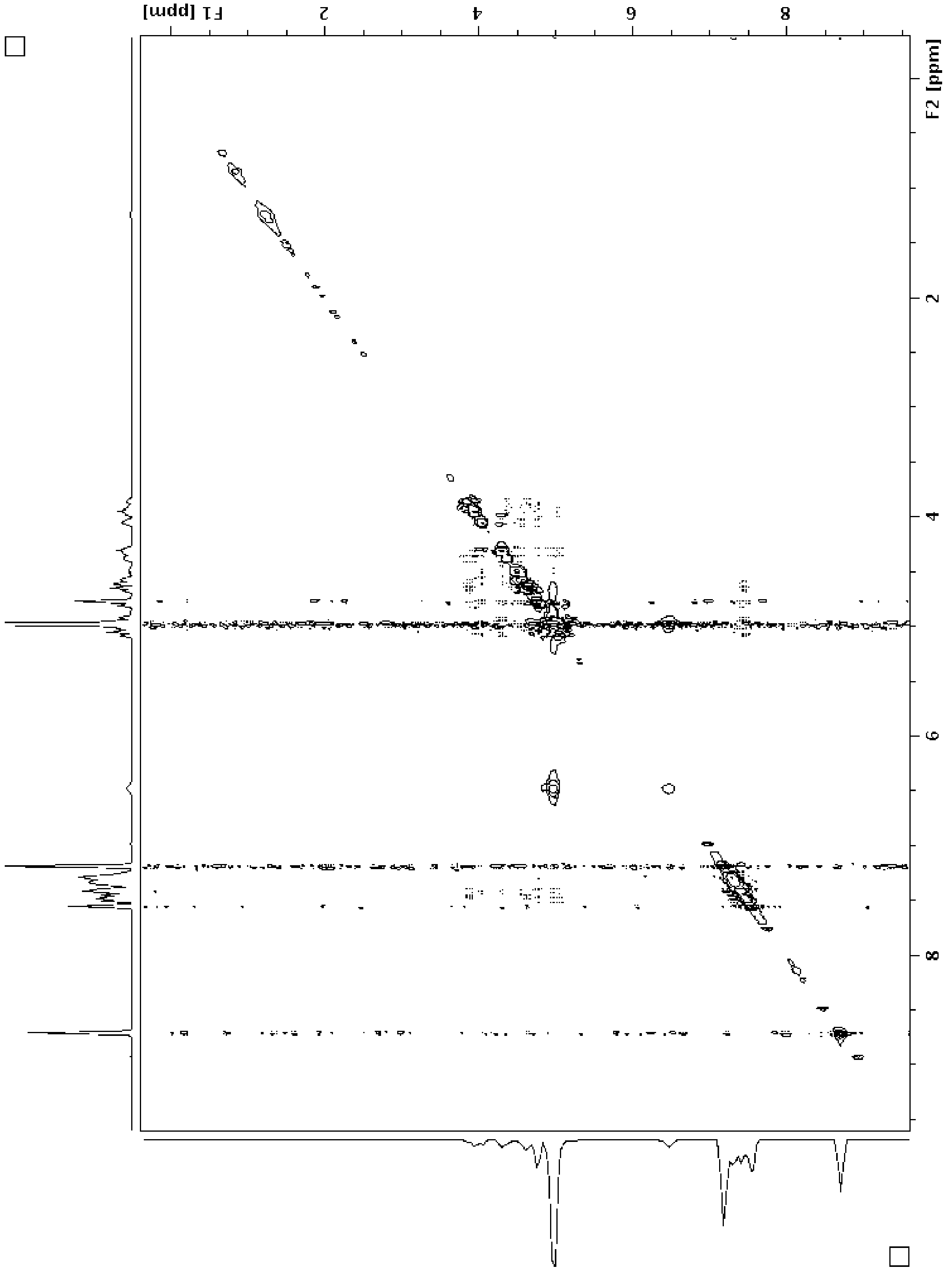

Compound **8**:

^{1}H:

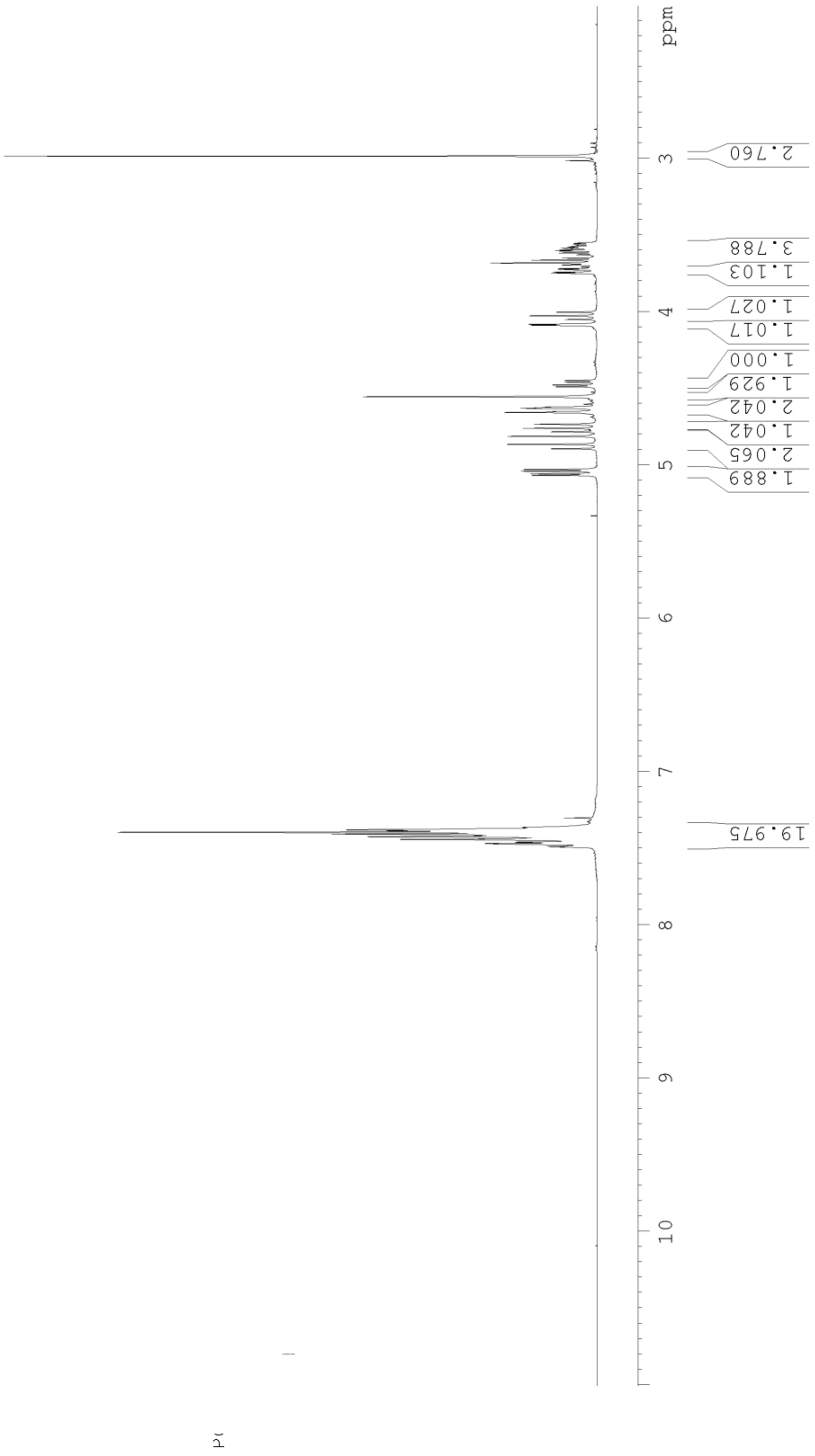

^{13}C:

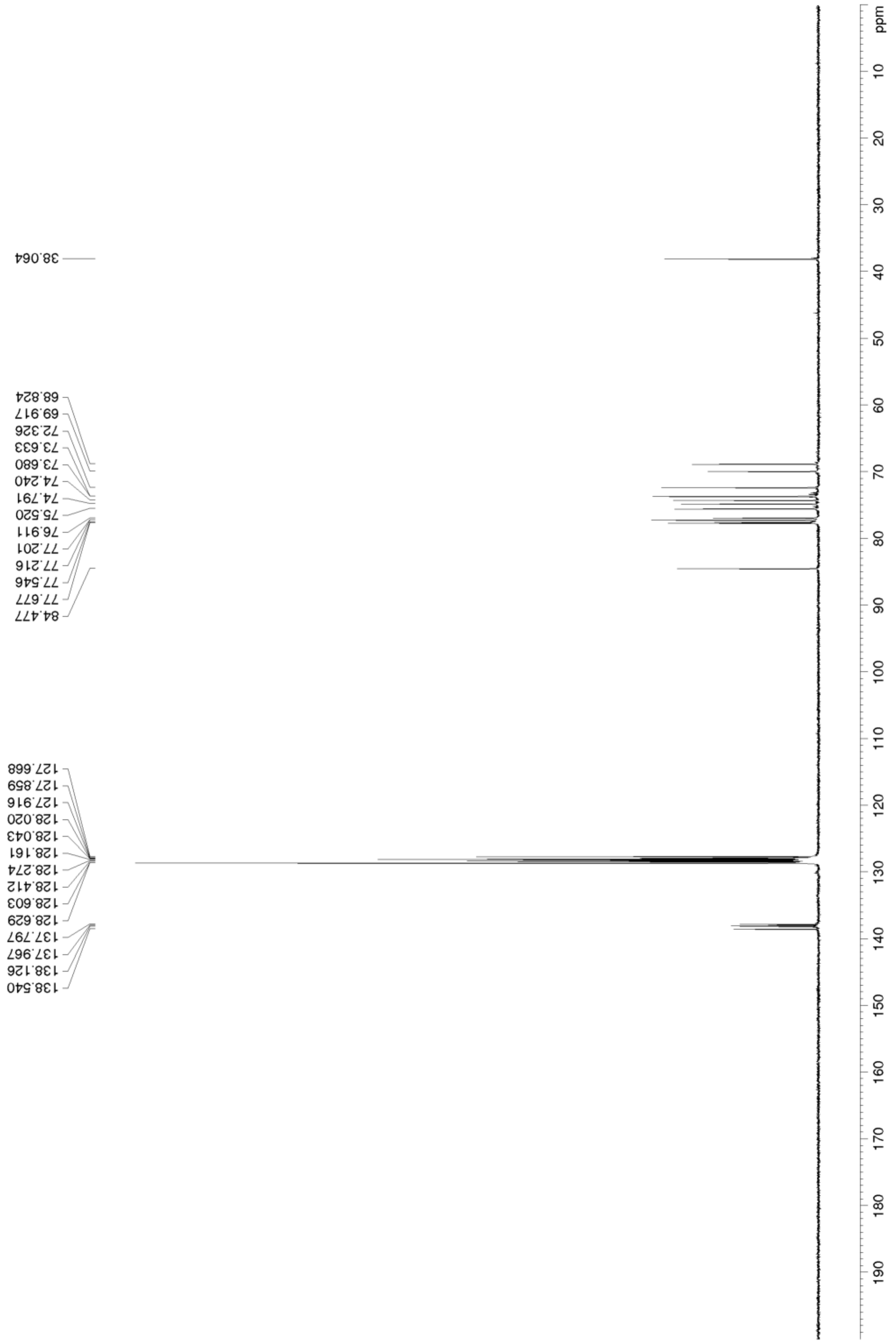
38.064
68.824
69.917
72.326
73.633
73.680
74.240
74.791
75.520
76.911
77.201
77.216
77.546
77.677
84.477
127.668
127.859
127.916
128.020
128.043
128.161
128.274
128.412
128.603
128.629
137.797
137.967
138.126
138.540
ppm
10
20
30
40
50
60
70
80
90
100
110
120
130
140
150
160
170
180
190

Compound **9**:

^{1}H:

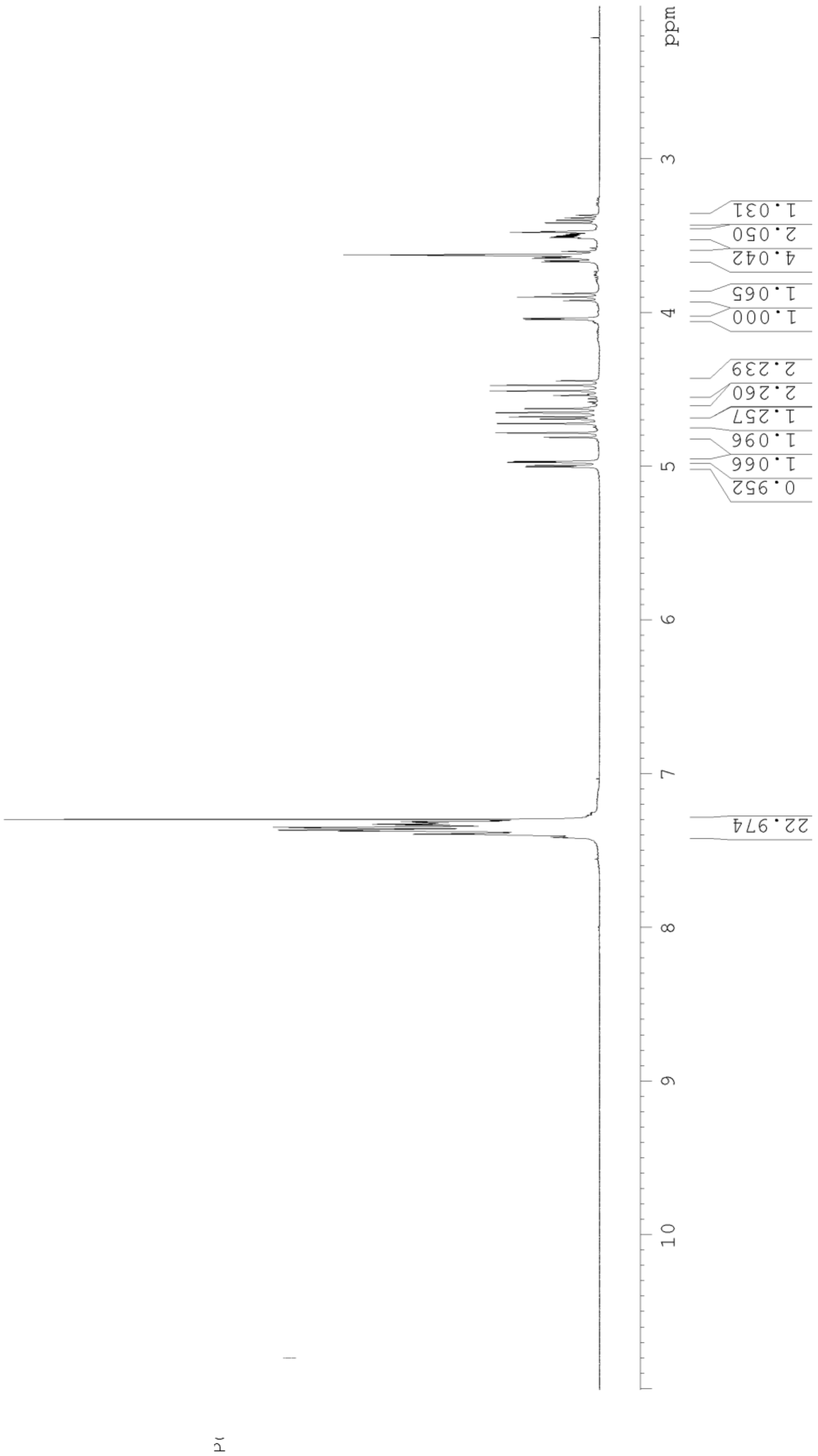

[13]C:

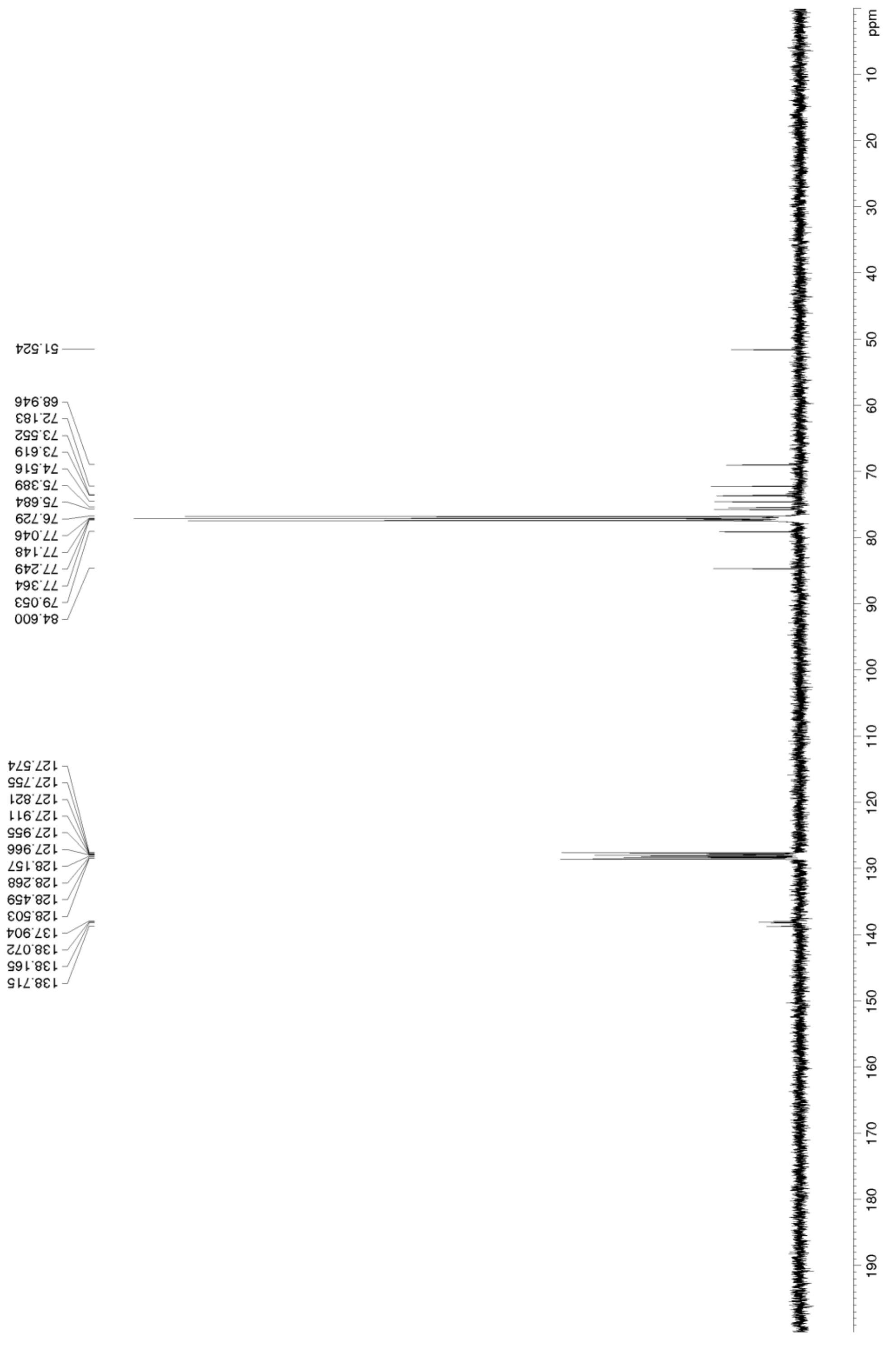
51.524
68.946
72.183
73.552
73.619
74.516
75.389
75.684
76.729
77.046
77.148
77.249
77.364
79.053
84.600
127.574
127.755
127.821
127.911
127.955
127.966
128.157
128.268
128.459
128.503
137.904
138.072
138.165
138.715
ppm
10
20
30
40
50
60
70
80
90
100
110
120
130
140
150
160
170
180
190

Compound **10a**:

^{1}H:

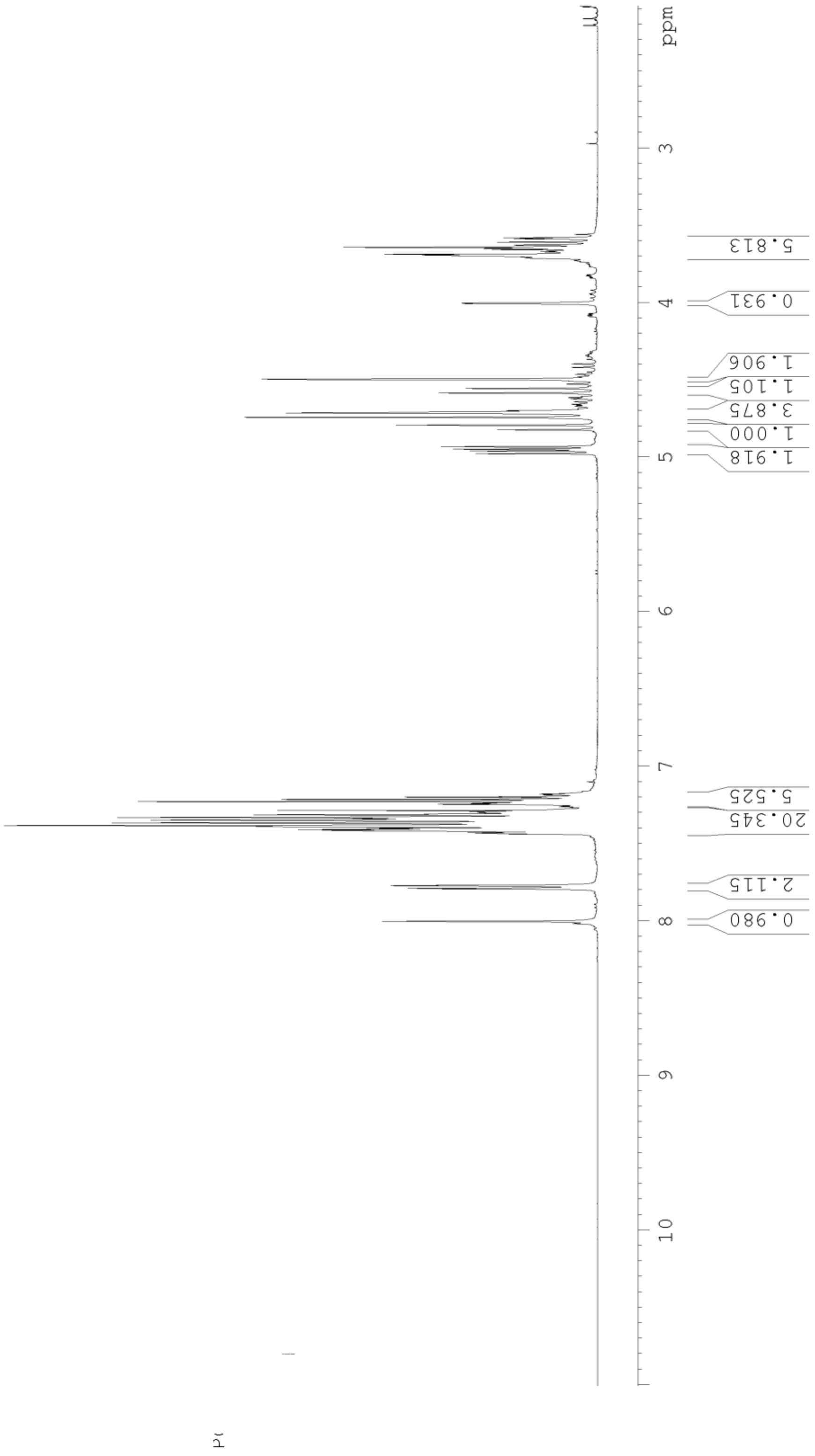

^{13}C:

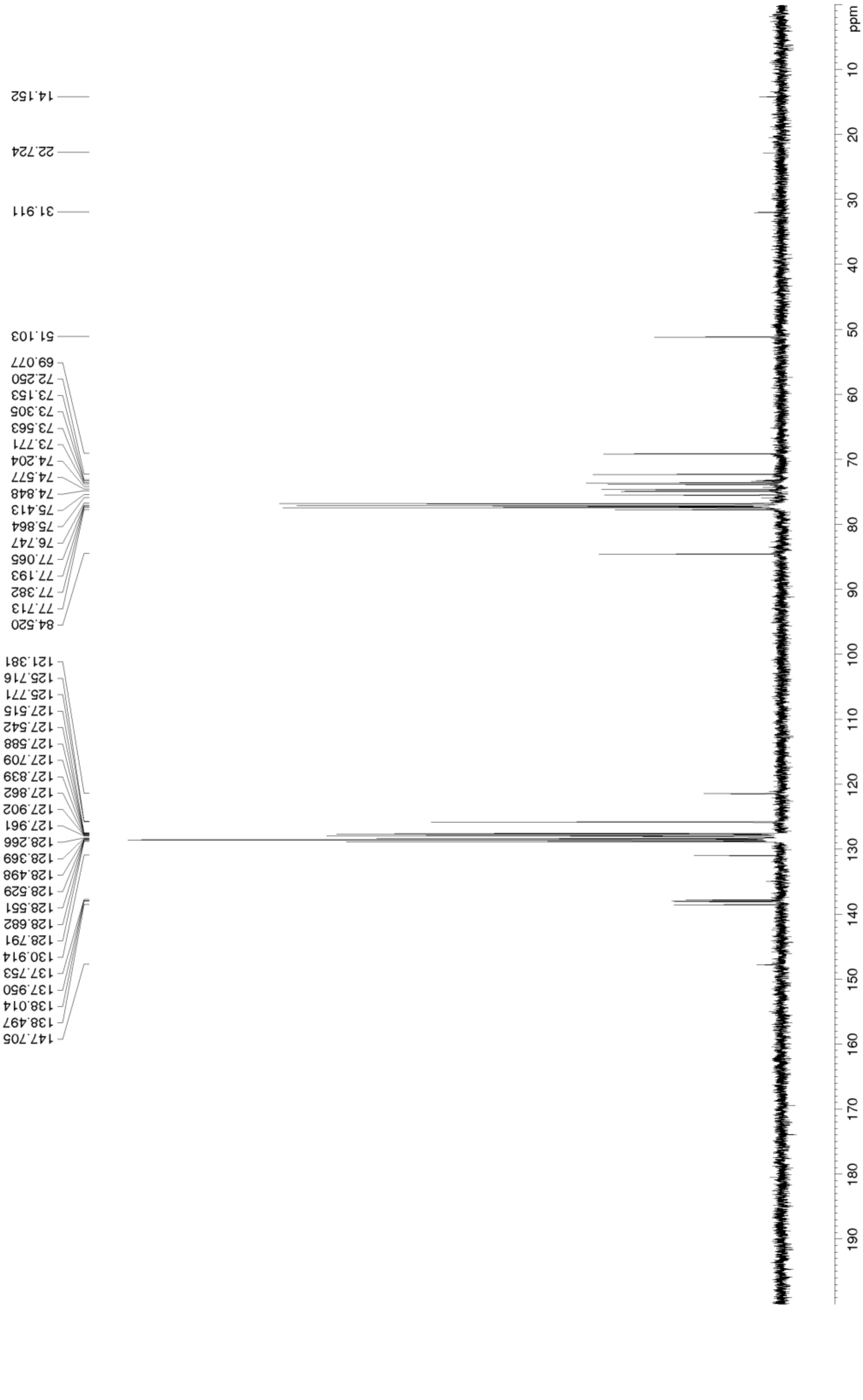
14.152
22.724
31.911
51.103
69.077
72.250
73.153
73.305
73.563
73.771
74.204
74.577
74.848
75.413
75.864
76.747
77.065
77.193
77.382
77.713
84.520
121.381
125.716
125.771
127.515
127.542
127.588
127.709
127.839
127.862
127.902
127.961
128.266
128.369
128.498
128.529
128.551
128.682
128.791
130.914
137.753
137.950
138.014
138.497
147.705
ppm
10
20
30
40
50
60
70
80
90
100
110
120
130
140
150
160
170
180
190

Compound **10b**:

^{1}H:

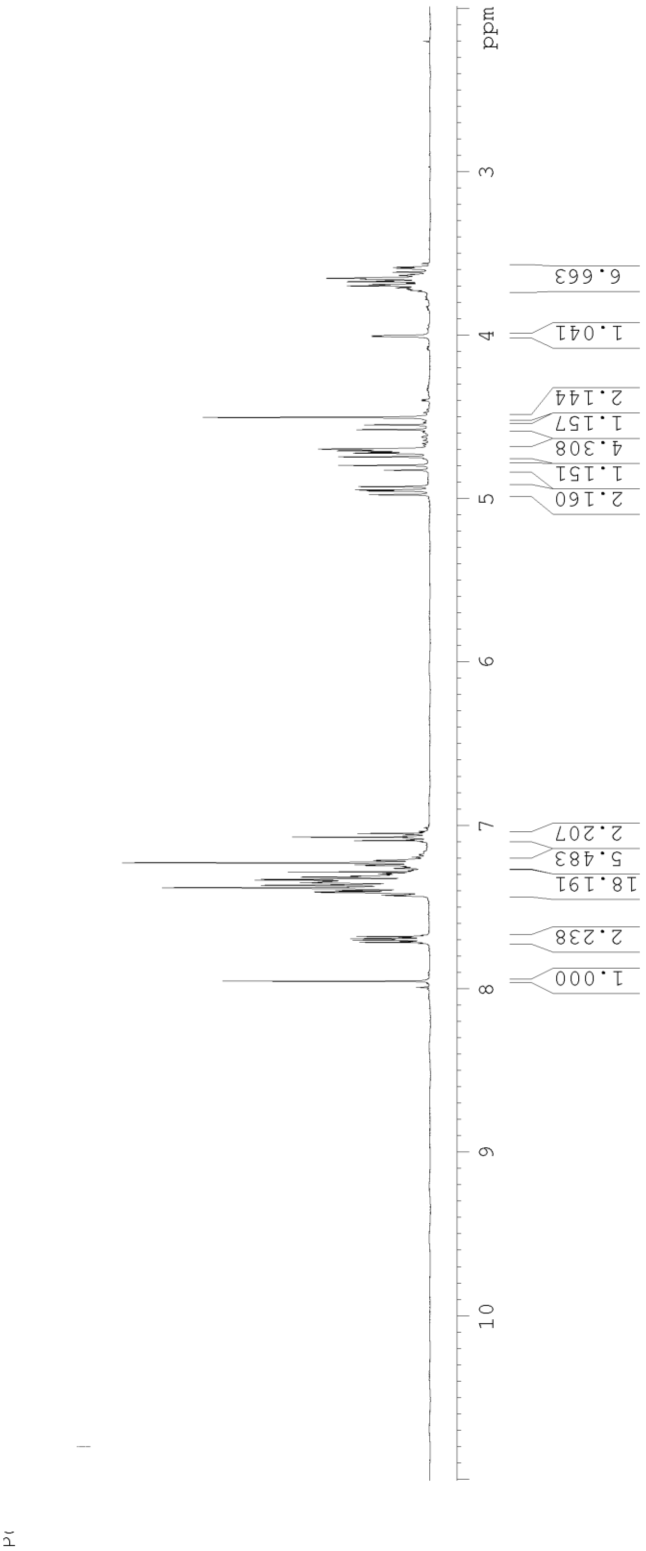

^{13}C:

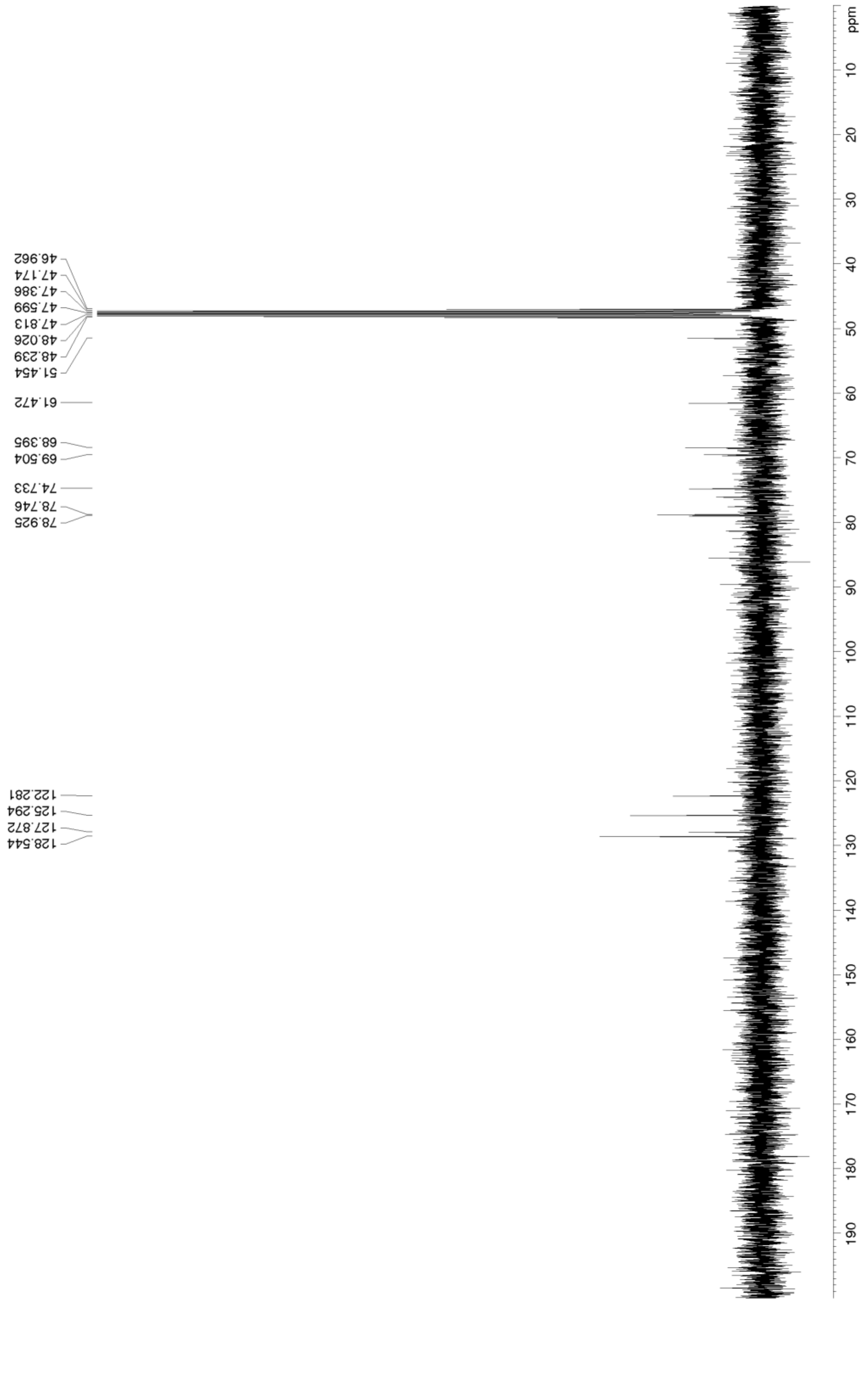

46.962
47.174
47.386
47.599
47.813
48.026
48.239
51.454
61.472
68.395
69.504
74.733
78.746
78.925
122.281
125.294
127.872
128.544
ppm

Compound **10c**:

^{1}H:

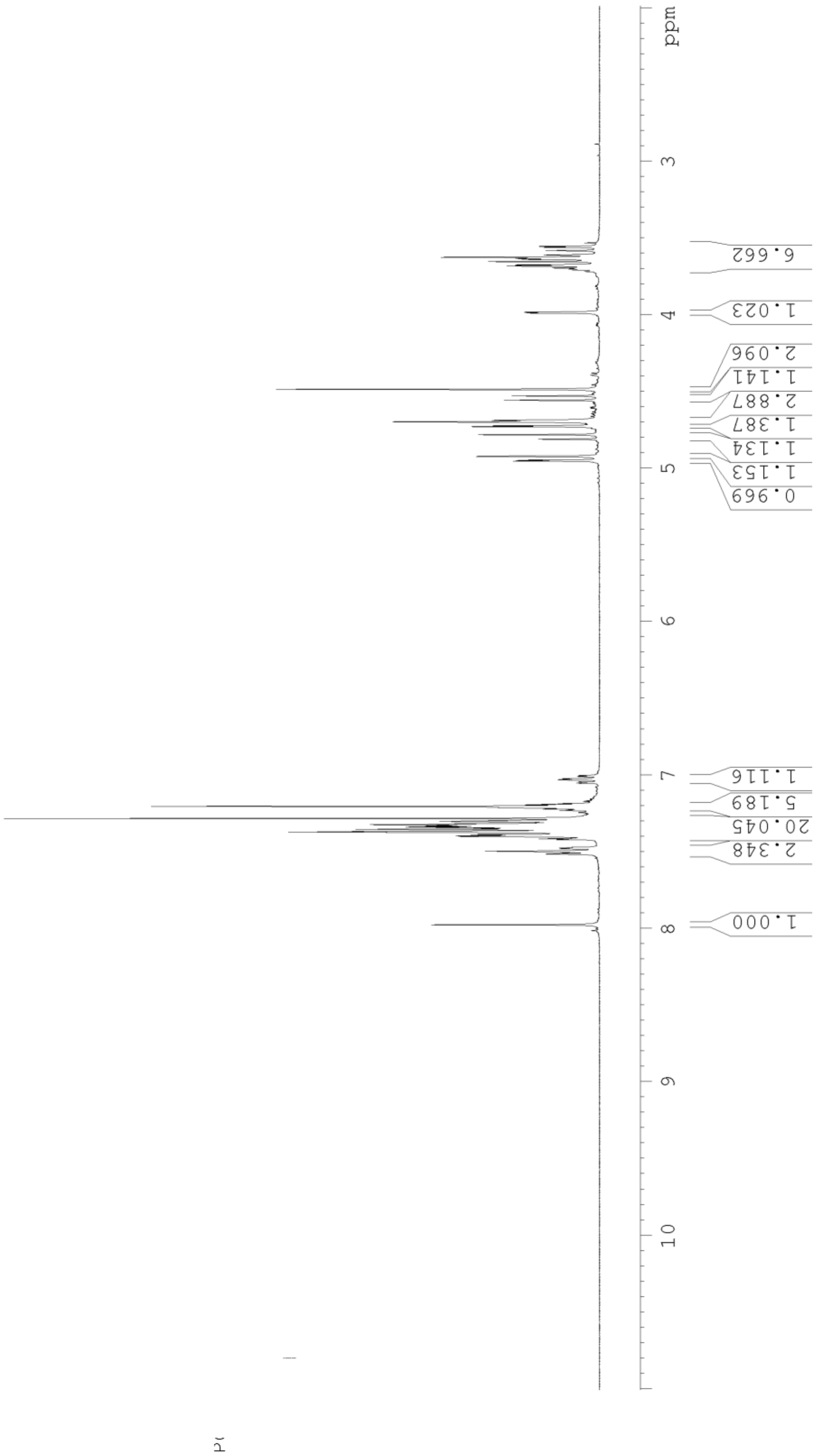

^{13}C:

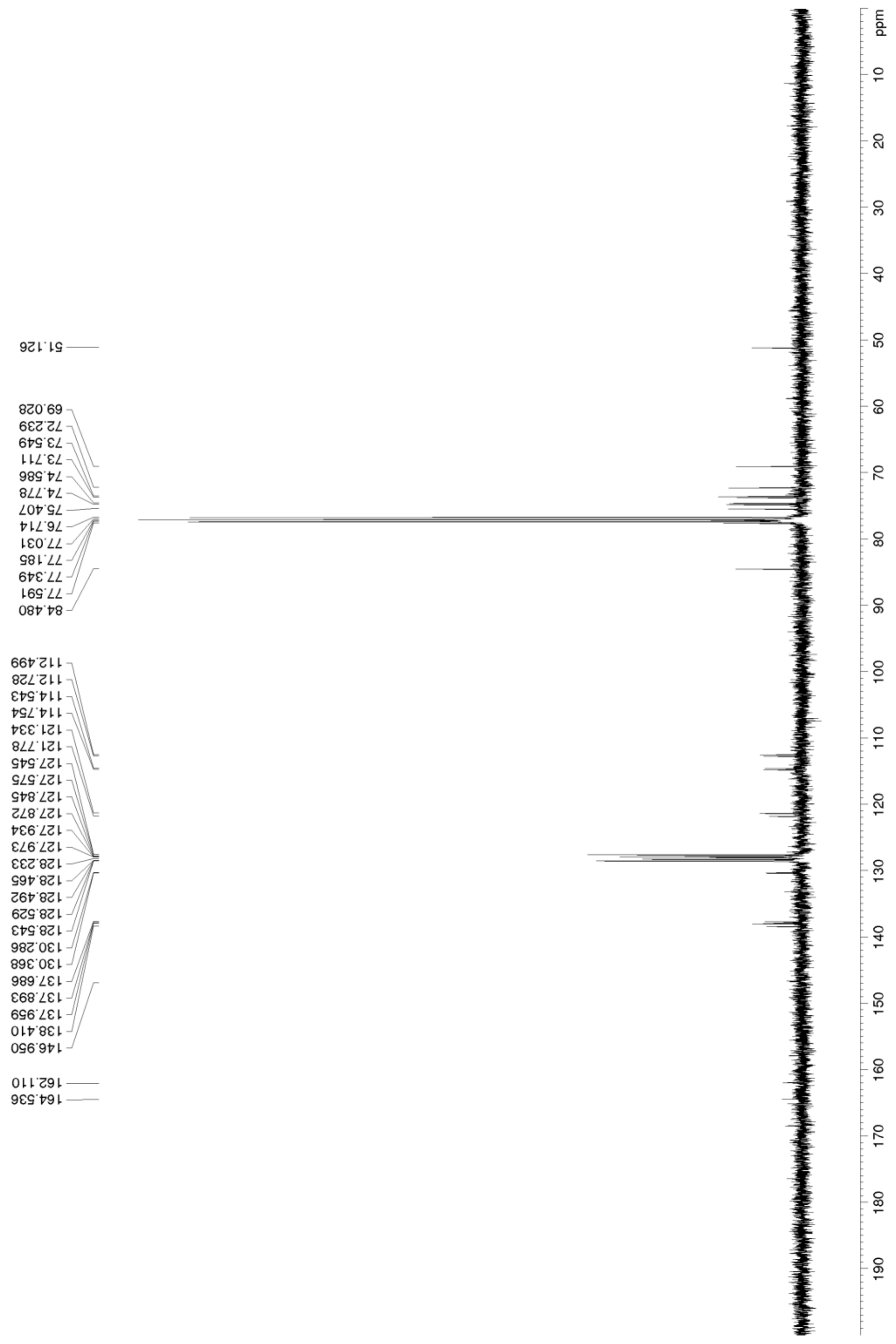

51.126
69.028
72.239
73.549
73.711
74.586
74.778
75.407
76.714
77.031
77.185
77.349
77.591
84.480
112.499
112.728
114.543
114.754
121.334
121.778
127.545
127.575
127.845
127.872
127.934
127.973
128.233
128.465
128.492
128.529
128.543
130.286
130.368
137.686
137.893
137.959
138.410
146.950
162.110
164.536
ppm
10
20
30
40
50
60
70
80
90
100
110
120
130
140
150
160
170
180
190

Compound **10d**:

^{1}H:

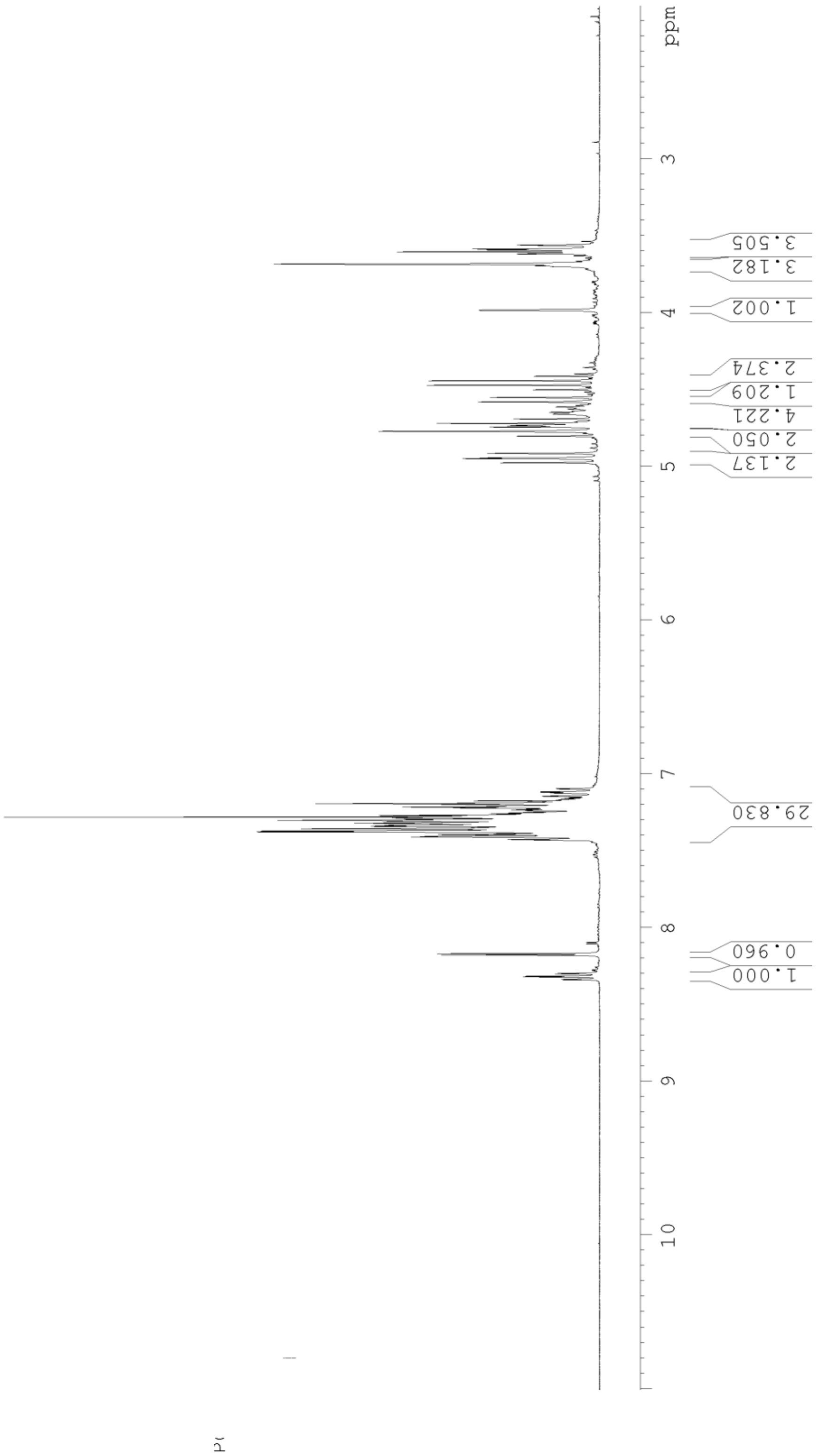

^{13}C:

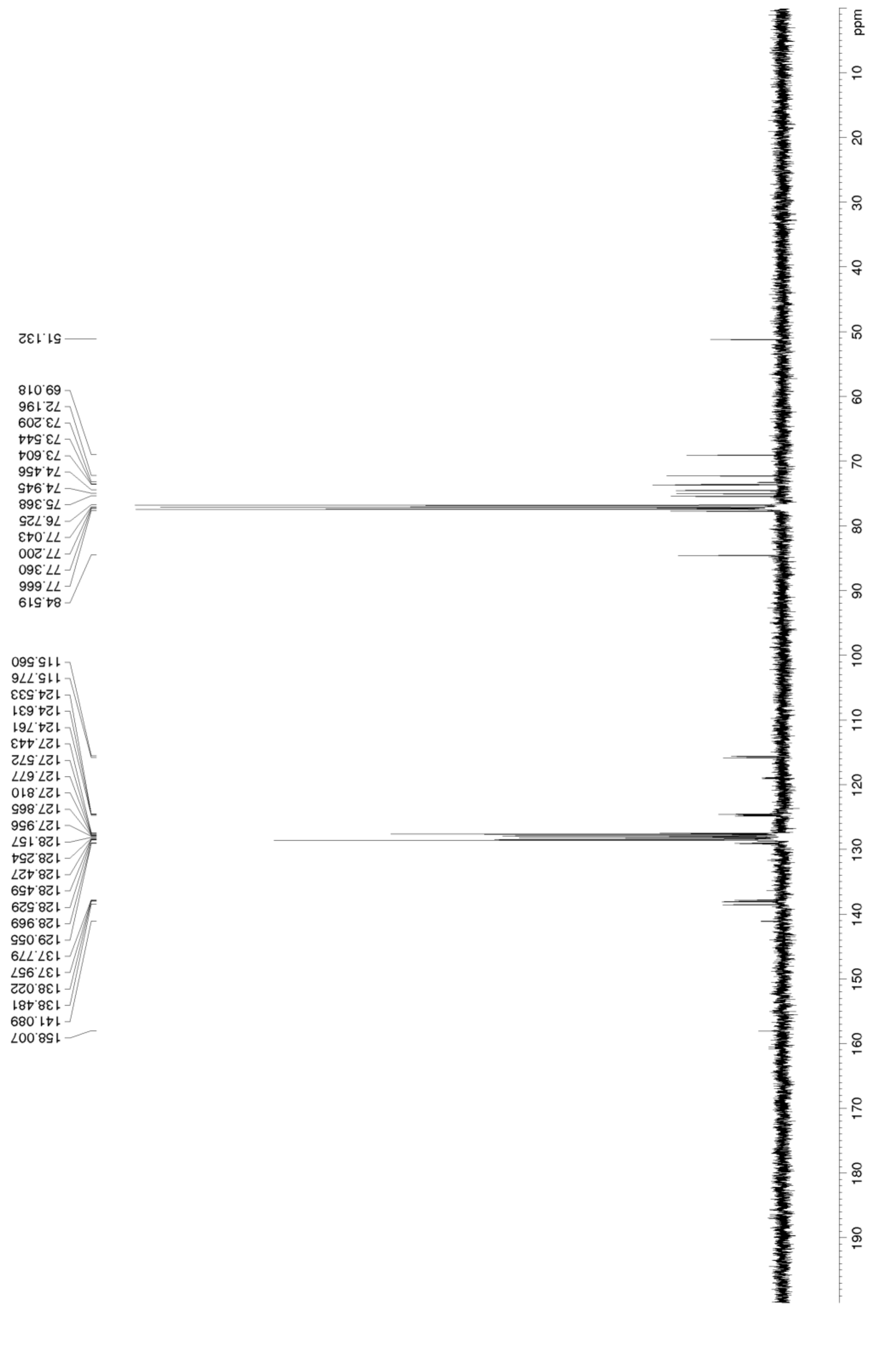
51.132
69.018
72.196
73.209
73.544
73.604
74.456
74.945
75.368
76.725
77.043
77.200
77.360
77.666
84.519
115.560
115.776
124.533
124.631
124.761
127.443
127.572
127.677
127.810
127.865
127.956
128.157
128.254
128.427
128.459
128.529
128.969
129.055
137.779
137.957
138.022
138.481
141.089
158.007
ppm
10
20
30
40
50
60
70
80
90
100
110
120
130
140
150
160
170
180
190

Compound **10e**:

^{1}H:

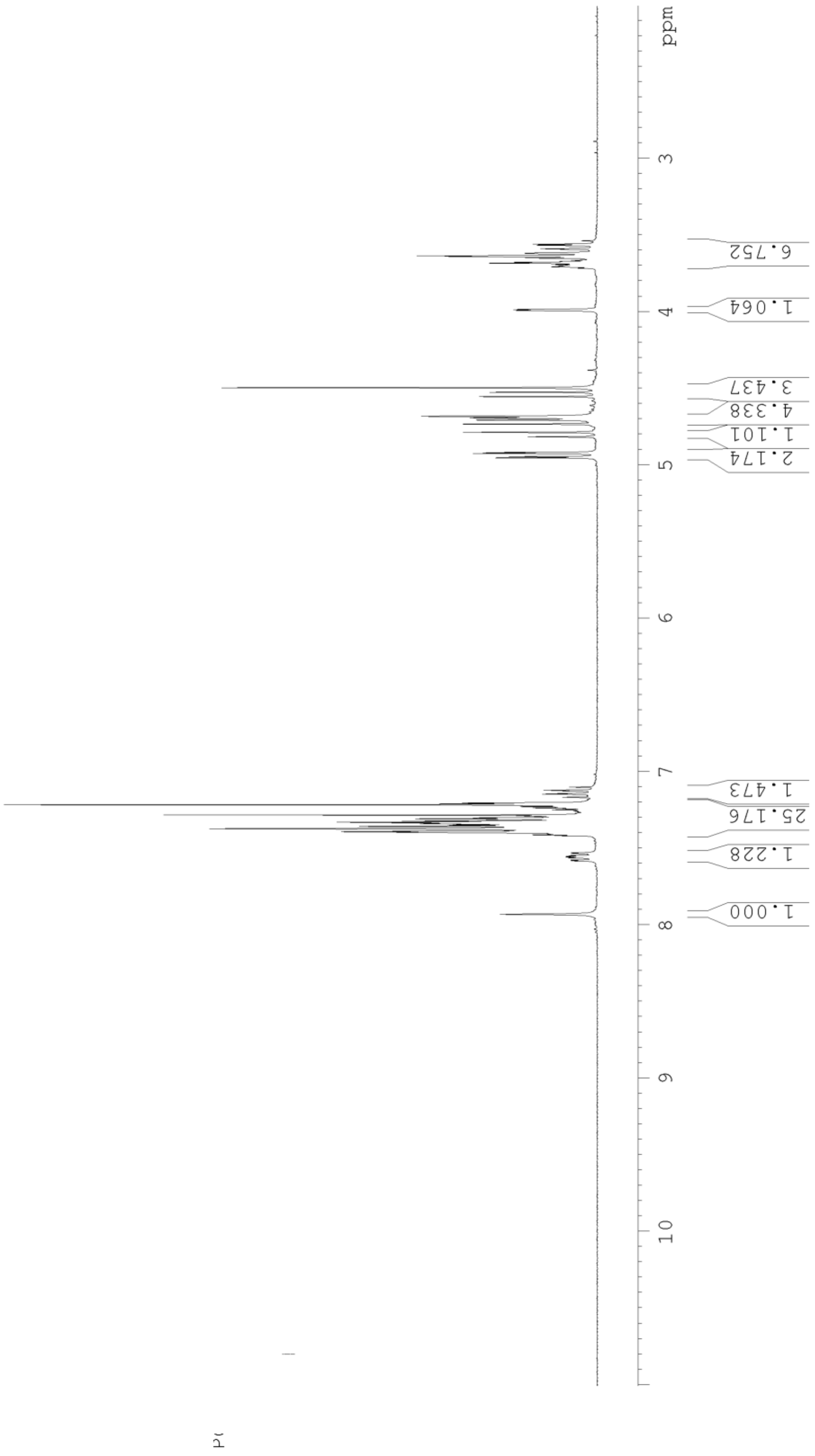

^{13}C:

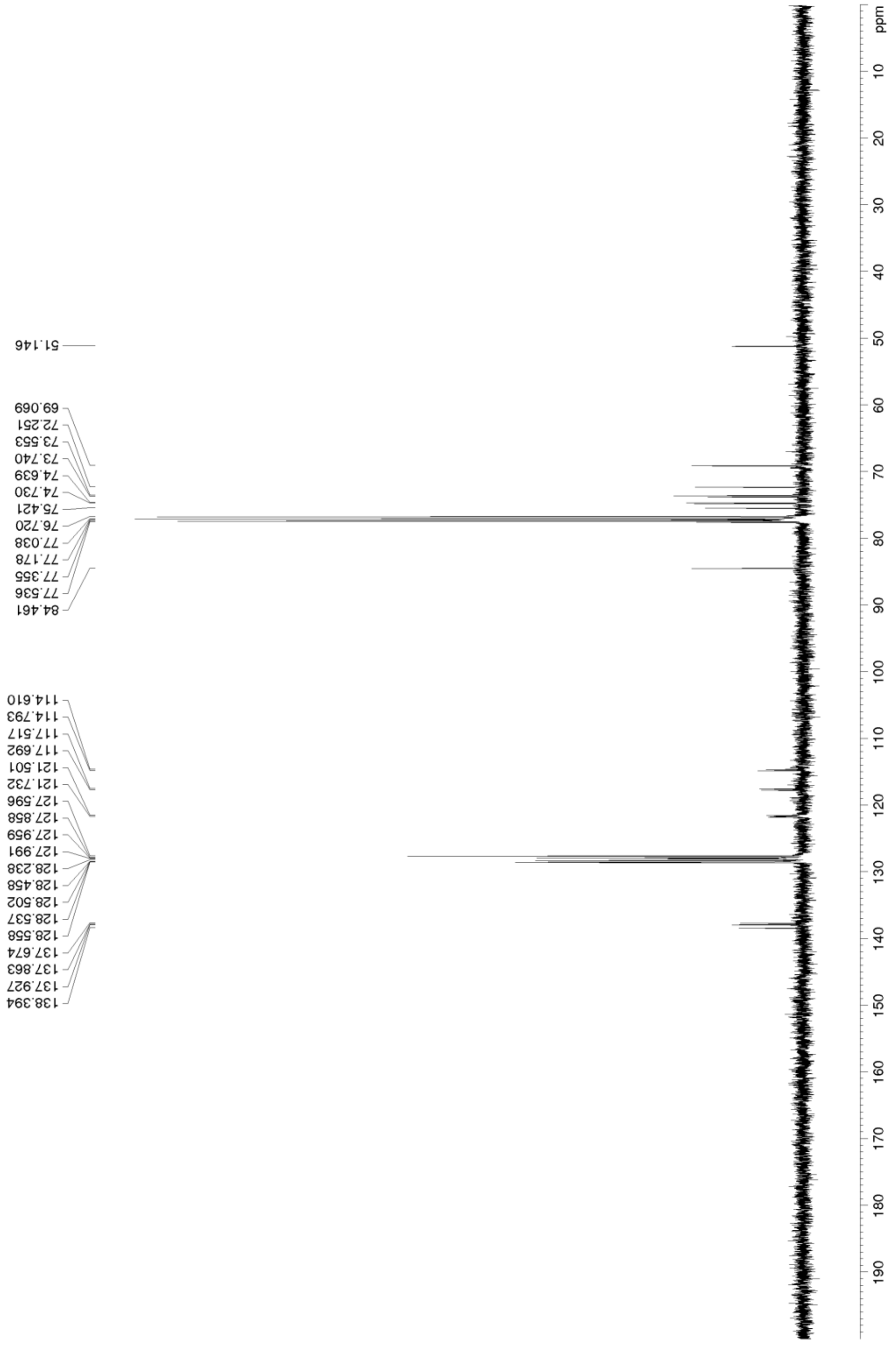
51.146
69.069
72.251
73.553
73.740
74.639
74.730
75.421
76.720
77.038
77.178
77.355
77.536
84.461
114.610
114.793
117.517
117.692
121.501
121.732
127.596
127.858
127.959
127.991
128.238
128.458
128.502
128.537
128.558
137.674
137.863
137.927
138.394
ppm
10
20
30
40
50
60
70
80
90
100
110
120
130
140
150
160
170
180
190

Compound **10f**:

^{1}H:

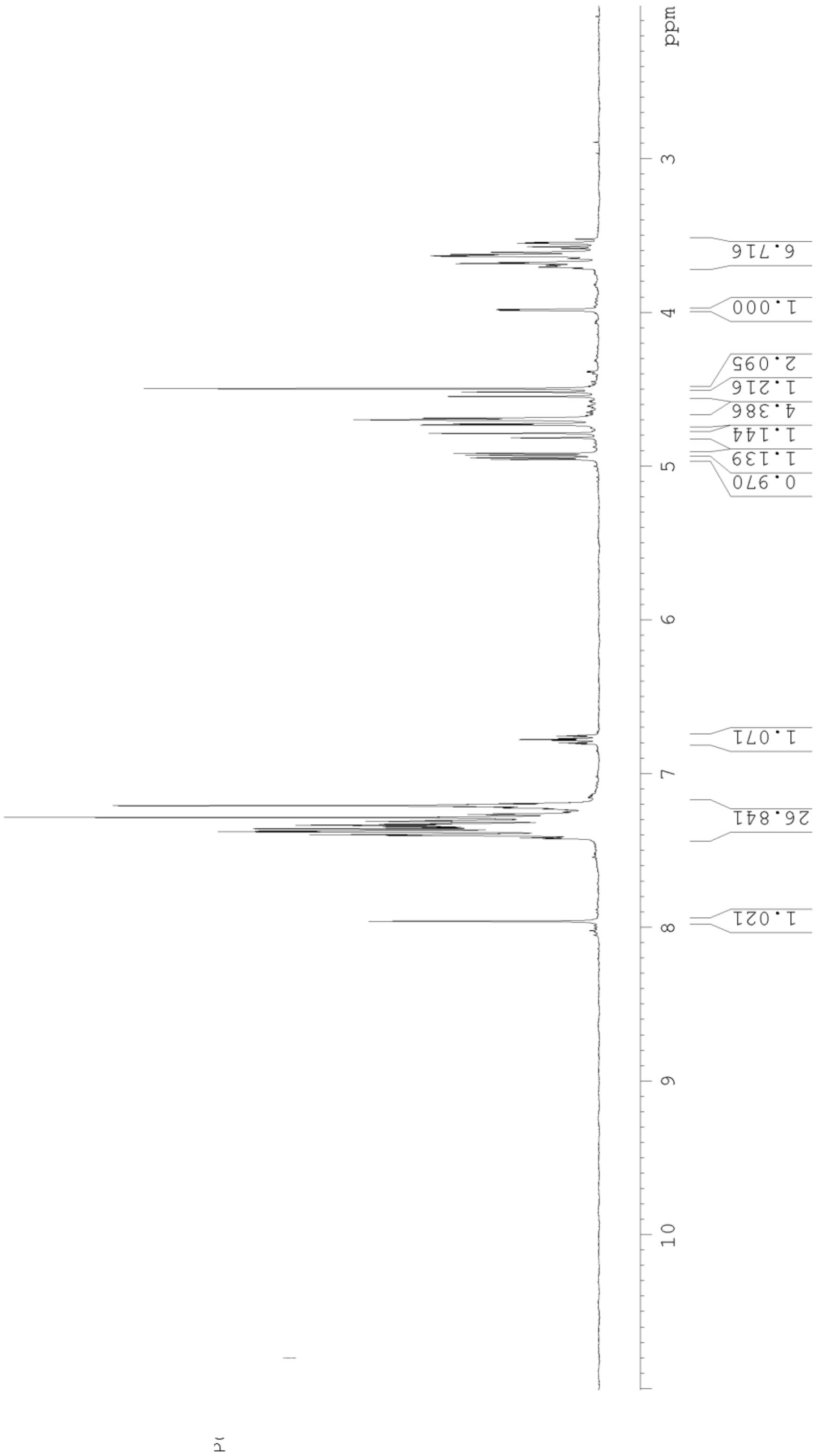

^{13}C:

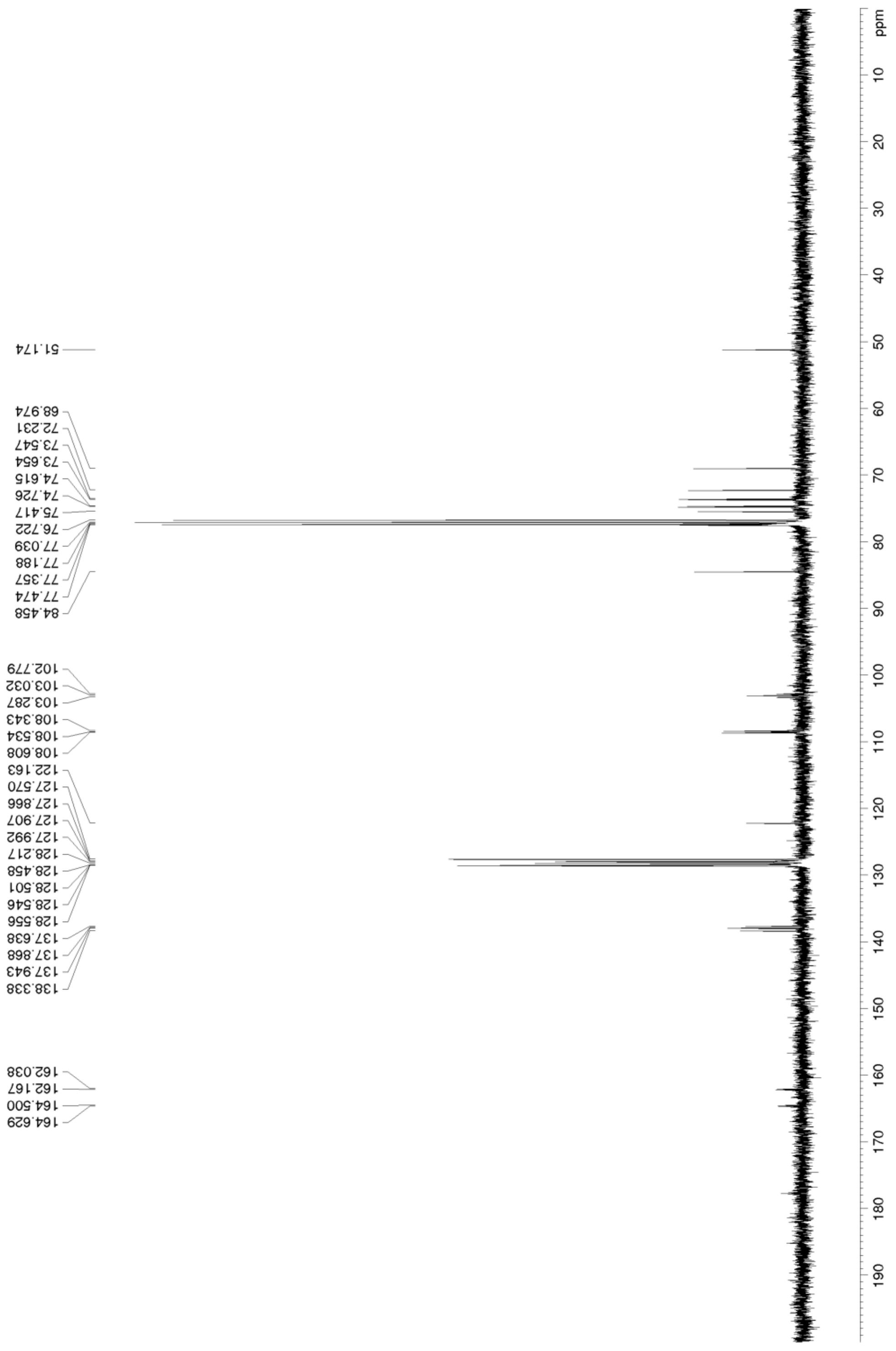
51.174
68.974
72.231
73.547
73.654
74.615
74.726
75.417
76.722
77.039
77.188
77.357
77.474
84.458
102.779
103.032
103.287
108.343
108.534
108.608
122.163
127.570
127.866
127.907
127.992
128.217
128.458
128.501
128.546
128.556
137.638
137.868
137.943
138.338
162.038
162.167
164.500
164.629
ppm
10
20
30
40
50
60
70
80
90
100
110
120
130
140
150
160
170
180
190

Compound **10g**:

^{1}H:

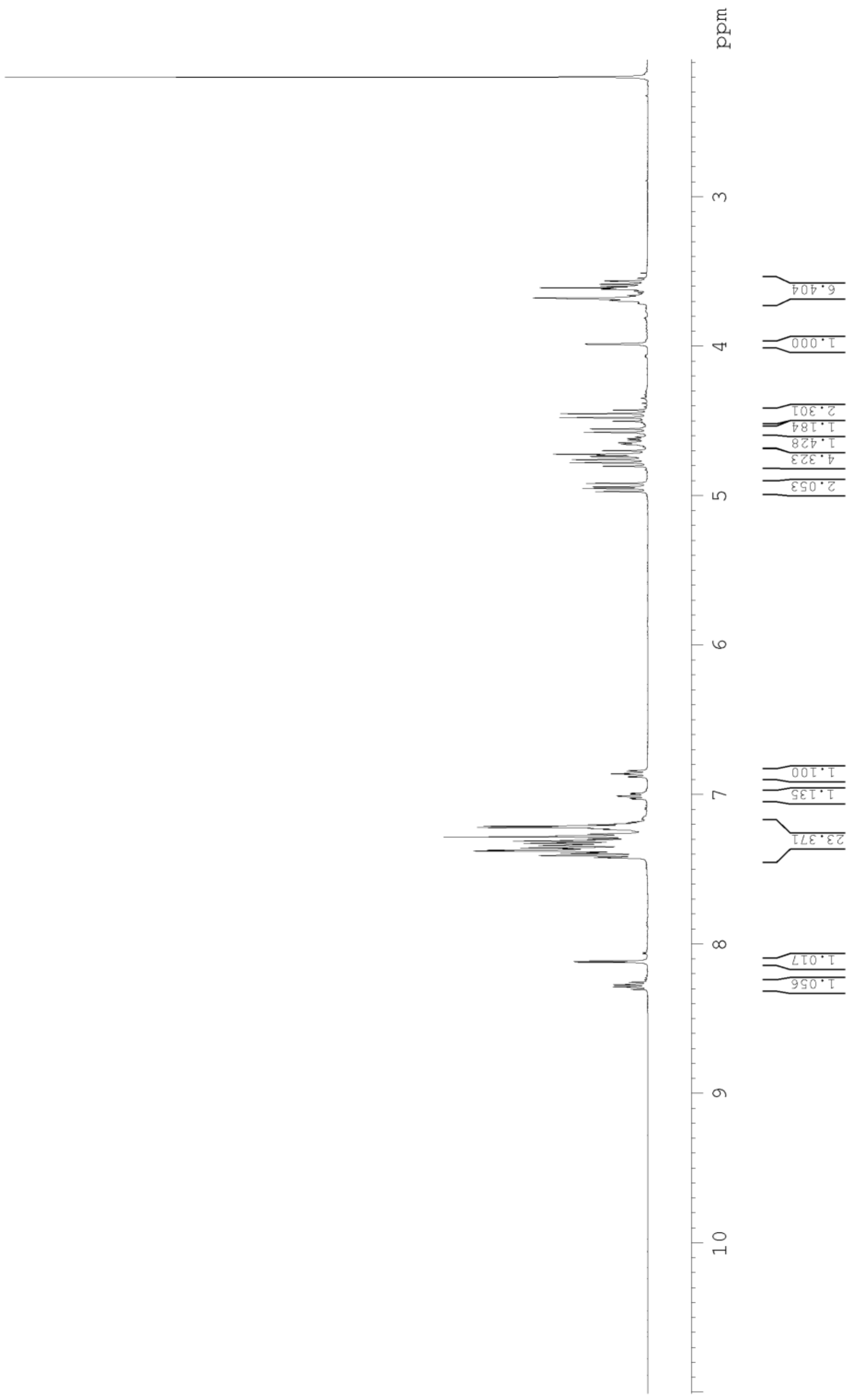

^{13}C:

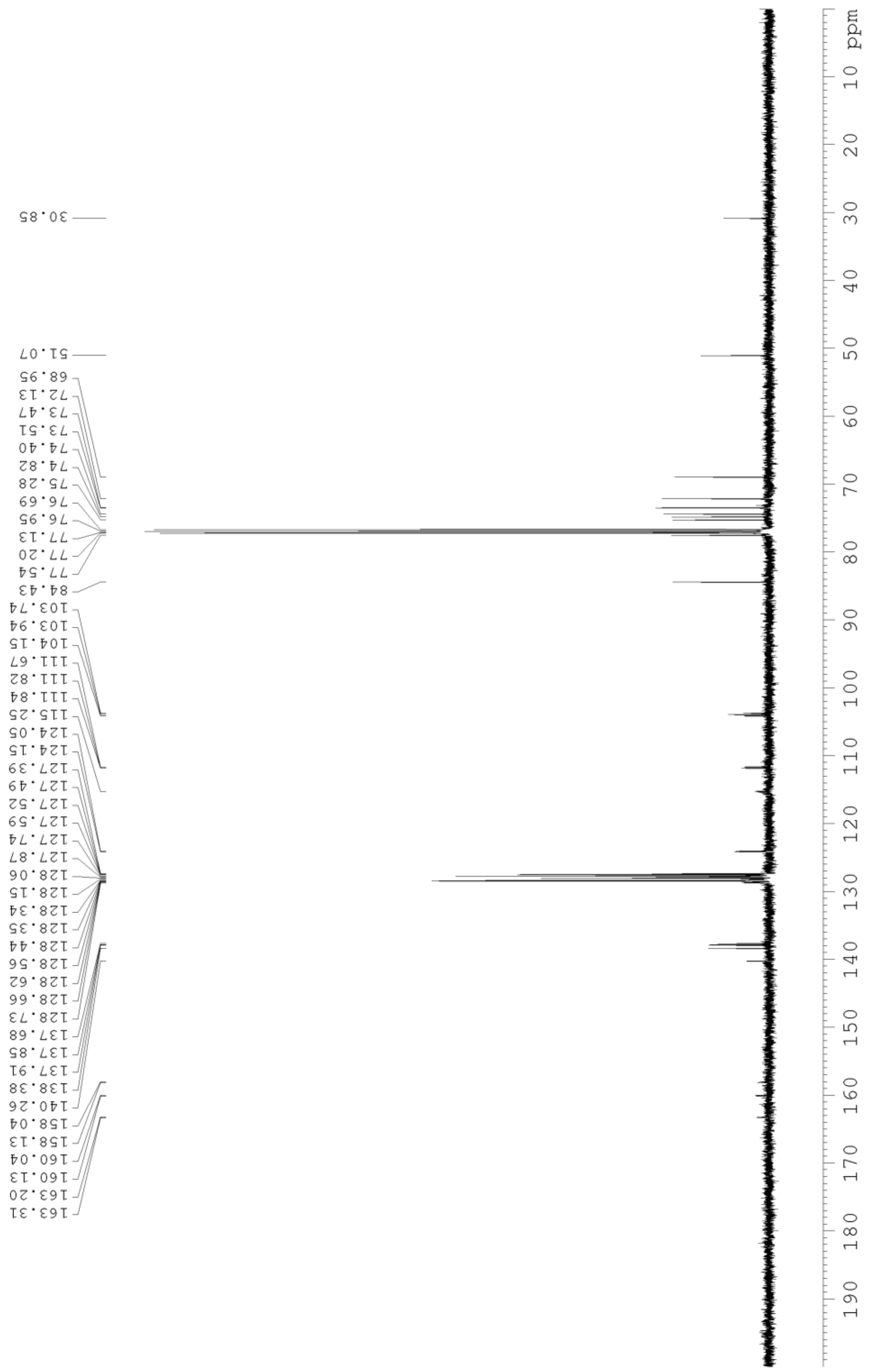
30.85
51.07
68.95
72.13
73.47
73.51
74.40
74.82
75.28
76.69
76.95
77.13
77.20
77.54
84.43
103.74
103.94
104.15
111.67
111.82
111.84
115.25
124.05
124.15
127.39
127.49
127.52
127.59
127.74
127.87
128.06
128.15
128.34
128.35
128.44
128.56
128.62
128.66
128.73
137.68
137.85
137.91
138.38
140.26
158.04
158.13
160.04
160.13
163.20
163.31
ppm
10
20
30
40
50
60
70
80
90
100
110
120
130
140
150
160
170
180
190

Compound **10h**:

^{1}H:

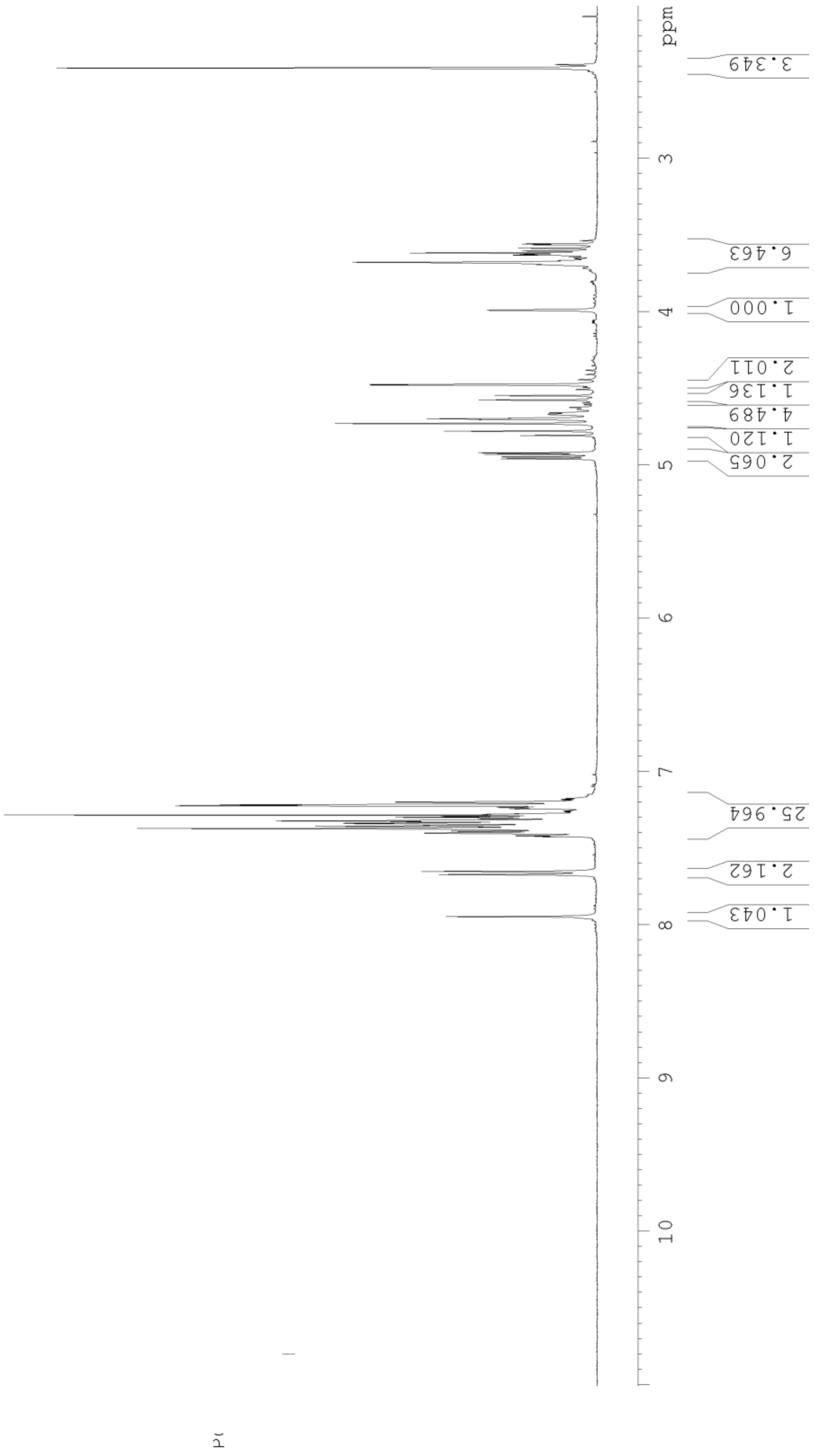

^{13}C:

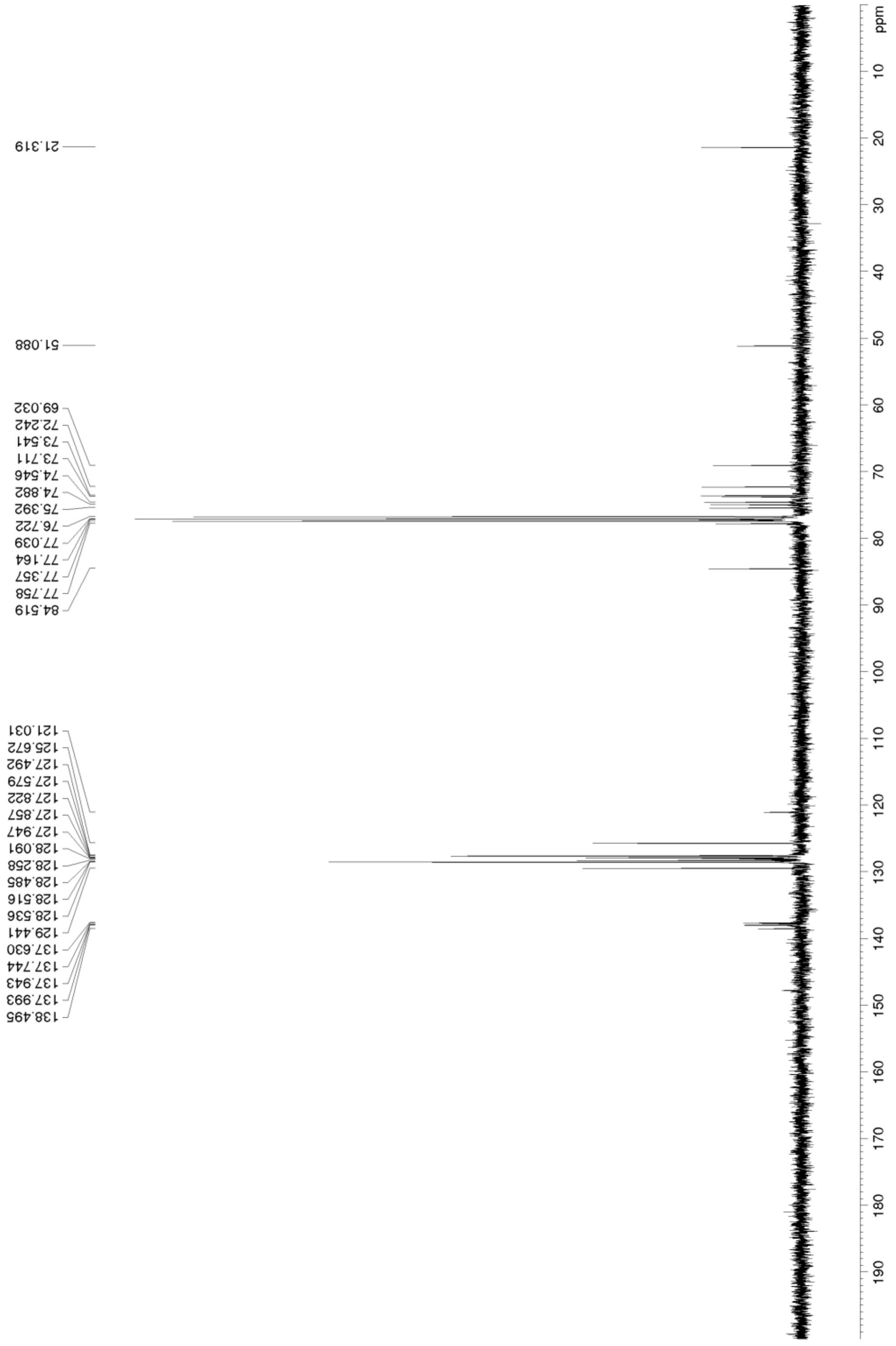
21.319
51.088
69.032
72.242
73.541
73.711
74.546
74.882
75.392
76.722
77.039
77.164
77.357
77.758
84.519
121.031
125.672
127.492
127.579
127.822
127.857
127.947
128.091
128.258
128.485
128.516
128.536
129.441
137.630
137.744
137.943
137.993
138.495
ppm

Compound **10i**:

^1H:

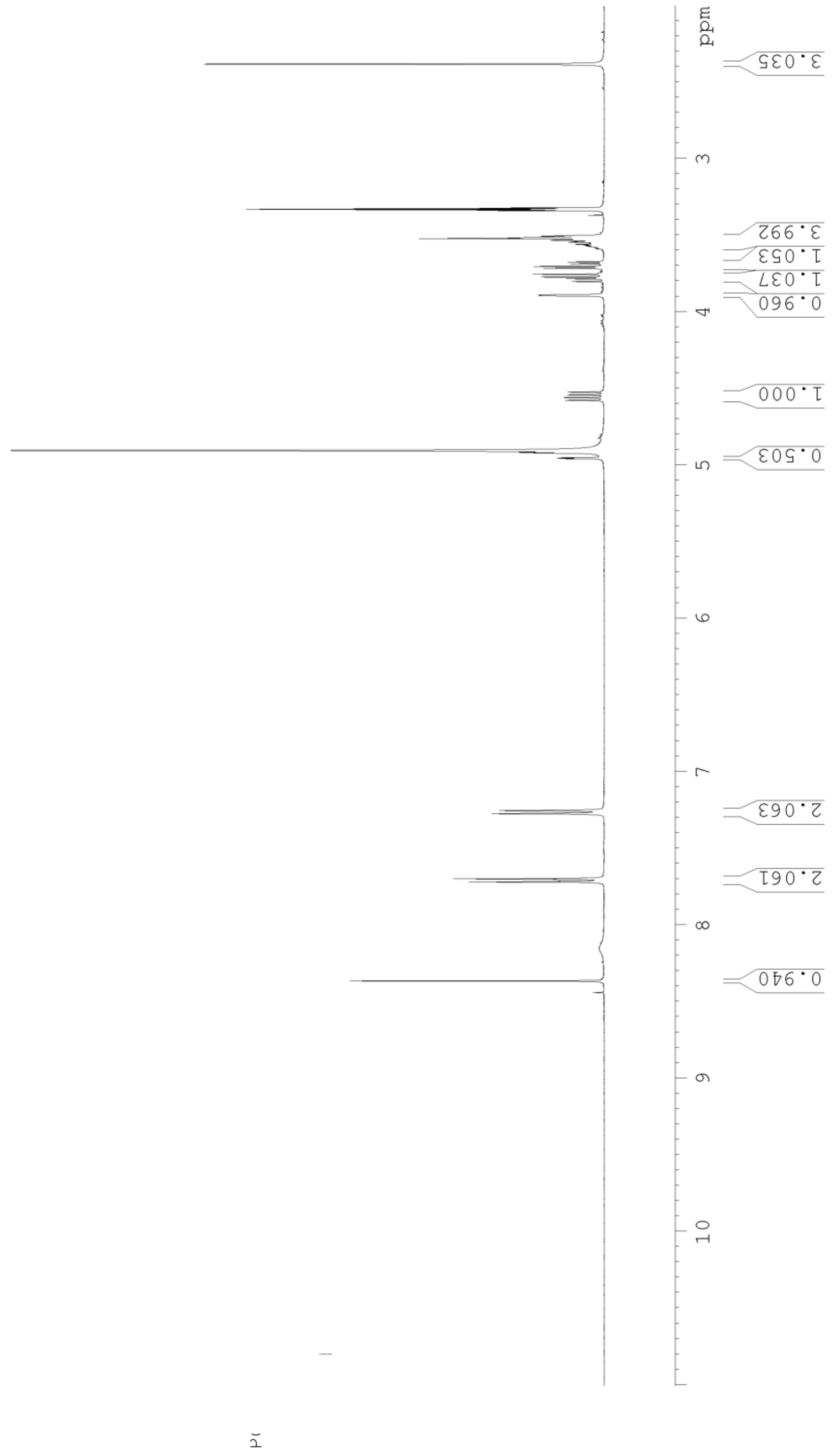

^{13}C:

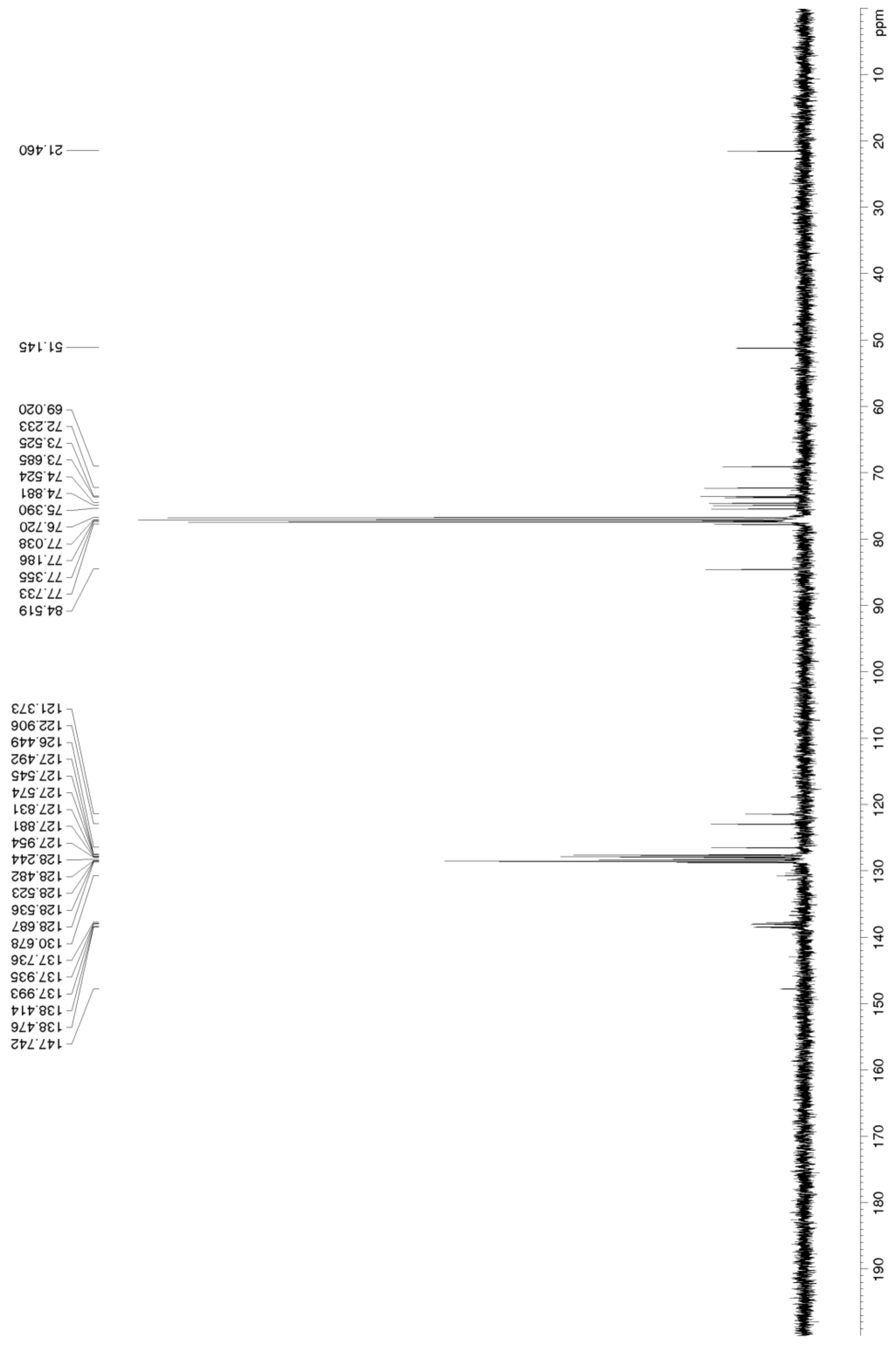
21.460
51.145
69.020
72.233
73.525
73.685
74.524
74.881
75.390
76.720
77.038
77.186
77.355
77.733
84.519
121.373
122.906
126.449
127.492
127.545
127.574
127.831
127.881
127.954
128.244
128.482
128.523
128.536
128.687
130.678
137.736
137.935
137.993
138.414
138.476
147.742
ppm
10
20
30
40
50
60
70
80
90
100
110
120
130
140
150
160
170
180
190

Compound **10j**:

^{1}H:

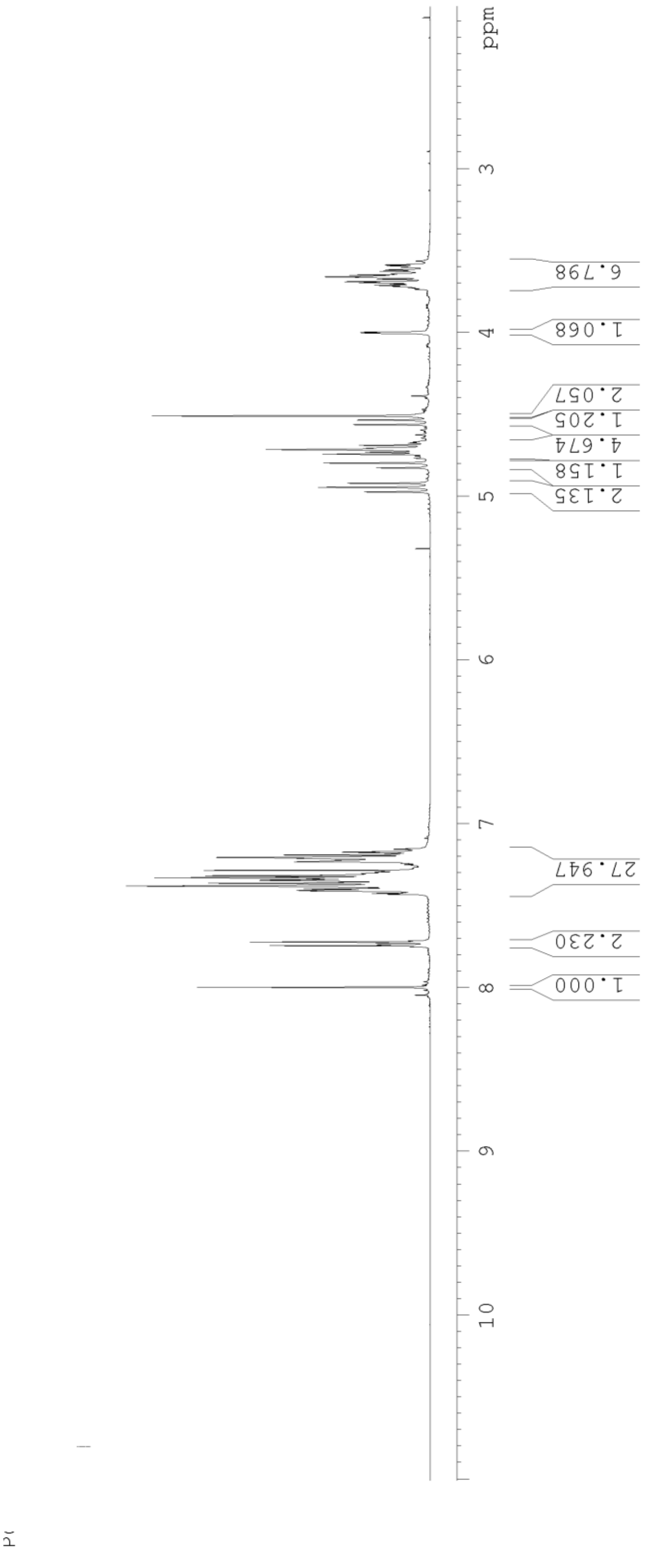

^{13}C:

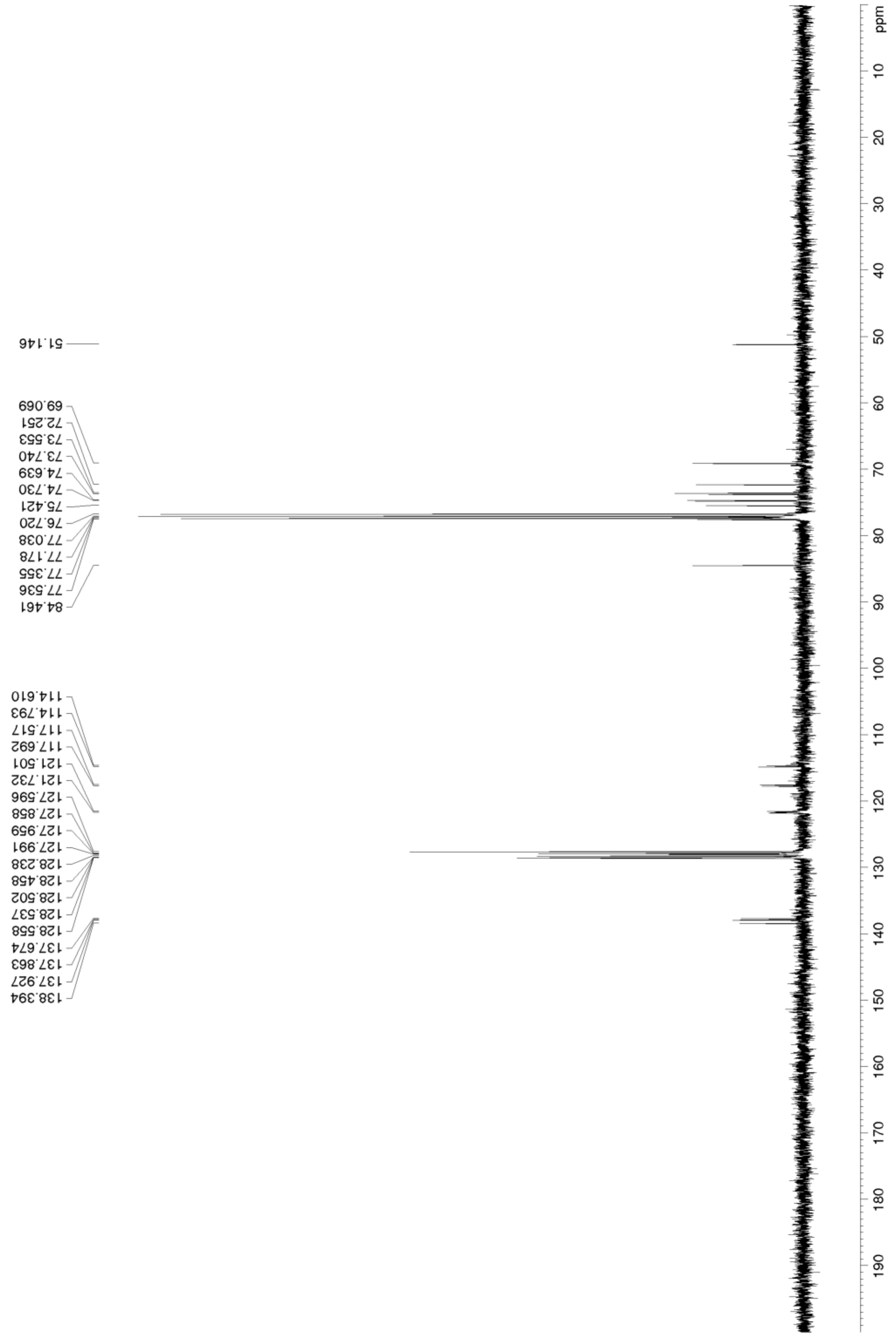
51.146
69.069
72.251
73.553
73.740
74.639
74.730
75.421
76.720
77.038
77.178
77.355
77.536
84.461
114.610
114.793
117.517
117.692
121.501
121.732
127.596
127.858
127.959
127.991
128.238
128.458
128.502
128.537
128.558
137.674
137.863
137.927
138.394
ppm
10
20
30
40
50
60
70
80
90
100
110
120
130
140
150
160
170
180
190

Compound **10k**:

^{1}H:

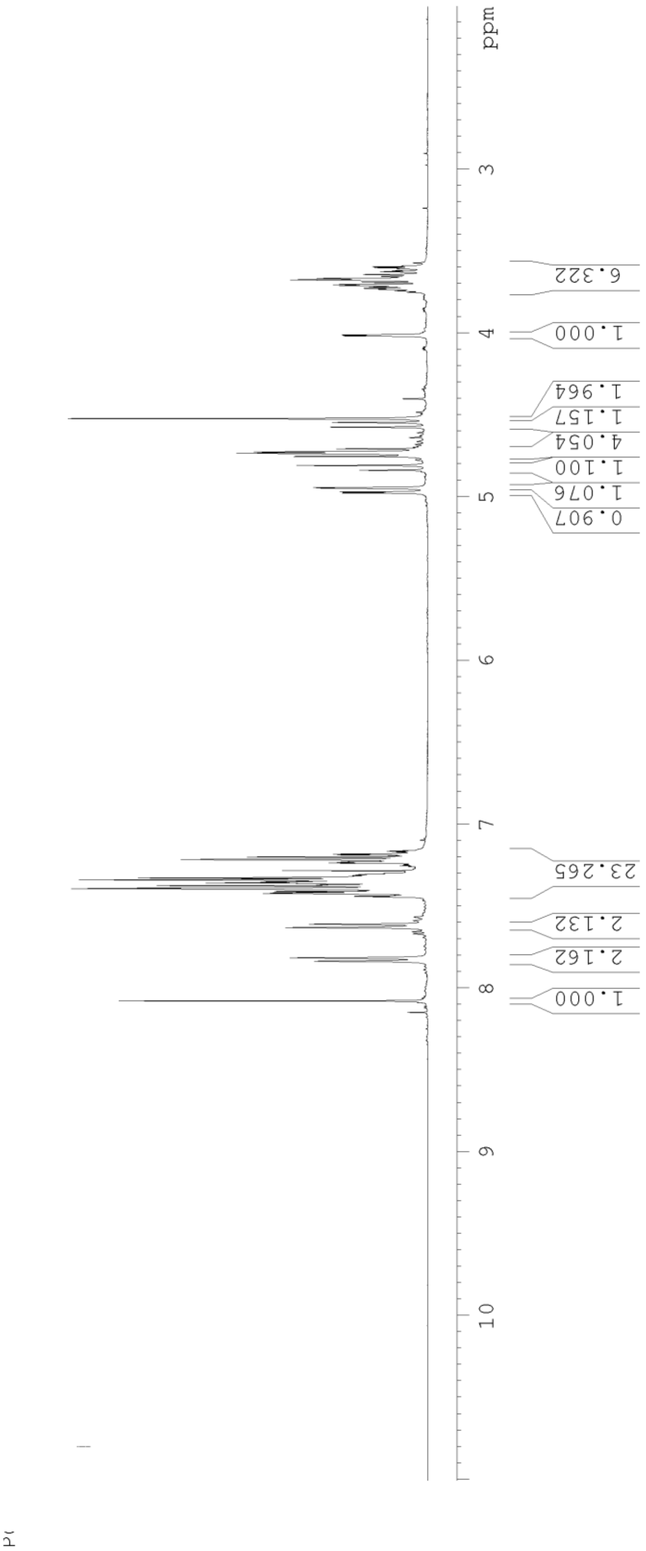

^{13}C:

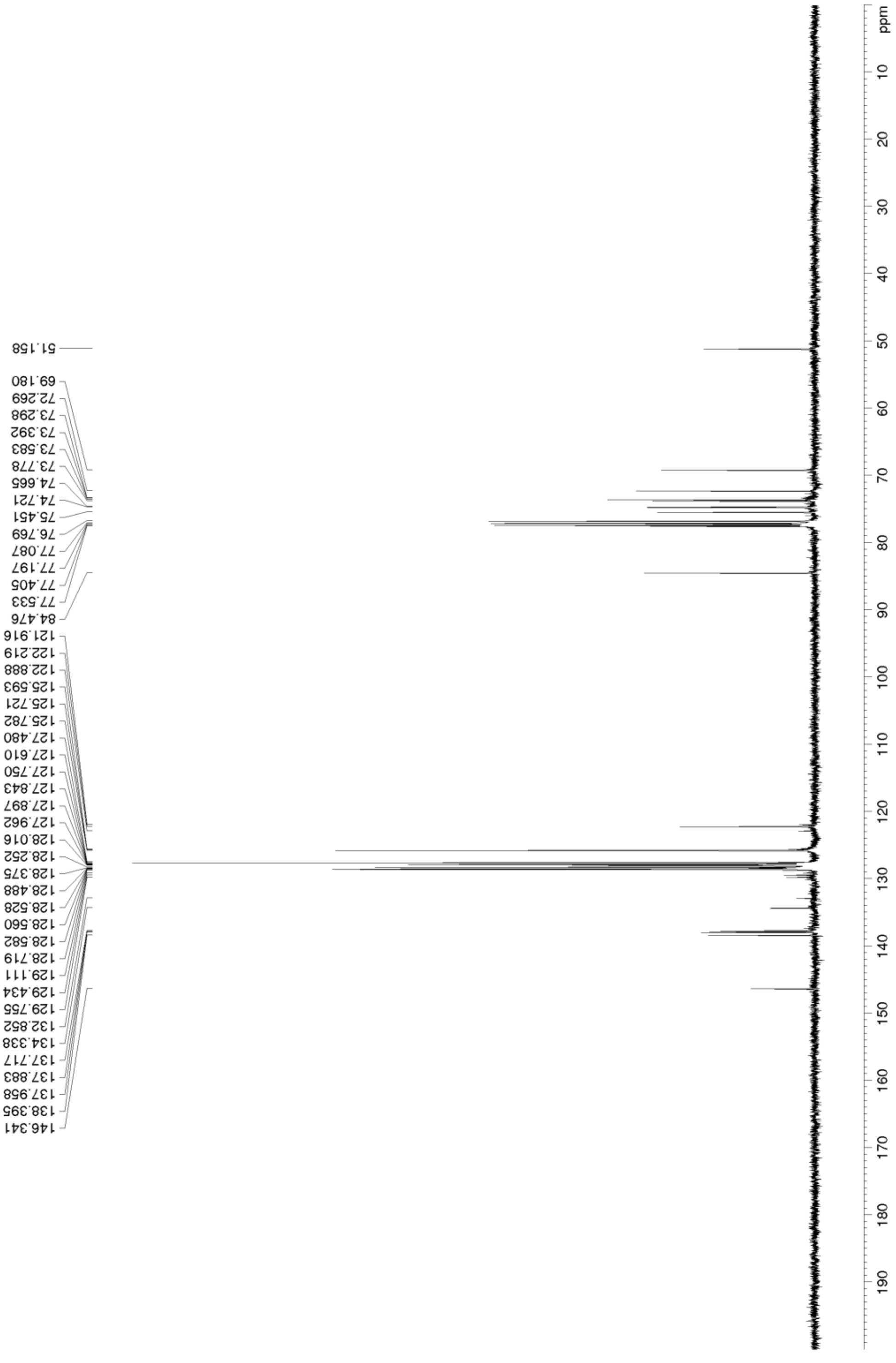
51.158
69.180
72.269
73.298
73.392
73.583
73.778
74.665
74.721
75.451
76.769
77.087
77.197
77.405
77.533
84.476
121.916
122.219
122.888
125.593
125.721
125.782
127.480
127.610
127.750
127.843
127.897
127.962
128.016
128.252
128.375
128.488
128.528
128.560
128.582
128.719
129.111
129.434
129.755
132.852
134.338
137.717
137.883
137.958
138.395
146.341
ppm
10
20
30
40
50
60
70
80
90
100
110
120
130
140
150
160
170
180
190

Compound **10l**:

^{1}H:

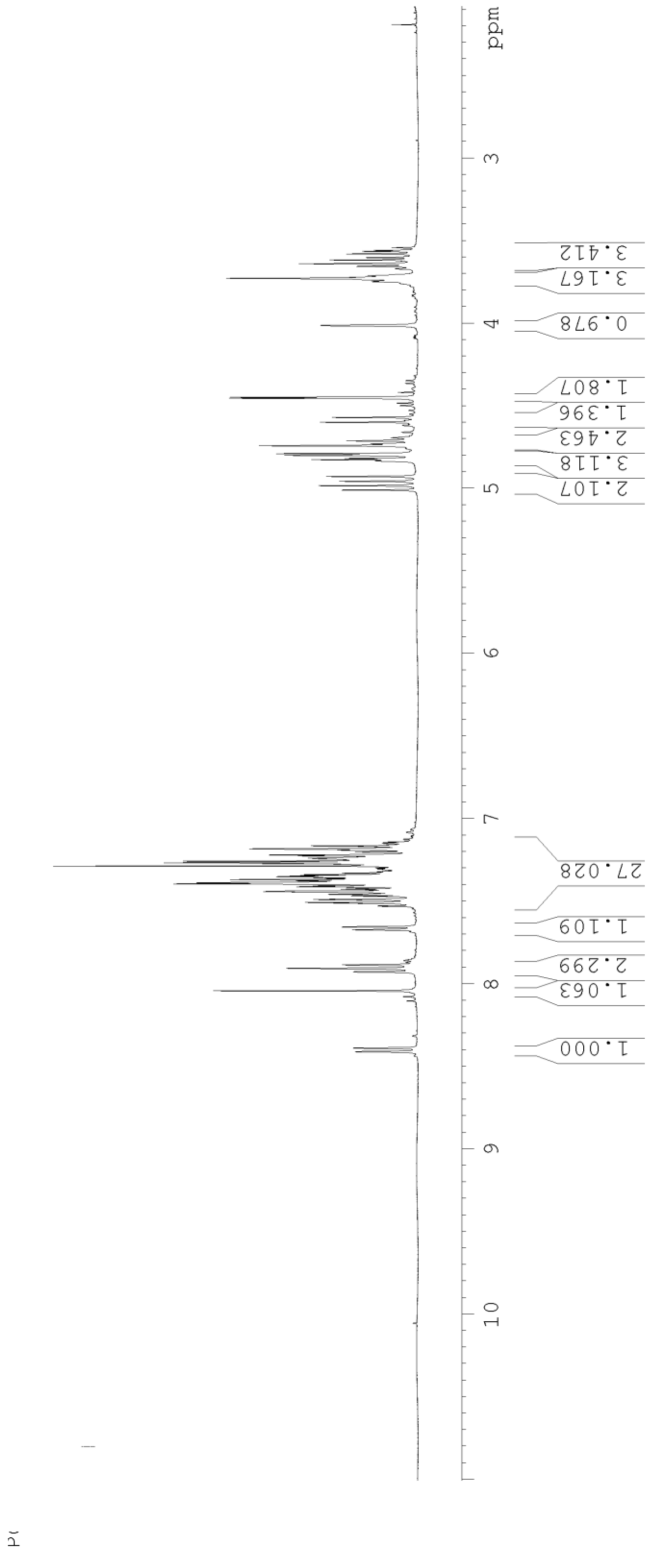

^{13}C:

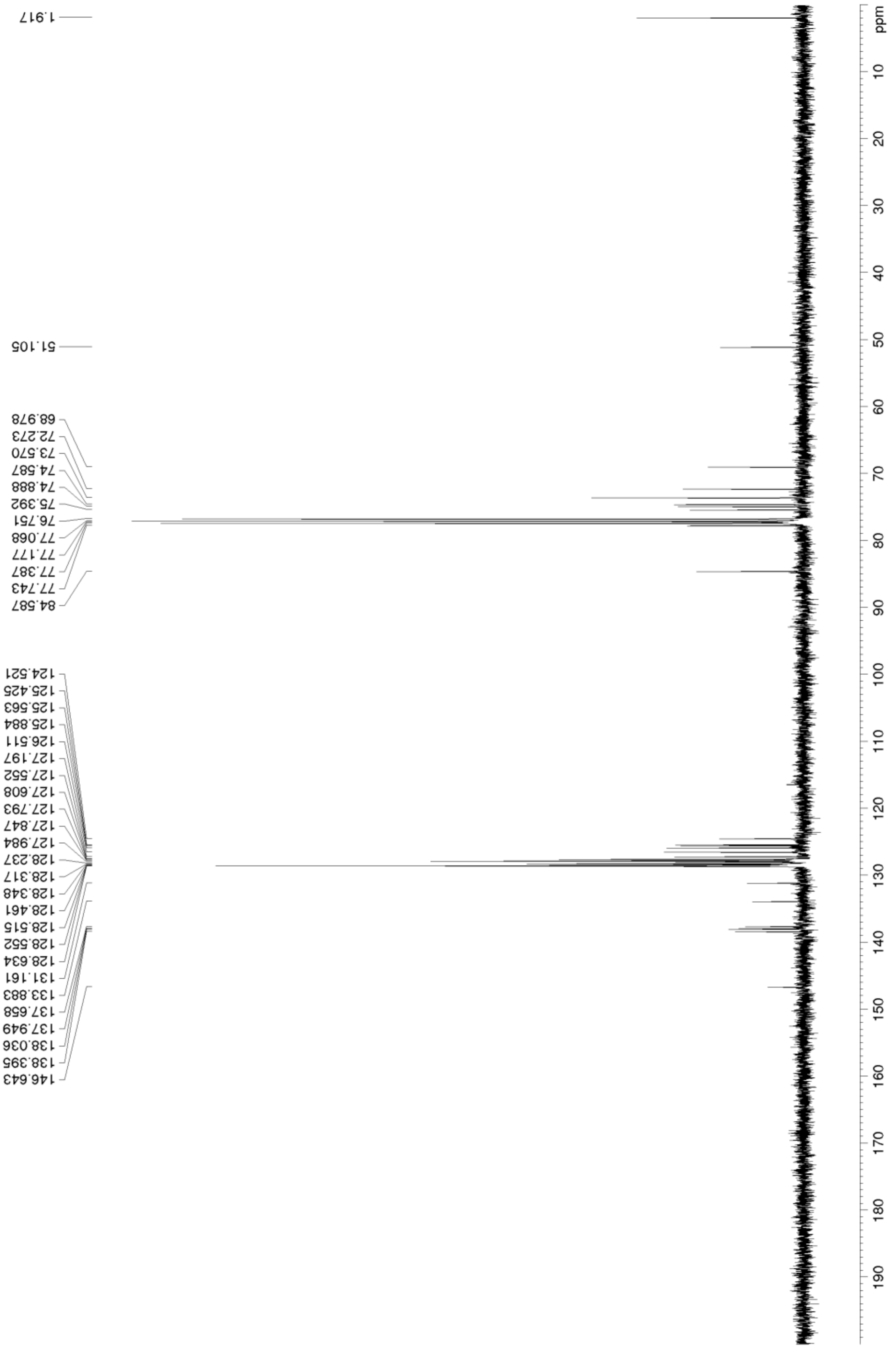
1.917
51.105
68.978
72.273
73.570
74.587
74.888
75.392
76.751
77.068
77.177
77.387
77.743
84.587
124.521
125.425
125.563
125.884
126.511
127.197
127.552
127.608
127.793
127.847
127.984
128.237
128.317
128.348
128.461
128.515
128.552
128.634
131.161
133.883
137.658
137.949
138.036
138.395
146.643
ppm
10
20
30
40
50
60
70
80
90
100
110
120
130
140
150
160
170
180
190

Compound **10m**:

^{1}H:

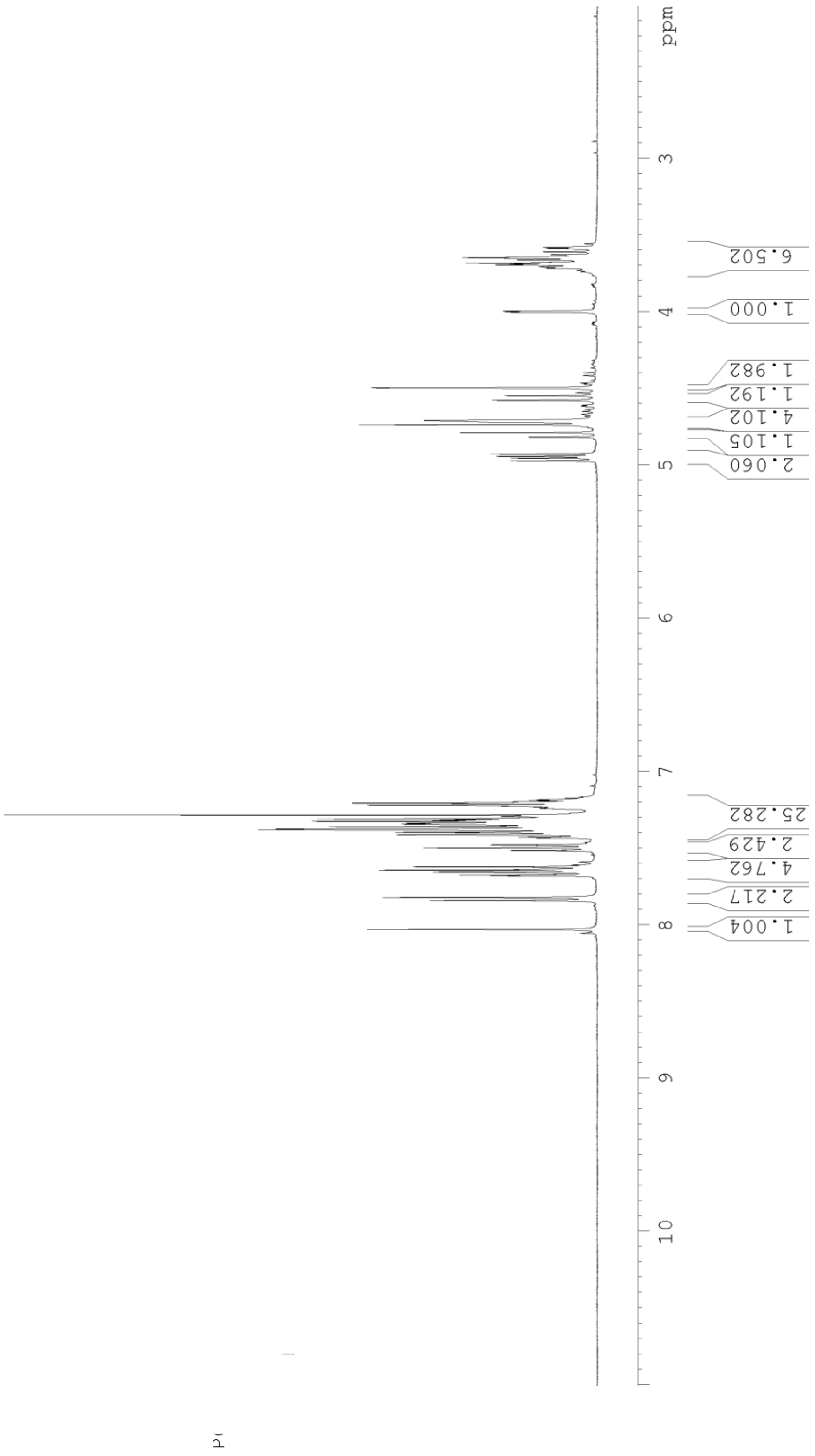

^{13}C:

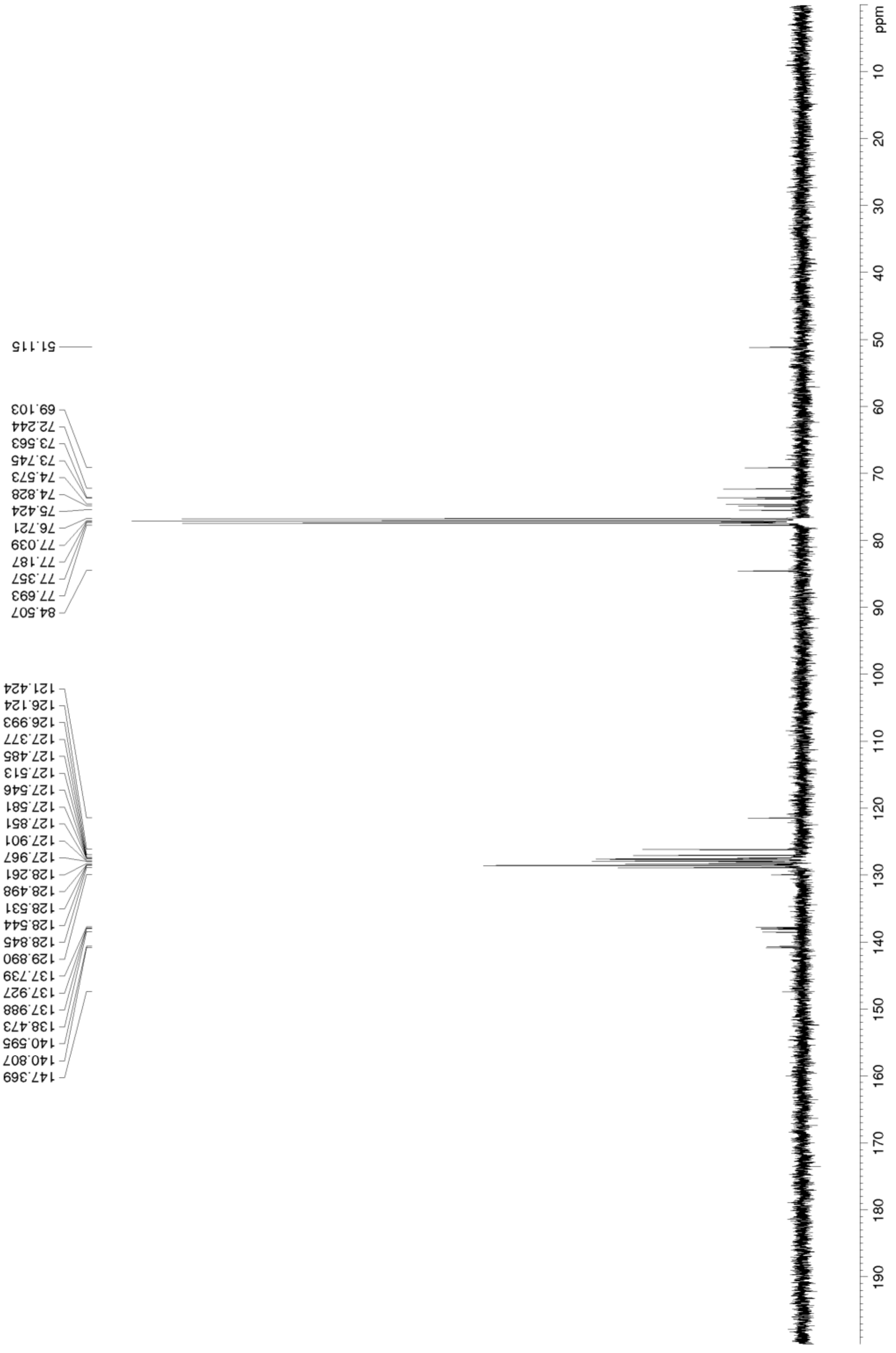
51.115
69.103
72.244
73.563
73.745
74.573
74.828
75.424
76.721
77.039
77.187
77.357
77.693
84.507
121.424
126.124
126.993
127.377
127.485
127.513
127.546
127.581
127.851
127.901
127.967
128.261
128.498
128.531
128.544
128.845
129.890
137.739
137.927
137.988
138.473
140.595
140.807
147.369
ppm
10
20
30
40
50
60
70
80
90
100
110
120
130
140
150
160
170
180
190

Compound **10n**:

^{1}H:

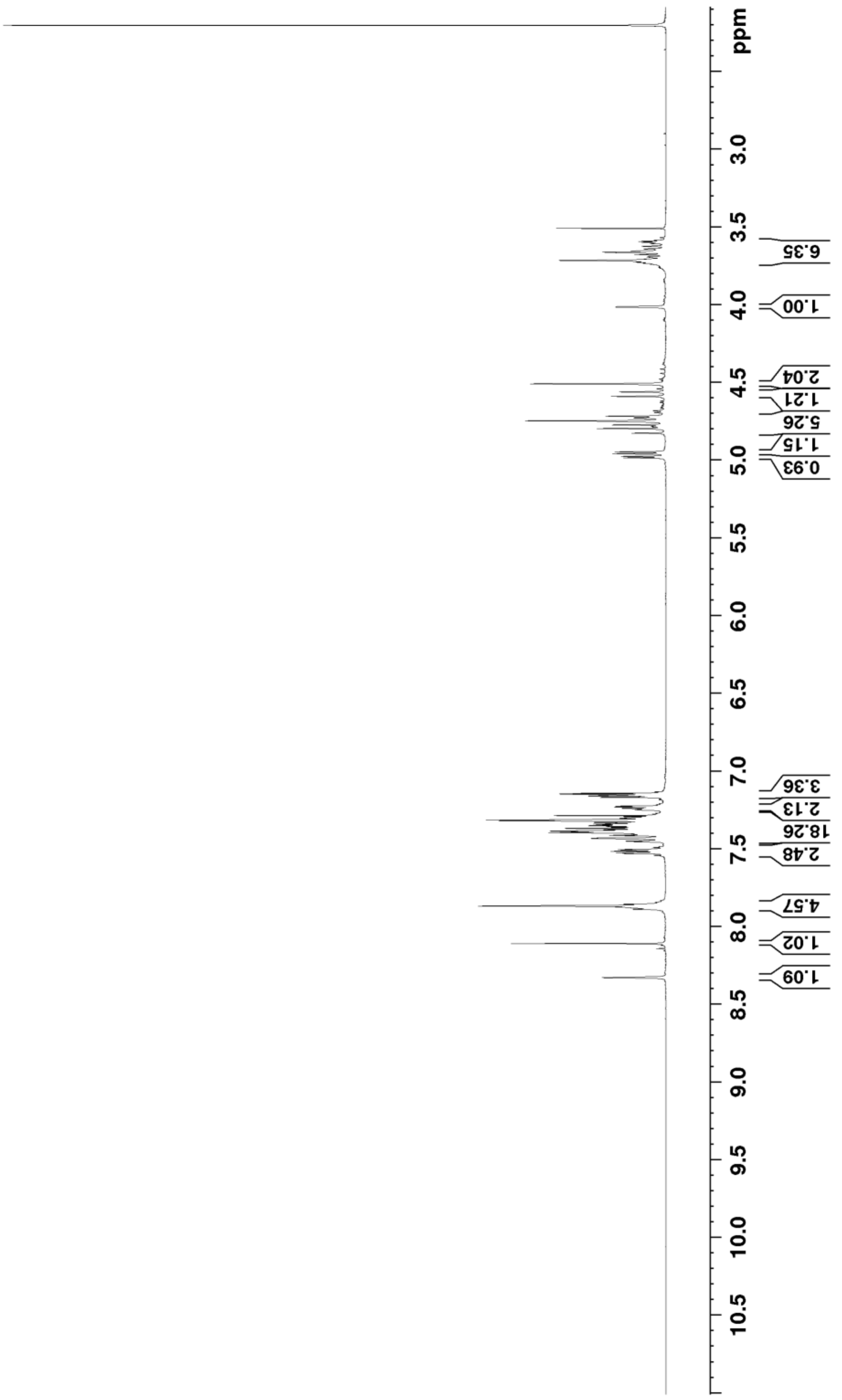

^{13}C:

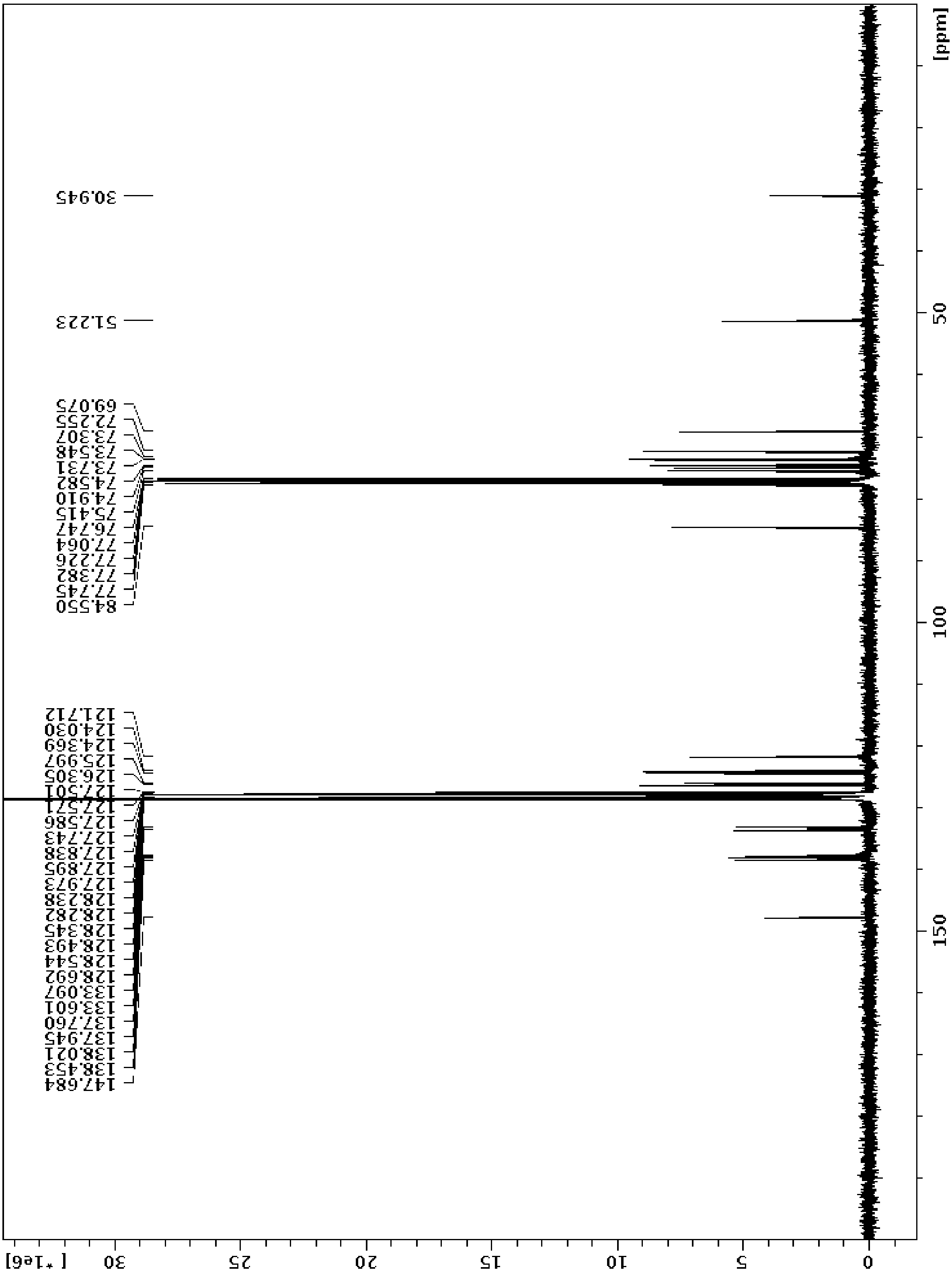
30.945
51.223
69.075
72.255
73.307
73.548
73.731
74.582
74.910
75.415
76.747
77.064
77.226
77.382
77.745
84.550
121.712
124.030
124.369
125.997
126.305
127.501
127.521
127.586
127.743
127.838
127.895
127.973
128.238
128.282
128.345
128.493
128.544
128.692
133.097
133.601
137.760
137.945
138.021
138.453
147.684
[ppm]
[*1e6]
